# Supramolecular Self‐Sorting Networks using Hydrogen‐Bonding Motifs

**DOI:** 10.1002/chem.201804791

**Published:** 2018-12-13

**Authors:** Heather M. Coubrough, Stephanie C. C. van der Lubbe, Kristina Hetherington, Aisling Minard, Christopher Pask, Mark J. Howard, Célia Fonseca Guerra, Andrew J. Wilson

**Affiliations:** ^1^ School of Chemistry University of Leeds Woodhouse Lane Leeds LS2 9JT UK; ^2^ Astbury Centre for Structural Molecular Biology University of Leeds Woodhouse Lane Leeds LS2 9JT UK; ^3^ Department of Theoretical Chemistry and Amsterdam Centre for Multiscale Modelling Vrije Universiteit De Boelelaan 1081 Amsterdam 1081 HV The Netherlands; ^4^ Leiden Institute of Chemistry, Gorlaeus Laboratories Leiden University Wassenaarseweg 76 Leiden 2333 AL The Netherlands

**Keywords:** biomimetic chemistry, hydrogen bonding, molecular recognition, self-sorting, supramolecular chemistry

## Abstract

A current objective in supramolecular chemistry is to mimic the transitions between complex self‐sorted systems that represent a hallmark of regulatory function in nature. In this work, a self‐sorting network, comprising linear hydrogen motifs, was created. Selecting six hydrogen‐bonding motifs capable of both high‐fidelity and promiscuous molecular recognition gave rise to a complex self‐sorting system, which included motifs capable of both narcissistic and social self‐sorting. Examination of the interactions between individual components, experimentally and computationally, provided a rationale for the product distribution during each phase of a cascade. This reasoning holds through up to five sequential additions of six building blocks, resulting in the construction of a biomimetic network in which the presence or absence of different components provides multiple unique pathways to distinct self‐sorted configurations.

## Introduction

Nature has the ability to assemble multiple functional assemblies simultaneously in defined locations and with temporal precision.^1^ For example, in a cellular signalling cascade, individual proteins interact selectively with certain proteins during one stage of a cascade and then with different proteins at another stage, driven by protein expression levels and enzymatically manipulated post‐translational modifications (PTMs).^1^, ^2^ Thus, regulatory control in a cellular context requires molecular recognition motifs capable of selective but adaptive recognition behaviour.^1^, ^3^ In terms of biomimetic systems that recapitulate these features, defined supramolecular assemblies employing multiple consecutive narcissistic (self‐loving) and/or social (self‐loathing) integrative self‐sorting^4^ events have been used to transition between well‐defined—and in some instances functional^5^—complexes.^6^ The parallel assembly of different complexes and transition to express different architectural complexity has also been demonstrated.5b However, these multicomponent systems have typically relied on shape and geometrical complementarity together with dative coordination bonds. In contrast, non‐integrative systems capable of transition between different self‐sorted configurations are less established as is the exploitation of weaker interactions, for example, hydrogen‐bonding set within the context of a recognition pattern of donors (D) and acceptors (A).^7^ Achieving this objective is a fundamental challenge and more accurately mimics many of the bimolecular associations that control cellular processes including those that occur within the context of multicomponent protein assemblies (e.g., the binding of co‐activators to transcription factor complexes).^8^

Previously, we have described a supramolecular system which we termed a self‐sorting cascade;^9^ it exploited both orthogonal and promiscuous recognition behaviour of linear arrays of hydrogen bonds^10^ to achieve sequential self‐sorting depending on which components were present. Comprising four different hydrogen‐bonding motifs, four rounds of self‐sorting could be achieved with different complexes formed depending on the sequence in which the components were added. Furthermore, the transitions could be triggered (albeit irreversibly) using a light‐mediated reaction to unmask one of the H‐bonding motifs, thus, mimicking the process of post‐translational regulation. In this work, we studied in greater detail the ability of linear arrays of hydrogen bonds to self‐sort using a combination of experimental (single‐crystal X‐ray diffraction and ^1^H NMR spectroscopy) and computational (DFT) methods. We illustrate that thermodynamically less preferential complexes can be formed during self‐sorting, driven by the stability of the entire system, and that this can be influenced by the configuration and conformation of the hydrogen‐bonding motif. This allowed us to extend the scope of the self‐sorting cascade to six components and construct cascades that operate in the presence of each other but with cross‐talk, recapitulating a new aspect of biological signalling and resulting in a self‐sorting network.

## Results and Discussion

**Figure 1:**
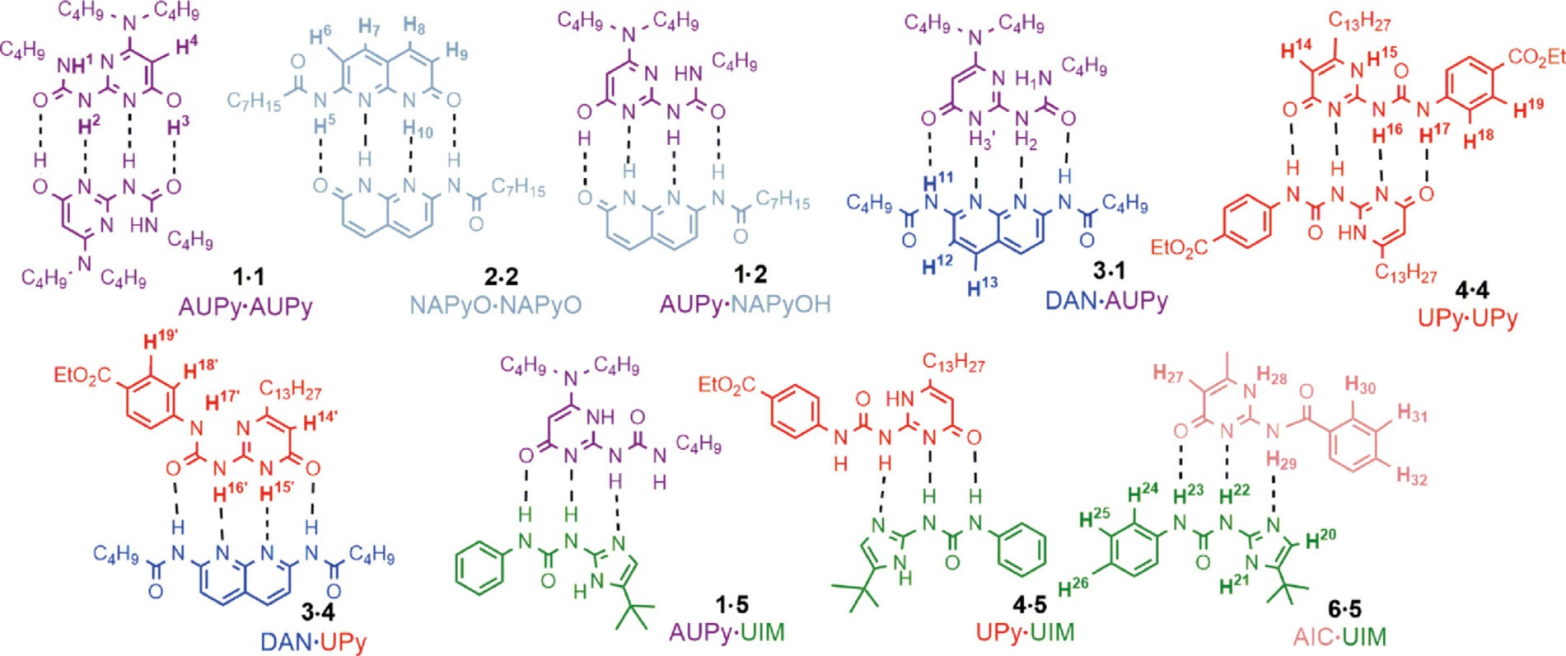
The lowest energy dimerisation interactions between the six linear hydrogen bonding motifs **1**–**6**, with dashed lines showing intermolecular hydrogen bonds.

In this work, we examined the literature to identify additional H‐bonding motifs **1**–**2** to add to those employed **3**–**6** in our previous study (Figure 1).^9^ Prior work by Sijbesma and co‐workers established that self‐complementary dialkylaminoureidopyrimidinone (AUPy) motifs, for example, **1**, and amidonapthyridone (NAPyO) motifs, for example, **2**, could be used to form alternating supramolecular copolymers through preferential heterocomplexation, with tuneable polymer composition.^11^ These building blocks were thus known to elicit low fidelity recognition behaviour in the same way as ureidopyrimidinone (UPy) **4**, an essential requirement for the assembly of a cascade.^12^ Such variable recognition behaviour arises through the different arrays of donors and acceptors (e.g., DADA, DDAA) presented by different accessible tautomers/conformers of each motif. The use of diamidonaphthyridine (DAN) **3**, ureidoimidazole (UIM) **5** and amidoisocytosine (AIC) **6**, with more restricted molecular recognition properties enforced by a solitary arrangement of donors and acceptors in each case, was also considered necessary for the effective assembly of a cascade. In the prior study, motifs **3**–**6** were shown to form a pair of tightly bound heterodimers **3⋅4** and **5⋅6** in chloroform, resulting from effective self‐sorting. Therefore, our study commenced with a deeper analysis of the recognition properties of these compounds with an emphasis on **1** and **2**.

### Synthesis of hydrogen bonding motifs

**Figure 2:**
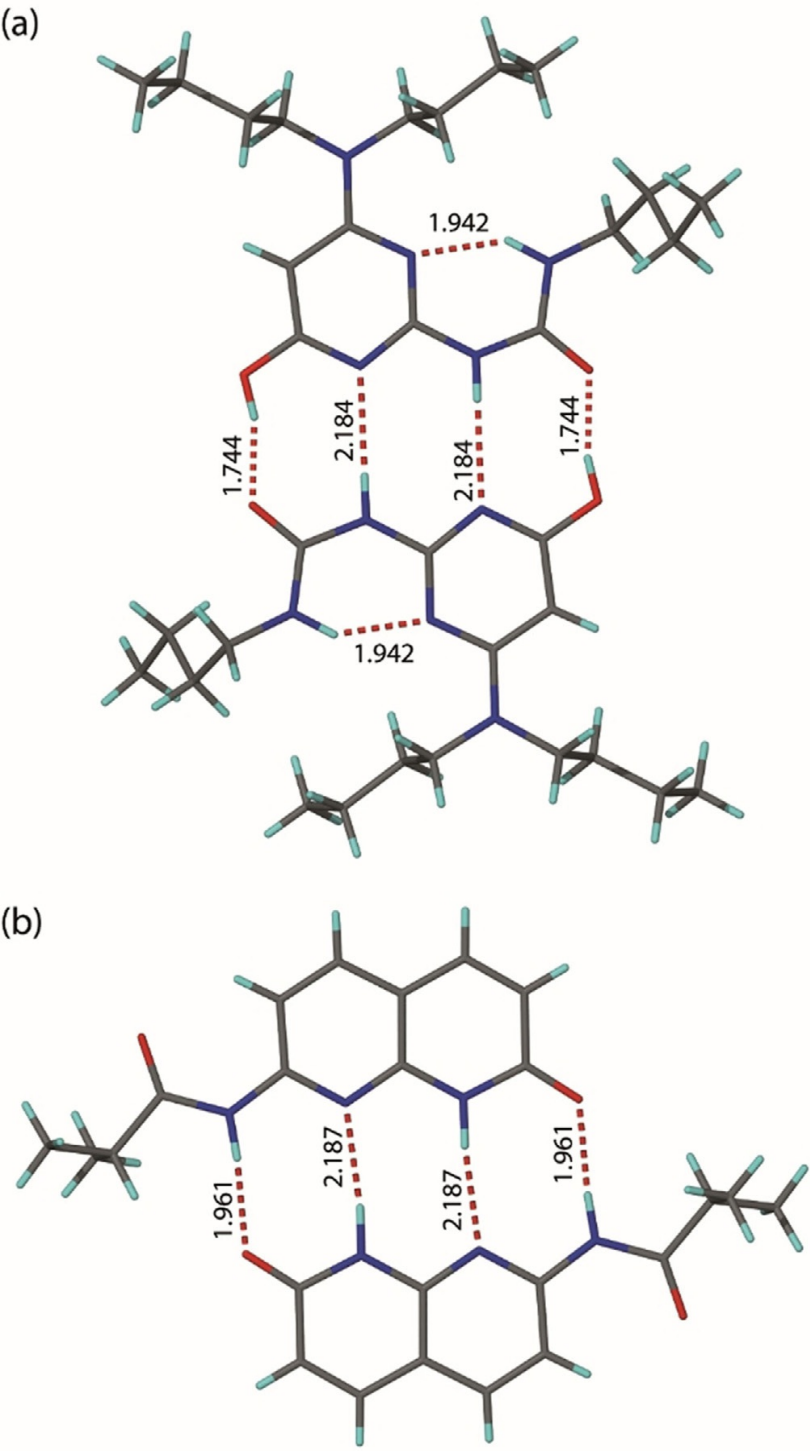
X‐ray crystal structures of a) AUPy **1** and b) NAPyO **7** homodimers (carbon is shown in grey, nitrogen in blue, oxygen in red, hydrogen in light blue and hydrogen bonds as dashed lines, with distances between the donor hydrogen and the acceptor expressed in Å).

The synthesis of AUPy **1** and NAPyO **2** was achieved by adaptation of previously described methods (see Experimental Section and Supporting Information).11b, 11c Like UPy **4**, AUPy **1** has the potential to adopt four different tautomers/conformers (Supporting Information, Figure S1 a), three of which present a quadruple hydrogen‐bond array. A single‐crystal analysis confirmed that AUPy **1** forms a quadruple hydrogen‐bonded homodimer **1⋅1** in the solid state; in this case with the monomer adopting the ADAD presenting tautomeric conformer (Figure 2 a). This is entirely consistent with results observed previously.11b Similarly, NAPyO **2** may feasibly access two tautomeric configurations (see Supporting Information, Figure S1b). Single‐crystal X‐ray analysis on an isopropyl analogue of NAPyO **7** revealed a quadruple hydrogen‐bonded homodimer **7⋅7** in which the monomer was present as the pyrimindinone tautomer, resulting in a DADA array (Figure 2 b).

### Pairwise interactions

The interactions of these two additional components (AUPy **1** and NAPyO **2**) with partners were then further investigated using ^1^H NMR spectroscopy in CDCl_3_. The ^1^H NMR resonances from separate samples of AUPy **1⋅1** and NAPyO **2⋅2** homodimers were compared to a 1:1 mixture of AUPy **1** and NAPyO **2** (Supporting Information, Figure S2). The small, yet notable, changes in the chemical shift of the NH signals relative to the homodimers were indicative of complex formation and consistent with reported interactions.11c Attempted 2D ^1^H–^1^H NOESY analyses were confounded by the presence of exchange peaks for NH/OH resonances, preventing a more in‐depth structural interpretation, although such exchange peaks are entirely consistent with an interaction between AUPy **1** and NAPyO **2**.

**Figure 3:**
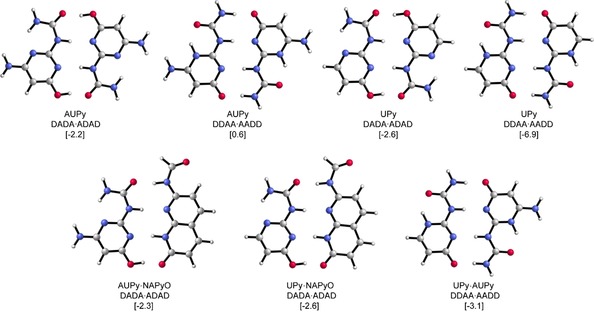
Optimised dimer interactions and their Gibbs free energies Δ*G* between brackets [in kcal mol^−1^] with respect to their most stable tautomer ADDA. Computed at the BLYP‐D3(BJ)/TZ2P level of theory (without alkyl chains).

To further confirm the presence of a heterodimer, variable‐temperature (VT) NMR analyses were performed (Supporting Information, Figure S3). As the temperature was reduced from 277 to 244 K, the broad NH/OH signals in the region 9–15 ppm sharpened and split to reveal additional resonances consistent with the presence of homodimers of AUPy **1⋅1** and NAPyO **2⋅2** as well as AUPy⋅NAPyO **1⋅2** heterodimer as the dominant species; at 253 K the ratio homodimer/heterodimer was observed to be 1:2. This indicates fast exchange between homodimers **1⋅1**/**2⋅2** and heterodimer **1⋅2** at room temperature. This is a property that is useful in the context of the cascades/network discussed later, in that recognition simply needs to translate into a response, for example, a signal change/distinct spectrum and not necessarily diagnostic resonances associated with particular complexes. To further understand the observations gleaned from ^1^H NMR analyses, we computed the Gibbs free energies of dimerisation (Δ*G*) at the BLYP‐D3(BJ)/TZ2P level of theory with implicit chloroform solvation at 298 K. The Gibbs free energies for the AUPy **1⋅1** and NAPyO **2⋅2** homodimers were −2.2 and −3.5 kcal mol^−1^, respectively (Figure 3, Table 1 and Supporting Information for Cartesian coordinates). The lowest energy AUPy⋅NAPyO **1⋅2** heterodimer was seen for the ADAD⋅DADA interacting array with a Gibbs free energy of −2.3 kcal mol^−1^. Hence, the dimerisation energy of the AUPy **1⋅1** homodimer is close to that of the AUPy⋅NAPyO **1⋅2** heterodimer, which is consistent with experimental observations of a mixture of homodimers and heterodimer at room temperature.

**Table 1:** The Gibbs free energies computed for all dimerisation interactions with respect to the most stable tautomer ADDA in implicit chloroform solvation at the BLYP‐D3(BJ)/TZ2P level of theory (without alkyl chains).

Hydrogen‐bonding motif	Interacting array	Δ*G* [kcal mol^−1^]
*Homodimers*		
AUPy	ADAD^[a]^	−2.2
AUPy	AADD	0.6
UPy	ADAD	−2.6
UPy	AADD^[a]^	−6.9
NAPyO	ADAD^[a]^	−3.5
NAPyO	AADD	7.4
*Heterodimers*		
AUPy⋅DAN	ADDA⋅DAAD^[a]^	−5.0
UPy⋅DAN	ADDA⋅DAAD^[a]^	−4.8
AUPy⋅NAPyO	ADAD⋅DADA^[a]^	−2.3
AUPy⋅NAPyO	AADD⋅DDAA	4.1
AUPy⋅NAPyO	ADDA⋅DAAD	0.0
UPy⋅NAPyO	ADAD⋅DADA	−2.6
UPy⋅NAPyO	AADD⋅DDAA	0.8
UPy⋅NAPyO	ADDA⋅DAAD	0.5
AUPy⋅UPy	ADAD⋅DADA	−2.4
AUPy⋅UPy	AADD⋅DDAA	−3.1

[a] Interacting array of dimers seen experimentally.

Next, the interactions of AUPy **1** and NAPyO **2** with UPy **4** were examined. Previous studies have highlighted the promiscuous nature of UPy **4** to interact through several hydrogen‐bonding arrays.^9^, ^12^ Surprisingly, ^1^H NMR spectroscopy suggested that there was no significant interaction of UPy **4** with AUPy **1** or NAPyO **2** in 1:1 mixtures (Supporting Information, Figures S4 and S5). A variable‐temperature ^1^H NMR analysis of a 1:1 mixture of NAPyO **2** and UPy **4** further confirmed narcissistic self‐sorting (supporting information Figure S6). In such mixtures, UPy **4** remained as homodimer **4⋅4** and did not show heterodimer interactions with AUPy (**1⋅4**) or NAPyO (**2⋅4**).

Taken together, these observations raised the following interesting points: 1) AUPy **1** has a preference to adopt a DADA pyrimidinol tautomer in the homodimer, in contrast to the extensively observed DDAA pyrimidinone tautomer in UPy **4**, and 2) AUPy **1** has the ability to form a heterodimer with NAPyO **2**, whereas UPy **4** does not. To resolve these queries, we turned to DFT calculations. These showed the most stable UPy **4⋅4** and AUPy **1⋅1** homodimers to have Δ*G* values of −6.9 and −2.2 kcal mol^−1^, respectively, whereas the most stable UPy⋅AUPy **1⋅4**, UPy⋅NAPyO **2⋅4** and AUPy⋅NAPyO **1⋅2** heterodimers had Δ*G* values of −3.1, −2.6 and −2.3 kcal mol^−1^, respectively (Figure 3). Hence, the dimerisation energy of UPy (i.e., to form **4⋅4**) contrasts with that of AUPy **1⋅1**, being considerably stronger than the potential heterodimers (**1⋅4** and **2⋅4**), which explained why AUPy forms heterodimers, whereas UPy does not.

Next, the molecular basis for the tautomeric difference between AUPy and UPy was reconciled. Naturally, the secondary electrostatic model proposed by Jorgensen,^13^ and verified by Zimmerman,^14^ dictates that the DDAA array should be a higher affinity interaction than the DADA array; nevertheless, the DADA tautomer is preferred by AUPy **1⋅1**. Our DFT calculations revealed that this is caused by the differences in tautomerization energy Δ*G*_taut_ (Supporting Information, Figure S7). When considering the Gibbs free energies of dimerisation with respect to the actual tautomer in the dimer (i.e., excluding Δ*G*_taut_) the AUPy **1⋅1** is more stable as DDAA than as DADA tautomer, and of similar energy to the calculated UPy **4⋅4** dimer. Thus, the higher energetic penalty of switching from the pyrimidinol to the pyrimidinone tautomer for AUPy is responsible for its preference to be in DADA tautomeric form. This may be attributed to the intramolecular hydrogen bond between the [6]N and ureido NH (Supporting Information, Figure S8).

**Figure 4:**
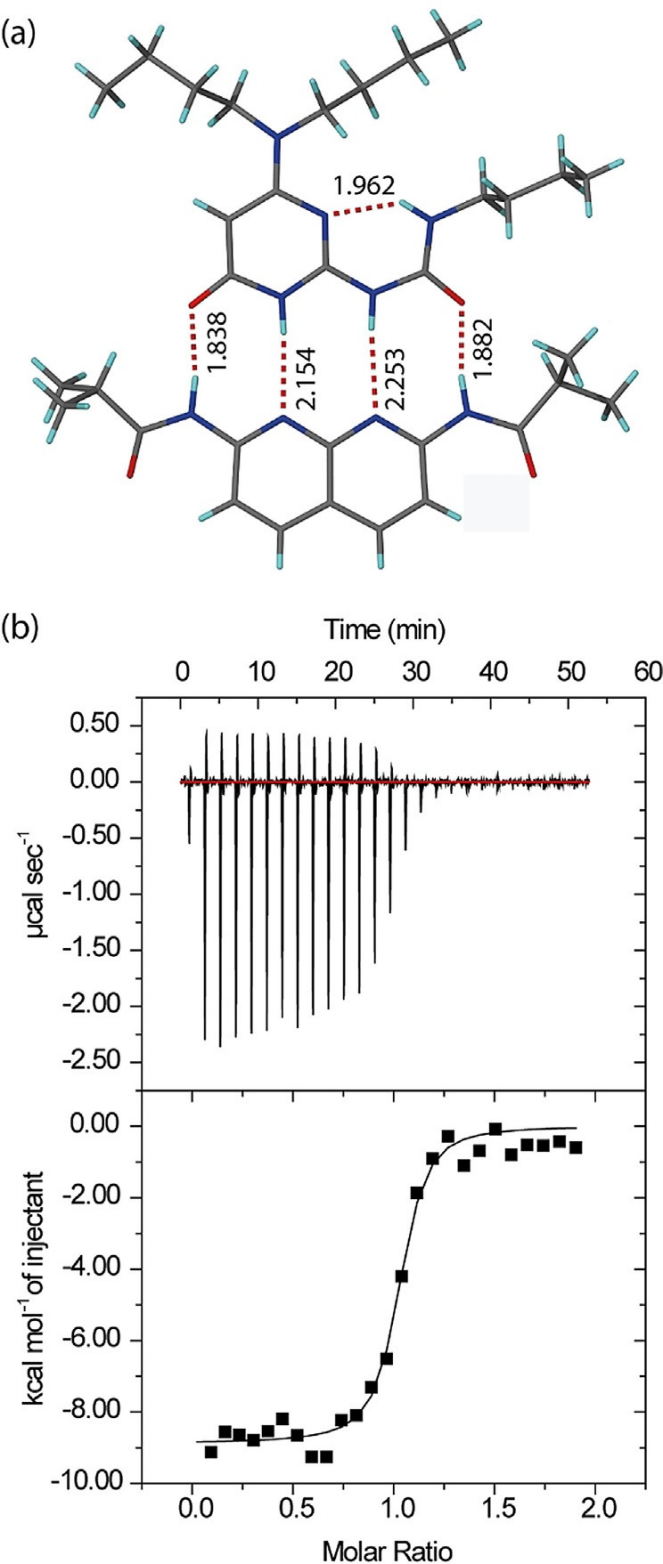
Structural and thermodynamic characterisation of AUPy⋅DAN **1⋅8** heterodimer. a) Single‐crystal structure of AUPy⋅DAN **1⋅8** heterodimer (carbon is shown in grey, nitrogen in blue, oxygen in red, hydrogen in light blue and hydrogen bonds as dashed lines, with distances between the donor hydrogen and the acceptor expressed in Å). b) ITC thermograms (top) and binding isotherm for the addition of a solution of AUPy **1** (1 mm) into DAN **3** (0.1 mm) in CHCl_3_ (bottom).

**Figure 5:**
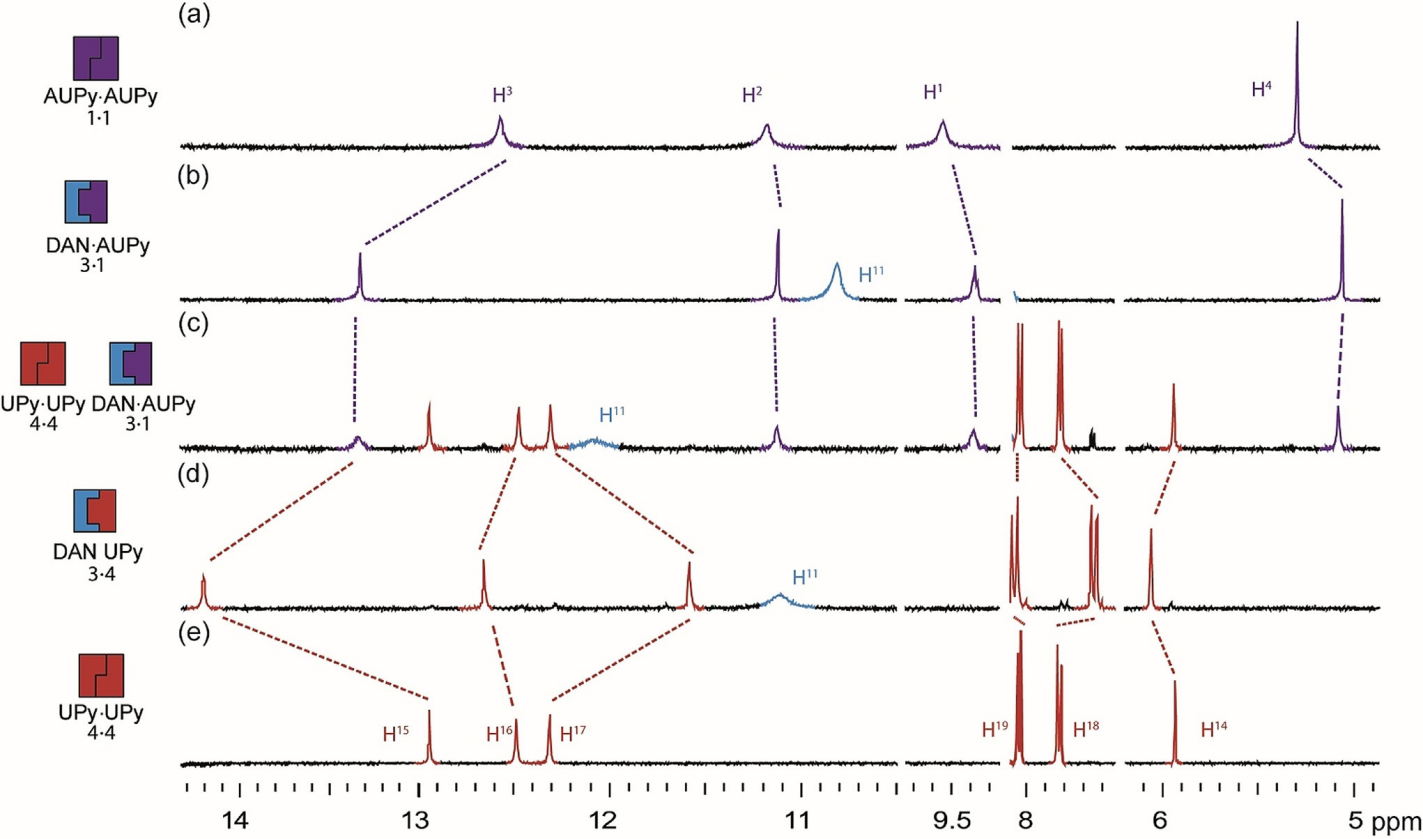
Analysis of pairwise interactions and preferences for AUPy **1**, DAN **3** and UPy **4** by ^1^H NMR spectroscopy (10 mm, CDCl_3_). a) AUPy **1**; b) 1:1 AUPy **1**: DAN **3**; c) 1:1:1 AUPy **1**: DAN **3**: UPy **4**; d) 1:1 DAN **3**: UPy **4**; (e) UPy **4**.

We then characterised the interaction of the additional components AUPy **1** and NAPyO **2** with DAN **3**, presenting only a DAAD arrangement of hydrogen‐bonding functionalities. ^1^H NMR analyses on 1:1 mixtures of NAPyO **2** and DAN **3** demonstrated no interaction, as expected given the incompatible linear arrays. In contrast, ^1^H NMR analyses on a 1:1 mixture of AUPy **1** and DAN **3** indicated heterodimer **1⋅3** formation (Supporting Information Figure S9), in a similar manner to the observation of heterodimer **3⋅4** formation for DAN **3** and UPy **4** (Supporting Information, Figure S10). 2D ^1^H–^1^H NOESY analyses also supported the formation of a heterodimer with NOE cross peaks between NH signals of both AUPy **1** and DAN **3** (Supporting Information, Figure S11). Additionally, single‐crystal X‐ray diffraction studies on a co‐crystal of AUPy **1** and DAN **8** (isopropyl analogue) revealed the anticipated ADDA⋅DAAD **1⋅8** heterodimer interaction, as expected (Figure 4 a). Isothermal titration calorimetry (ITC) confirmed a strong interaction with an association constant of *K*_a_=10^6^ m ^−1^ in chloroform (Figure 4 b). Next, we tested which dimers would “win‐out” when AUPy **1**, DAN **3** and UPy **4** were combined in a 1:1:1 ratio. The ^1^H NMR data were indicative of a mixture of UPy **4⋅4** homodimer and AUPy⋅DAN **1⋅3** heterodimer (Figure 5), consistent with prior observations.11c Our DFT analyses indicated no significant Gibbs free energy difference Δ*G* between UPy⋅DAN **3⋅4** (−4.8 kcal mol^−1^) and AUPy⋅DAN **1⋅3** (−5.0 kcal mol^−1^). Thus, the energetic difference between the two heterodimers fails to explain preferential AUPy⋅DAN (**1⋅3**) heterodimer formation. Instead, the behaviour is explained by considering the free energy change in this system and the additive effects of each dimer formed. Again, the high stability of the UPy **4⋅4** homodimer drove the behaviour of the system; considering that the Δ*G* for the UPy **4⋅4** homodimer is lower (i.e., more negative) than both heterodimers by 1.9 (AUPy⋅DAN **1⋅3**) and 2.1 (UPy⋅DAN **3⋅4)** kcal mol^−1^, UPy dimerises with itself instead of with DAN, and the AUPy **1⋅1** homodimer consumes two equivalents on DAN to form the AUPy⋅DAN **1⋅3** heterodimer. Although calculations revealed that AUPy⋅DAN **1⋅3** is 2.8 kcal mol^−1^ more stable than the AUPy **1⋅1** homodimer, the requirement to maximise hydrogen‐bonding interactions dictates this behaviour. To provide further evidence that the overall number of hydrogen bonds in the system must be maximised before considering the strength of the dimerisation interaction and lowest energy conformers, a further ^1^H NMR experiment was performed with DAN **3**, AUPy **1** and UPy **4** in 2:1:1 ratio (Supporting Information, Figure S12). This predictably resulted in an equal amount of AUPy**⋅**DAN **1⋅3** and UPy⋅DAN **4⋅3** heterodimers with no homodimers present. Here, the excess of DAN **3** drives the formation of UPy‐DAN **4⋅3** heterodimer over the UPy **4⋅4** homodimer, despite an energetic bias towards the latter.

Finally, the AUPy**⋅**DAN **1⋅3** heterodimer was also shown to “win‐out” in the presence of NAPyO **2**. Upon addition of DAN **3** to the weak AUPy⋅NAPyO **1⋅2** heterodimer, NAPyO **2** was displaced, resulting in the formation of AUPy⋅DAN **1⋅3** heterodimer and NAPyO **2⋅2** homodimer (Supporting Information, Figure S13). This can be readily explained in terms of both maximising the number of hydrogen bonds in a system and association strength. Unsurprisingly, DFT computations mirror these experimental observations; AUPy⋅NAPyO **1⋅2** and AUPy⋅DAN **1⋅3** heterodimers have a Δ*G* of −2.3 and −5.0 kcal mol^−1^.

### Self‐sorting cascades

**Figure 6:**
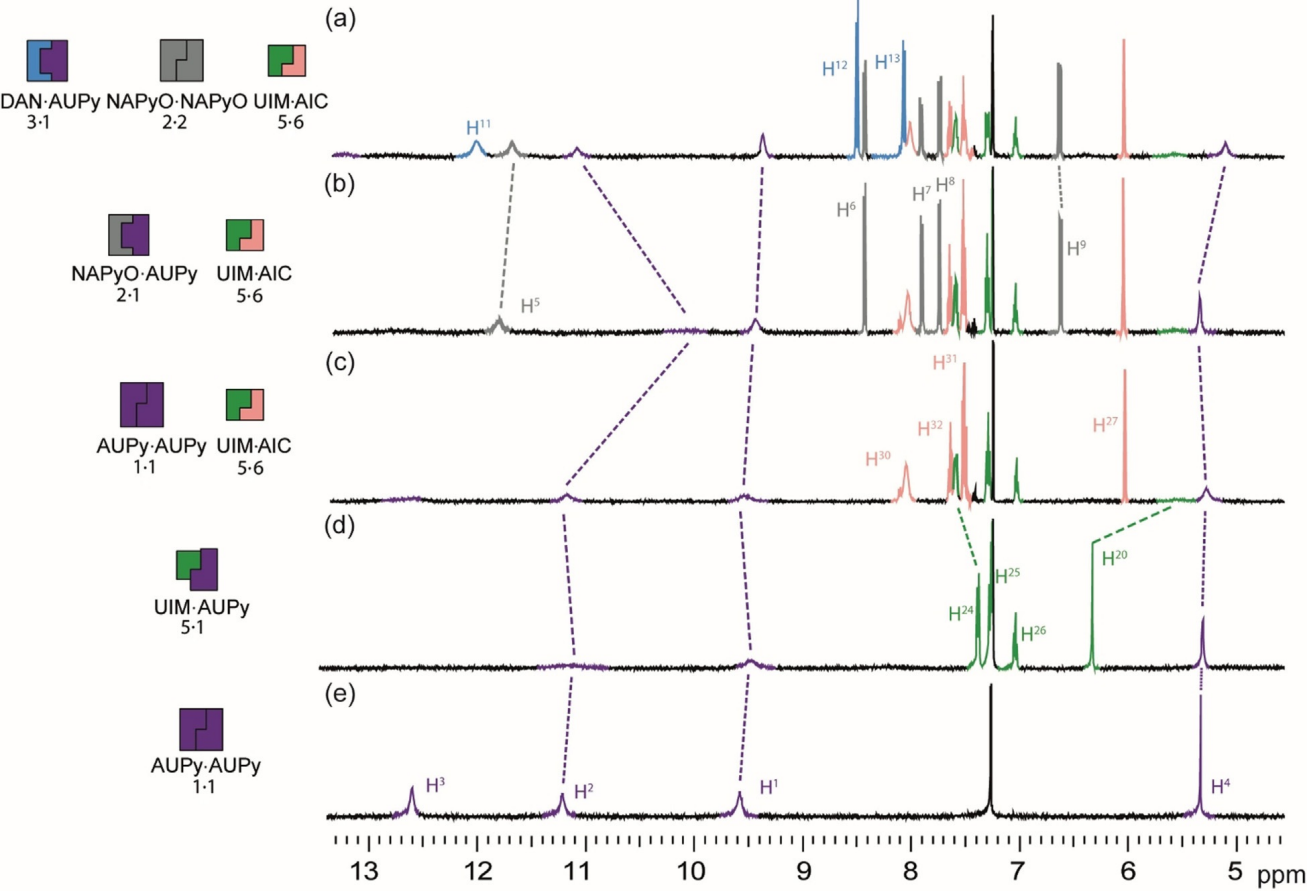
Five‐component signalling cascade studied by ^1^H NMR (500 MHz, 10 mm, CDCl_3_) (a) AUPy **1**, NAPyO **2**, DAN **3**, UIM **5** and AIC **6**; (b) AUPy **1**, NAPyO **2**, UIM **5** and AIC **6**; (c) AUPy **1**, UIM **5** and AIC **6**; (d) AUPy **1**, UIM **5**; (e) AUPy **1**.

Having characterised the pairwise dimerisation behaviour of the individual components, this allowed for the construction of a number of self‐sorting cascades. The simplest of which was conceived though the addition of NAPyO **2** to AUPy **1** followed by the addition of DAN **3**, as described above (Supporting Information, Figure S13*)*. More complex self‐sorting cascades exploiting five hydrogen‐bonding motifs were also investigated (Figure 6 a). Beginning with AUPy⋅AUPy **1⋅1** homodimer (Figure 6 e), the addition of UIM **5** (Figure 6 d) resulted in a small shift in the H^**4**^ resonance of AUPy **1** and broadening of the NH signals, consistent with AUPy⋅UIM **1⋅5** heterodimer formation (Supporting Information, Figure S14). The AIC **6** motif was then added to the mixture (Figure 6 c), resulting in the formation of UIM⋅AIC **5⋅6** indicated by the downfield shift in the H^24^ resonance and broadening of the H^20^ resonance of UIM **5** (Supporting Information, Figure S15). Concomitant recovery of the AUPy homodimer **1⋅1** was observed on the basis of the restoration of its NH resonances towards the expected frequency. Upon the addition of NAPyO **2** (Figure 6 b) the interaction between AIC**⋅**UIM **6⋅5** was unchanged, but small changes in chemical shifts of the NH resonances of AUPy **1** were observed, consistent with the formation of AUPy⋅NAPyO **1⋅2** as the dominant species comprising either components (as previously discussed). The cascade was completed by the addition of DAN **3** (Figure 6 a). The ^1^H NMR spectrum of AIC**⋅**UIM **6⋅5** heterodimer showed that no resonances indicative of this complex were affected. However, a significant upfield shift for proton H^4^ and sharpening of NH resonances for AUPy **1** indicated formation of AUPy⋅DAN **1⋅3** and the displacement of NAPyO **2** from complexation with AUPy **1** (Supporting Information, Figure S12). Based on this reasoning, the final mixture was comprised of AUPy⋅DAN **1⋅3** and AIC⋅UIM **6⋅5** heterodimers as well as NAPyO⋅NAPyO **2⋅2** homodimer (as predicted) and a clear pathway of sequential self‐sorting established for five components.

**Figure 7:**
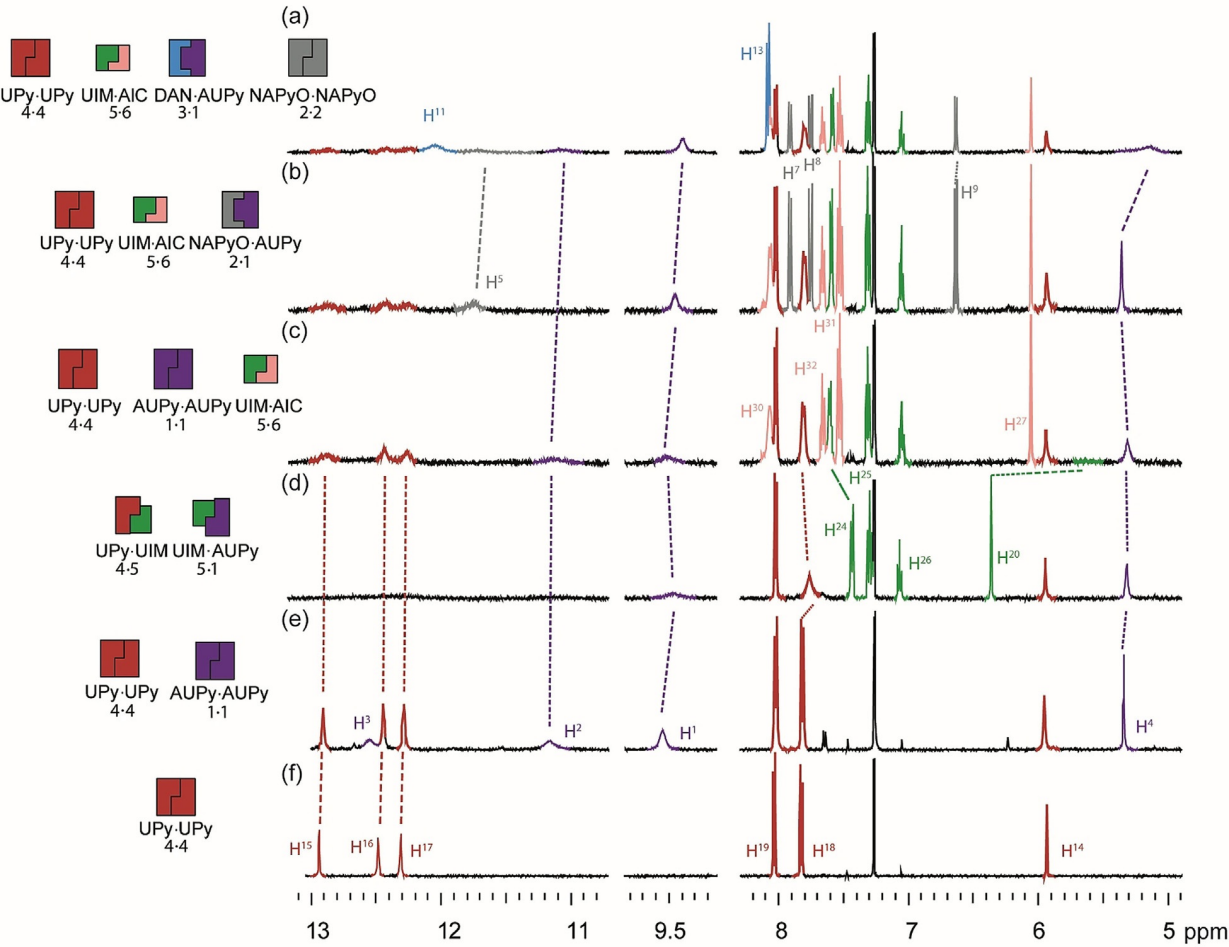
Pathway E. ^1^H NMR (500 MHz, 10 mm, CDCl_3_) six‐component signalling cascade a) AUPy **1**, NAPyO **2**, DAN **3**, UPy **4**, UIM **5**, and AIC **6**; b) AUPy **1**, NAPyO **2**, UPy **4**, UIM **5**, and AIC **6**; c) AUPy **1**, UPY **4**, UIM **5**, and AIC **6**; d) AUPy **1**, UPy **4**, and UIM **5**; e) AUPy **1** and UPy **4**; f) UPy **4**.

A series of ^1^H NMR experiments were then performed to exemplify a six‐component cascade (Figure 7). The first six‐component cascade (pathway E, see Figure 9) initiated with UPy **4⋅4** homodimer; addition of AUPy **1** led to no change in chemical shifts consistent with narcissistic self‐sorting of UPy **4⋅4** and AUPy **1⋅1** homodimers (Figure 7 e). Upon addition of UIM **5**, UPy⋅UIM **4⋅5** and AUPy⋅UIM **1⋅5** heterodimers formed as major products (Figure 7 d). This was indicated by the upfield shift and broadening of H^18^ of UPy **4** as well as by the upfield shift of H^4^ and broadening of NH signals of AUPy **1,** in line with heterodimerisation observed in the two‐component mixture (Supporting Information, Figures S14 and S16). Notably, distinct spectral changes are sufficient to distinguish this phase of the cascade from the preceding and subsequent phases, demonstrating that the absence of clear speciation may be tolerated in cascades (see earlier discussion on AUPy⋅NAPyO **1⋅2**). AIC **6** was then added (Figure 7 c), resulting in a strong heterodimer formation with UIM **5** (illustrated by the downfield shift of H^2^, Supporting Information, Figure S14), and simultaneous reformation of the UPy **4⋅4** and AUPy **1⋅1** homodimers. Formation of the AUPy⋅NAPyO **1⋅2** heterodimer occurred on addition of NAPyO **2** to the cascade in the presence of UPy **4⋅4** homodimer and UIM⋅AIC **5⋅6** heterodimers (Figure 7 b). Addition of DAN **3**, as expected, disrupted the **1⋅2** complexation resulting in AUPy⋅DAN **1⋅3** heterodimer and NAPyO **2⋅2** homodimers (Figure 7 a). Thus, a final product distribution with four major components AUPy⋅DAN **1⋅3**, UIM⋅AIC **5⋅6** heterodimers and NAPyO **2⋅2**, UPy **4⋅4** homodimers was observed.

### Orthogonal self‐sorting cascade

**Figure 8:**
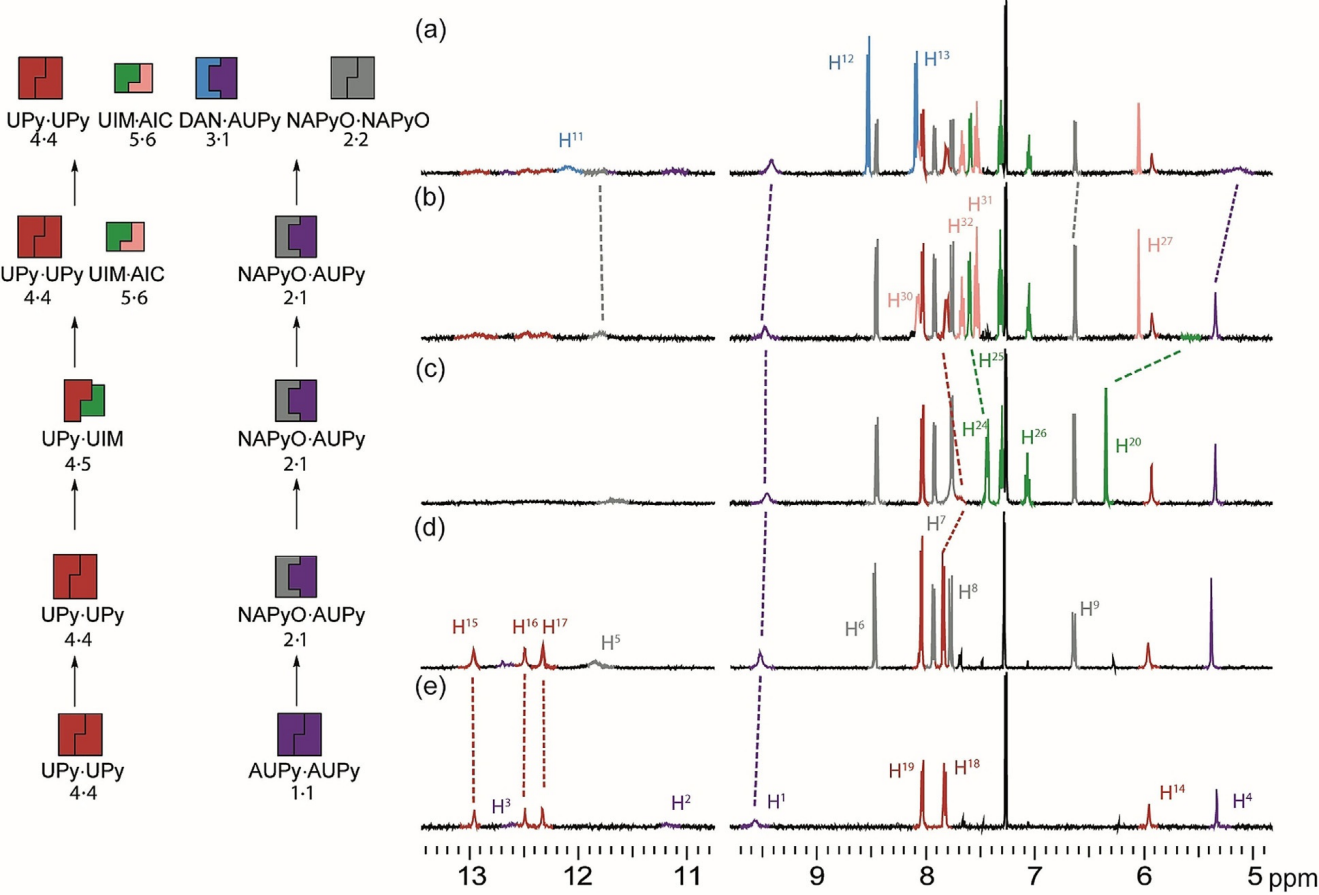
Pathway G. ^1^H NMR (500 MHz, 10 mm, CDCl_3_) orthogonal signalling cascade a) AUPy **1**, NAPyO **2**, DAN **3**, UPy **4**, UIM **5**, and AIC **6**; b) AUPy **1**, NAPyO **2**, UPy **4**, UIM **5**, and AIC **6**; c) AUPy **1**, NAPyO **2**, UPY **4** and UIM **5**; d) AUPy **1**, NAPyO **2** and UPy **4**; e) AUPy **1** and UPy.

To develop cascades occurring in parallel (but comprising components capable of cross‐talk), an approach to perform two orthogonal self‐sorting cascades (pathway G, Figure 8) was developed. The aim was to create two parallel cascades that can operate in sequence, in spite of the ability of complexes from both cascades to interact with one another in different scenarios. Starting with a mixture of UPy **4⋅4** and AUPy **1⋅1** homodimers (Figure 8 e), the cascade could be split in two with UPy **4⋅4** at the head of one channel and AUPy **1⋅1** at the head of the other. When NAPyO **2** was added (Figure 8 d), the “UPy channel” was unchanged, whereas AUPy **1** interacted with NAPyO **2** to form AUPy⋅NAPyO **1⋅2** heterodimer. Upon addition of UIM **5** to this three‐component mixture (Figure 8 c), the “AUPy channel” was unchanged and UPy⋅UIM **4⋅5** heterodimer formed in the UPy channel. With the addition of AIC **6** (Figure 8 b), the “AUPy channel” was unchanged through a further stage but, in the “UPy channel”, UPy⋅UIM **4⋅5** heterodimer was disrupted to form UPy **4⋅4** homodimer and UIM⋅AIC **5⋅6** heterodimer. The cascade was completed with the addition of DAN **3**; in this case, the “UPy channel” was unchanged but DAN **3** interacted with AUPy **1** in the “AUPy channel” to form AUPy⋅DAN **1⋅3** heterodimer liberating NAPyO **2⋅2** homodimer. Overall, four social self‐sorting transitions occurred, two in one channel and two in the other; however, the transitions did not cross over resulting in orthogonal cascades.

### Self‐sorting network

**Figure 9:**
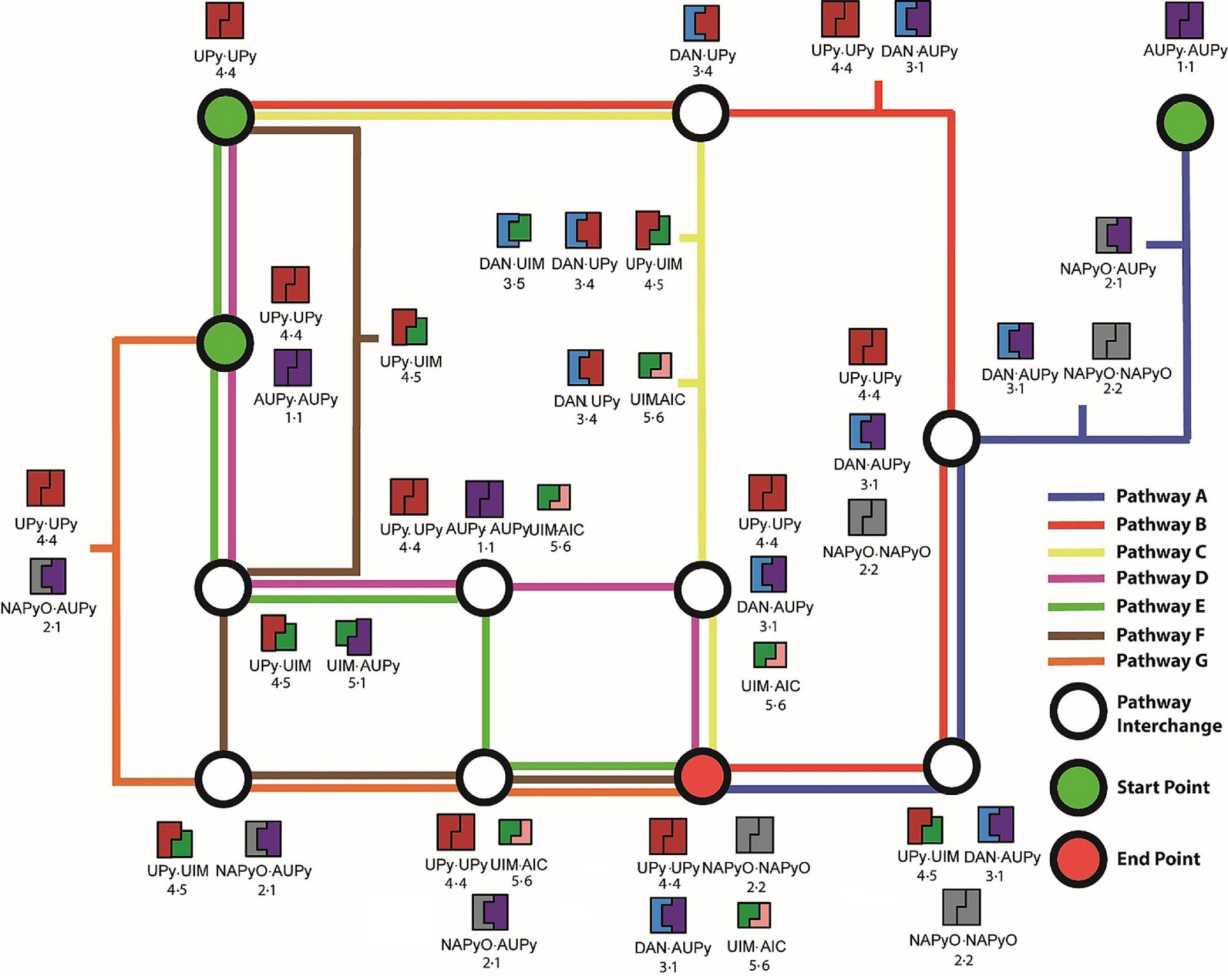
Schematic showing the self‐sorting behaviour of pathways A–G in forming hydrogen‐bonding dimers.

To elaborate a self‐sorting network, several different additional cascades were exemplified; understanding potential hydrogen‐bonding interactions between motifs **1**–**6** was key in creating a self‐sorting network with intersecting pathways (Figure 9). By comparing the ^1^H NMR spectra of each mixture with the distinct chemical shifts of heterodimers and homodimers, the major components in each pathway were identified. Hence, several different self‐sorting pathways could be plotted together based on their product distribution, resulting in a network of diverging and converging paths with the same end point. For example, pathway B (red, Figure 9) initiated with UPy **4⋅4** homodimer and, upon addition of DAN **3**, led to production of DAN⋅UPy **3⋅4** heterodimer (Supporting Information, Figure S18 e). Pathway B continued with the addition of AUPy **1**, resulting in disassembly of DAN⋅UPy **3⋅4** heterodimer at the expense of DAN⋅AUPy **3⋅1**, together with regeneration of UPy **4⋅4** homodimer (Supporting Information, Figure S18 d). The hydrogen‐bonding interactions were unchanged on the addition of NAPyO **2**, which was present as the **2⋅2** homodimer (Supporting Information, Figure S18c). UIM **5** was able to interfere with the UPy **4⋅4** homodimer interactions to form UPy⋅UIM **4⋅5** heterodimer (Supporting Information, Figure S18 b), which was in turn disassembled to form UIM⋅AIC **5⋅6** heterodimer with concomitant UPy **4⋅4** homodimer formation on addition of AIC **6** (Supporting Information, Figure S16 a). Pathway C (yellow, Figure 9) took a different route from pathway B, in that UIM **5** and AIC **6** are successively added to the DAN⋅UPy **3⋅4** heterodimer (considered here as an interchange) to create UPy⋅UIM **4⋅5** and UIM⋅AIC **5⋅6** heterodimers, respectively (Supporting Information, Figure S19 c,d). The ability of UIM **5** to disrupt the DAN⋅UPy **3⋅4** heterodimer is moderate and a series of low‐fidelity complexes formed. Nonetheless, the resultant ^1^H NMR spectrum was diagnostic of a distinct state within the network, and, the addition of AIC **6** restored a well‐resolved spectrum indicative of a well‐defined self‐sorted configuration. Finally, in pathway C, addition of AUPy **1** switched the configuration from DAN⋅UPy **3⋅4** and UIM⋅AIC **5⋅6** to AUPy⋅DAN **1⋅3**, UPy **4⋅4** and UIM⋅AIC **5⋅6** with subsequent addition of NAPyO **2⋅2** promoting no change in the distribution of the other components (Supporting Information, Figure S19 a,b). Pathways A, D, E and F further exemplify the different network configurations that can be obtained depending on which components are present and, therefore, expressed in the system (see Supporting Information, Figures S19–S21 for experimental data).

## Conclusion

Detailed analysis of molecular recognition behaviour of hydrogen‐bonding motifs by experiments and computations allowed for a complex self‐sorting network made up of several signalling cascades to be created. The sequential addition of six linear hydrogen motifs led to cascades capable of both narcissistic and social self‐sorting phases as well as a mixture of both. Through examination of the interactions between the individual components of each cascade, the product distribution of the overall system can be understood in terms of orthogonal recognition. The varying degrees of fidelity and promiscuity of the hydrogen‐bonding interactions of these motifs and critically an understanding of their behaviour was essential in developing the cascades comprising the network. These cascades include for the first‐time parallel cascades that operate in the presence of each other, but which are capable of cross‐talk. Future studies will centre on exploiting these motifs as components of self‐sorting and reconfigurable materials, and on developing reversible transitions between the different phases of the cascade. Beyond this, developing approaches to temporarily perturb the system may provide access to out‐of‐equilibrium networks with emergent behaviour.

## Experimental Section

**General considerations**: Solvents and reagents were purchased from Sigma Aldrich or Fisher Scientific and used without further purification unless otherwise stated. Where anhydrous solvents were required, dichloromethane, chloroform, tetrahydrofuran and acetonitrile were obtained from the in‐house solvent purification system Innovative Inc. PureSolv®. Anhydrous pyridine was placed over KOH for 24 hours before being refluxed for 2 hours and distilled over Linde 5 Å molecular sieves and solid KOH before use. All non‐aqueous reactions were carried out under a nitrogen atmosphere. Chloroform‐d was placed on CaCl_2_ before being distilled over Linde 5A molecular sieves before use in ^1^H NMR cascade experiments. Analytical thin layer chromatography was performed on Merck Kieselgel 60 F_254_ 0.25 mm pre‐coated aluminium plates. Product spots were visualised under UV light ((*λ*_max_=254 nm). Flash chromatography was carried out using Merck Kieselgel 60 silica gel. Nuclear magnetic resonance spectra were obtained at 298 K (unless stated) using a Bruker AV500 spectrometer operating at 11.4 T (500 MHz for ^1^H) and JEOL ECA600ii operating at 14.1 T (150 MHz for ^13^C) and NOESY spectra as stated. Infra‐red spectra were obtained using a PerkinElmer FTIR spectrometer in which absorption maxima ($\tilde{\nu }$_max_) are expressed in wavenumbers (cm^−1^) and only structurally relevant absorptions have been included. High‐Resolution mass spectra were recorded with a BrukerDaltonicsmicroTOF using electrospray ionisation (ESI). Detailed synthetic procedures and characterization are given in the Supporting Information.

**Crystal structure determination for 1**: Single crystals were grown by the slow evaporation of **1** in acetonitrile. X‐Ray diffraction data were collected at the University of Leeds. Crystal data. C_17_H_31_N_5_O_2_; M=337.47; crystal size 0.21×0.07×0.04 mm; triclinic; space group P$\tilde{1}$ ; *a*=5.1924(6), *b*=11.8545(15), *c*=15.3976(10) Å, *α*=97.911(8), *β*=93.859(7), *γ*=102.021(10)°; *V*=913.65(17) Å^3^; *T*=120.3(7) K; *Z*=2; *l*=0.661 mm^−1^; *λ*=1.54184 Å [Cu‐Kα]; 6466 reflections measured; 3410 unique reflections (*R*_int_=0.0462); observed *I*>2*r*(*I*). The final *R*_1_ was 0.0532 (observed reflections 0.0774) and *w*R(F^2^) was 0.1306 (all data 0.1468) for 232 parameters.

**Crystal structure determination for 7**: Single crystals were grown by the slow evaporation of **7** in acetonitrile. X‐Ray diffraction data were collected at the University of Leeds. Crystal data. C_12_H_13_N_3_O_2_, M=231.25; crystal size 0.15×0.06×0.03 mm; monoclinic; space group *P*2_1_/*n*; *a*=4.8837(11), *b*=22.962(4), *c*=9.9321(18) Å; *α*=90, *β*=101.26(2), *γ*=90°; *V*=1092.4(4) Å^3^; *T*=120.00(10) K; *Z*=4; *l*=0.811 mm^−1^; *λ*=1.54184 Å [Cu‐Kα]; 4018 reflections measured; 2121 unique reflections (*R*_int_=0.0500); observed *I*>2*r*(*I*). The final *R*_1_ was 0.0832 (observed reflections 0.1048) and *wR*(F^2^) was 0.2057 (all data 0.2223) for 164 parameters.

**Crystal structure determination for 1⋅8**: Single crystals were grown by slow evaporation of a 1:1 mixture of **1** and **8** in acetonitrile. X‐Ray diffraction data were collected at the University of Leeds. Crystal data. C_33_H_51_N_9_O_4_, M=637.82; crystal size 0.29×0.11×0.08 mm; monoclinic; space group *P*2_1_/*n*; *a*=9.52157(15), *b*=15.8558(3), *c*=22.3751(3) Å; *α*=90, *β*=92.0659(13), *γ*=90°; *V*=3375.81(9) Å^3^; *T*=119.99(13) K; *Z*=4; *l*=0.684 mm^−1^; *λ*=1.54184 Å [Cu‐Kα]; 13 422 reflections measured; 6628 unique reflections (*R*_int_=0.0348); observed *I*>2*r*(*I*). The final *R*_1_ was 0.0412 (observed reflections 0.0541) and *wR*(F^2^) was 0.0996 (all data 0.1084) for 442 parameters.

**Crystallographic data**: CCDC 1868220 (**1**), 1868221 (**7**), and 1868219 (**1⋅8**) contain the supplementary crystallographic data for this paper. These data are provided free of charge by The Cambridge Crystallographic Data Centre.

**NOESY data acquisition and processing**: Phase sensitive ^1^H–^1^H NOESY experiments were performed on a 1:1 mixture of components at 50 mm concentration with respect to each component in CDCl_3_. Spectra were recorded using a 750 ms mixing time with 256 increments, 2048 data points on a JEOL ECA600ii spectrometer at 298 K operating at 14.1 T (600 MHz for ^1^H).

**ITC experiments**: ITC experiments were carried out using Microcal ITC200i instrument (Malvern) at 25 °C in chloroform. 100 μm DAN **3** was present in the cell and titrated with 1 mm AUPy **1** loaded into the syringe using 26 1.4 μL injections with 120 s spacing between the injections. Heats of chloroform dilution was subtracted from each measurement raw data. Data was analysed using Microcal Origin 8 and fitted to a one‐binding site model.

**Molecular structure calculations**: The computations were carried out by using the density functional theory‐based program Amsterdam Density Functional (ADF) 2017.208^15^ at the BLYP‐D3(BJ)/TZ2P^16^ level of theory, which is known to accurately reproduce hydrogen‐bond strengths and lengths.^17^ Solvent effects were accounted for by using the implicit conductor‐like screening model (COSMO), in which the solute molecule is surrounded by a dielectric medium.^18^ All optimised structures have been verified to be true minima (zero imaginary frequencies). The molecular figures were illustrated using CYLview.^19^ Full computational details are given in the Supporting Information.

^**1**^ **H NMR experiments pairwise analysis**: The one‐component and two‐component ^1^H NMR experiments were performed on separate samples. The mass of each component was calculated to make a final concentration of 10 mm in 0.6 mL of CDCl_3_ (dried as described previously10f). The required mass of each component was dissolved in 0.6 mL of CDCl_3_. The sample was allowed to equilibrate for a minimum of ten minutes before acquisition. The acquired spectra of the one‐component and two‐component mixtures were compared for changes in the chemical shifts of specific resonances, indicative of hydrogen bonding.

^**1**^ **H NMR experiments cascades**: The mass of each component was calculated to make a final concentration of 10 mm in 0.6 mL of CDCl_3_. The required mass of each component for the first step of the cascade was dissolved in 0.6 mL of CDCl_3_. The sample was allowed to equilibrate for a minimum of ten minutes before acquisition. The required mass of the next component in the cascade was added to the same sample. The sample was allowed to equilibrate for a minimum of ten minutes before acquisition. The remaining components were added in the same sequential manner after each acquisition until all the components were added and the ^1^H NMR spectra was acquired for each stage of the cascade.

## Conflict of interest

The authors declare no conflict of interest.

## Supporting information

As a service to our authors and readers, this journal provides supporting information supplied by the authors. Such materials are peer reviewed and may be re‐organized for online delivery, but are not copy‐edited or typeset. Technical support issues arising from supporting information (other than missing files) should be addressed to the authors.

Supplementary
