# Supplementary material for: Supramolecular Self‐Sorting Networks using Hydrogen‐Bonding Motifs
Source: Chemistry. 2018 Dec 13;25(3):785–95. doi: 10.1002/chem.201804791 (PMC6563691; doi:10.1002/chem.201804791)
Supplement: Supplementary file 1 — Supplementary [file CHEM-25-785-s001.pdf]

CHEMISTRY
A European Journal

Supporting Information

Supramolecular Self-Sorting Networks using Hydrogen-Bonding Motifs

Heather M. Coubrough,[a, b] Stephanie C. C. van der Lubbe,[c] Kristina Hetherington,[a, b] Aisling Minard,[a, b] Christopher Pask,[a] Mark J. Howard,[a] Célia Fonseca Guerra,*[c, d] and Andrew J. Wilson*[a, b]

chem_201804791_sm_miscellaneous_information.pdf

Contents

1. Supporting Information Figures 2

2. Synthetic Procedures 16

2-amino-6-(dibutylamino) 4-pyrimidinol 16

1-butyl-3-(4-(dibutylamino)-6-hydroxypyrimidin-2-yl)urea 16

7-Amino-1,8-naphthridin-2-ol 17

2-ethyl-*N*-(5methyl-1,5-naphtyridine)hexanamide 17

7-[(propan-2-yl)amino]-1,8-naphthyridin-2-ol 18

***N*2,*N*7-bis(propan-2-yl)-1,8-naphthyridine-2,7-diamine** 18

***N-tert*-Butoxycarbonylguanidine** 18

***Tert*-Butyl 5-*tert*-1*H*-imidazol-2-yl-carbamate** 19

***4-tert*-Butyl-1*H*-imidazole-2-amine hydrochloride** 19

1-(4-*tert*-Butyl-1*H*-imidazol-2-yl)-3-phenylurea 19

Benzamido-5-methylisocytosine 20

3. Spectral Data 21

4. Computational Data 27

Voronoi deformation density (VDD) charges 28

Cartesian coordinates monomers 29

Cartesian coordinates dimers 33

References 42

1. Supporting Information Figures

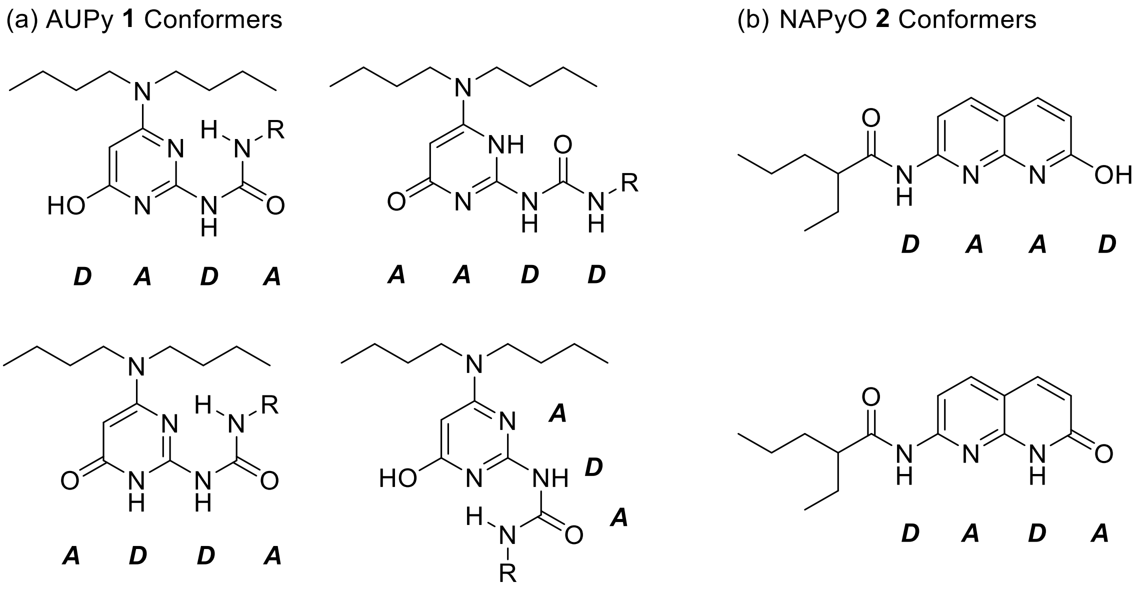

Figure S1. Different tautomeric configurations and conformations accessible by linear hydrogen-bonding arrays **1** and **2** (a) Four possible conformers of AUPy **1** (b) Two possible conformers of NAPyO **2**.

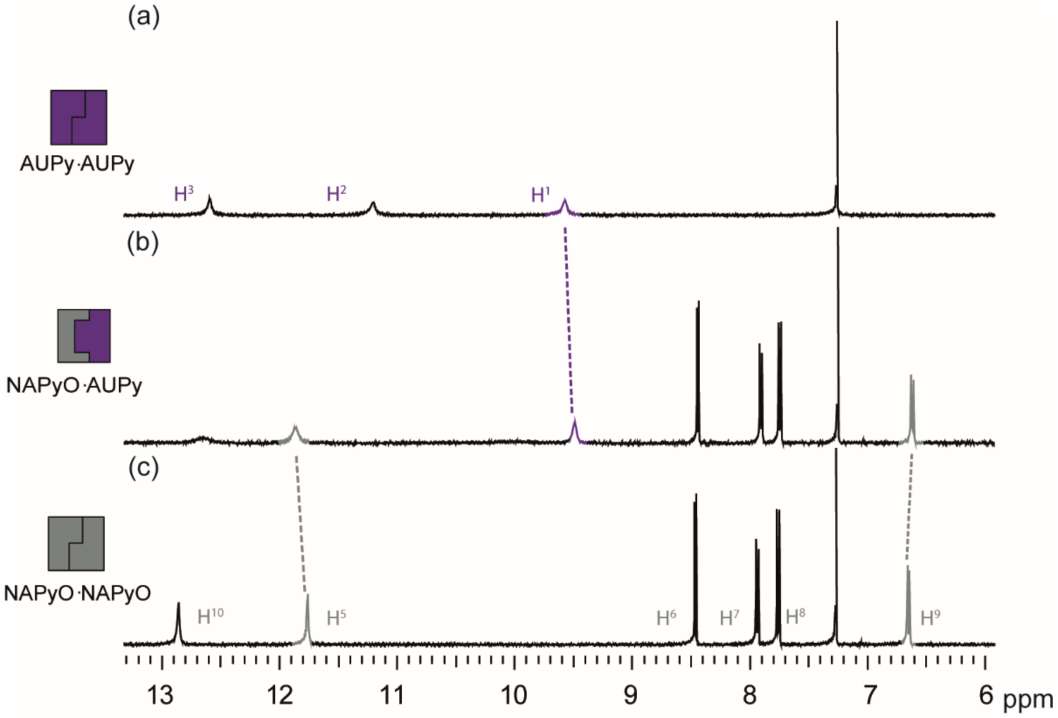

Figure S2. ^{1}H NMR (500 MHz, 10 mM, $CDCl_3$) for (a) AUPy **1·1** (b) 1:1 NAPyO·AUPy **1·2** and (c) NAPyO **2·2**.

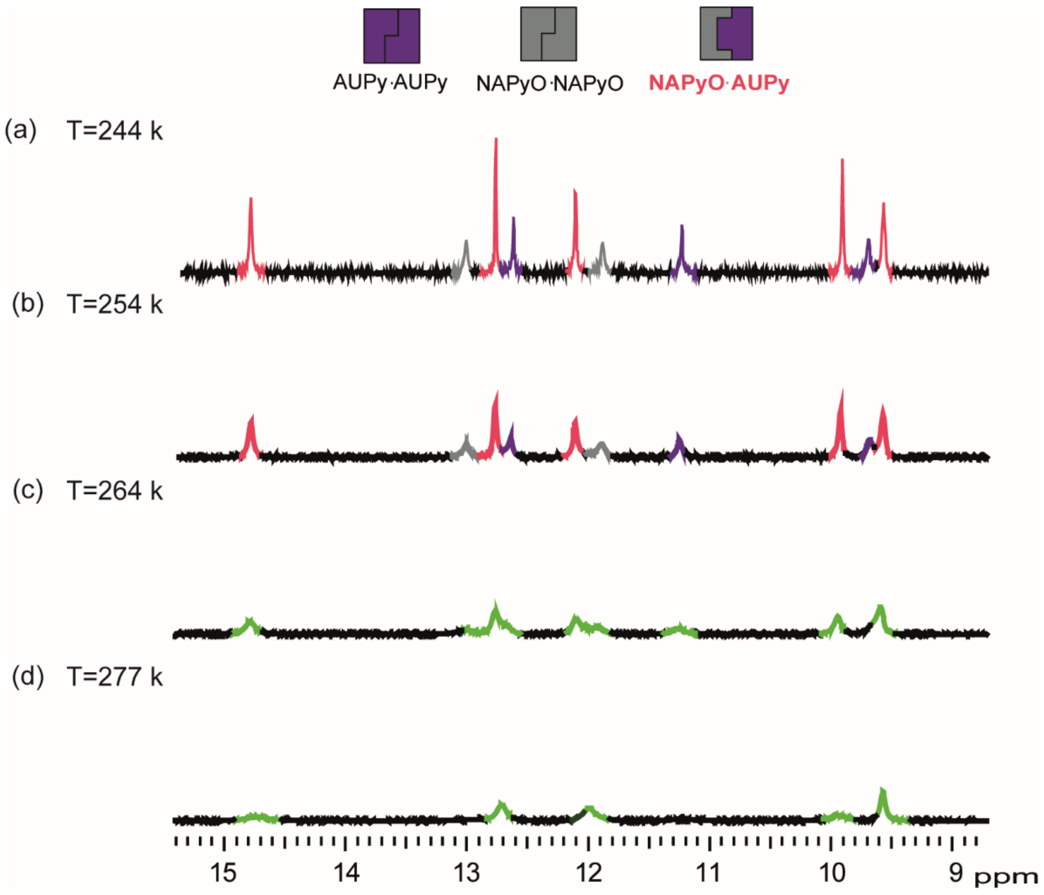

Figure S3. Variable temperature ^1H NMR (500 MHz, 10 mM, $CDCl_3$) of 1:1 AUPy **1** and NAPyO **2** at (a) 244 K (b) 254 K (c)264 K (d) 277 K. Signals colour code; green = mixture of complexes, purple = AUPy homodimer **1·1**, grey = NAPyO homodimer **2·2**, pink = AUPy·NAPyO **1·2** heterodimer.

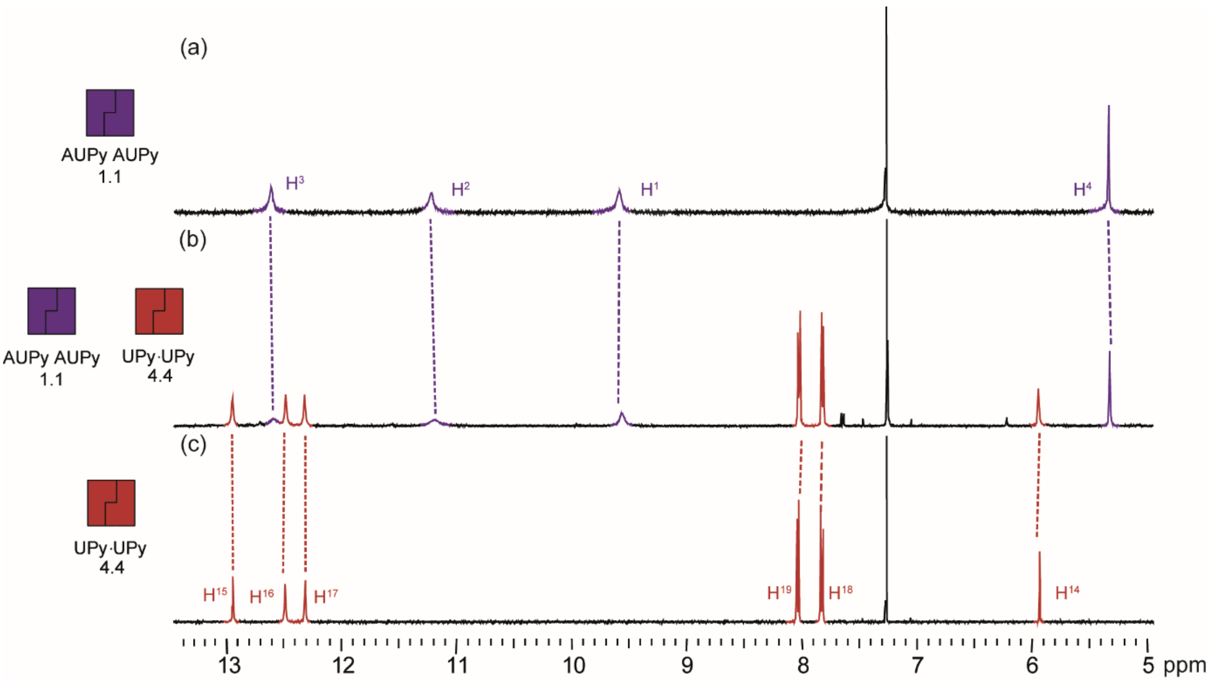

Figure S4. ^{1}H NMR (500 MHz, 10 mM, $CDCl_3$) for (a) AUPy **1·1** (b)1:1 AUPy **1·1** and UPy **4·4** (c) Upy **4·4**.

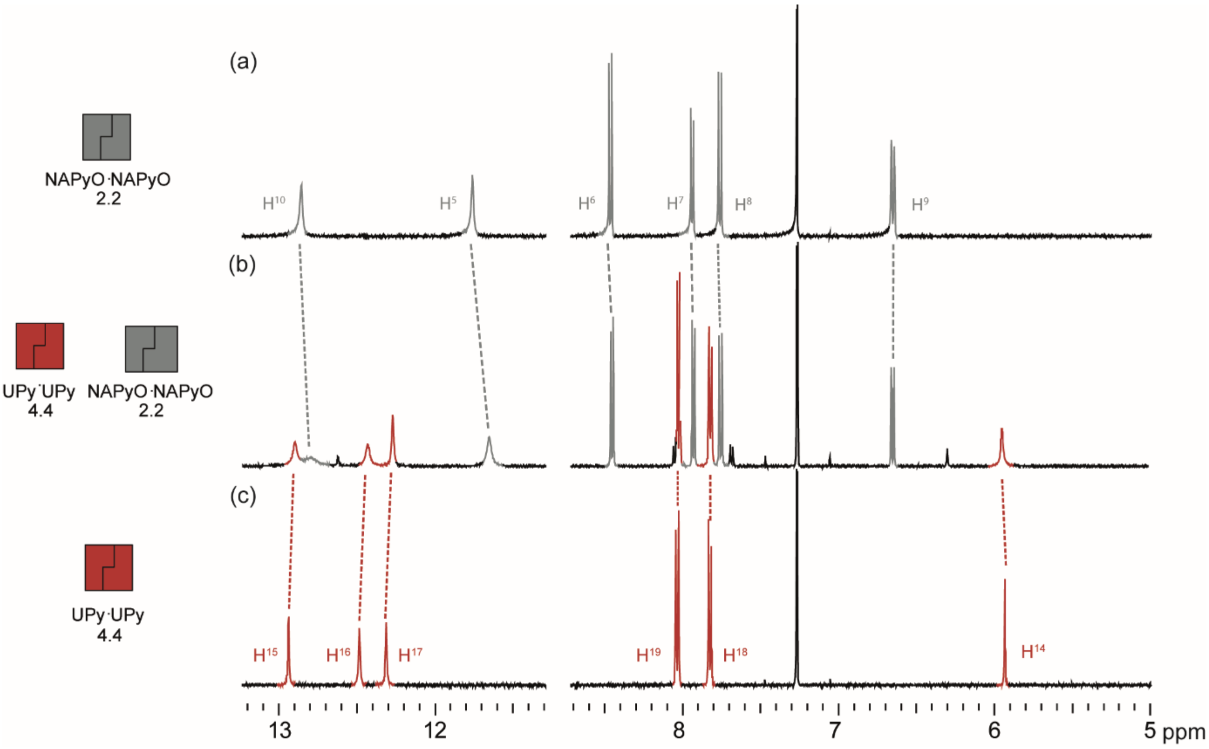

Figure S5. ^{1}H NMR (500 MHz, 10 mM, $CDCl_3$) for (a) NAPyO **1·1 (**b) 1:1 NAPyO·UPy **1·1** (c) UPy **1·1**.

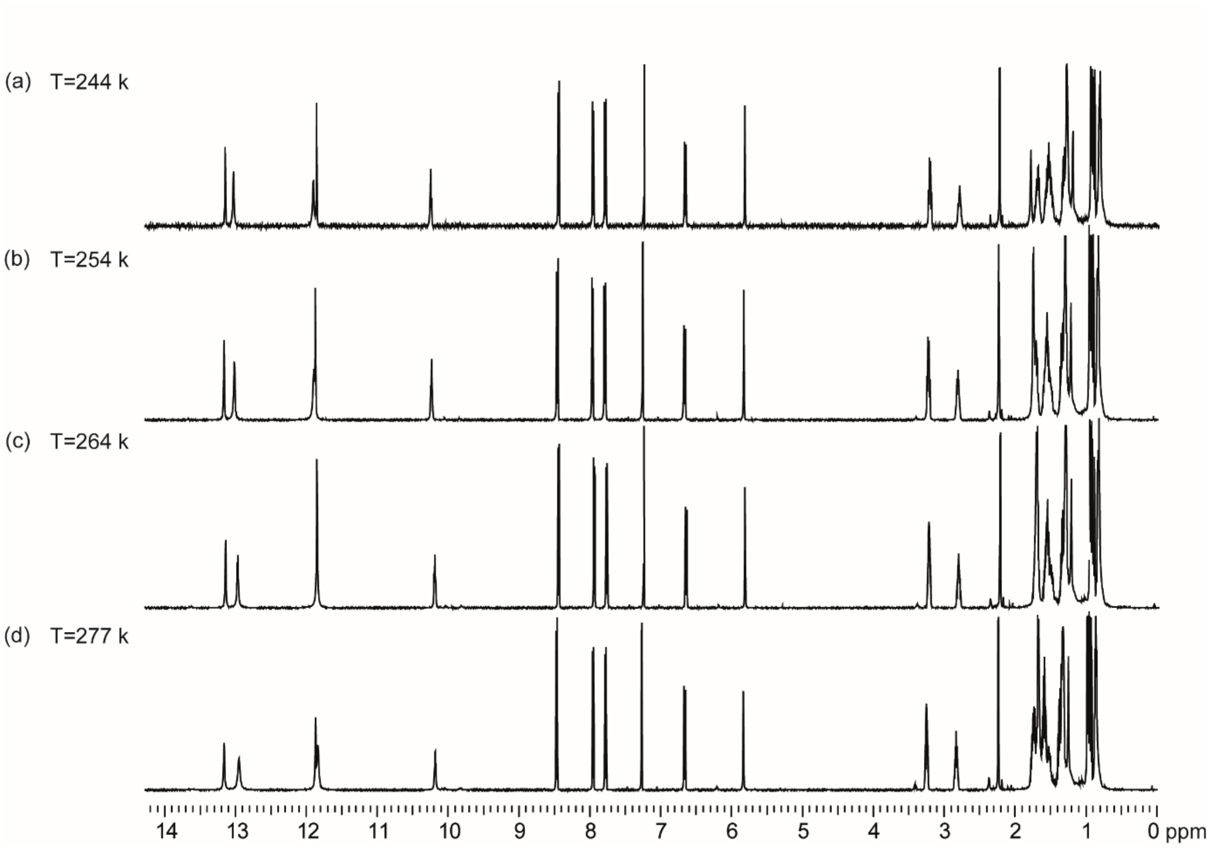

Figure S6. Variable temperature ^{1}H NMR (500 MHz, 10 mM, $CDCl_3$) of 1:1 NAPyO **1** and UPy **4** at (a) 244 K (b) 254 K (c) 264 K (d) 277 K.

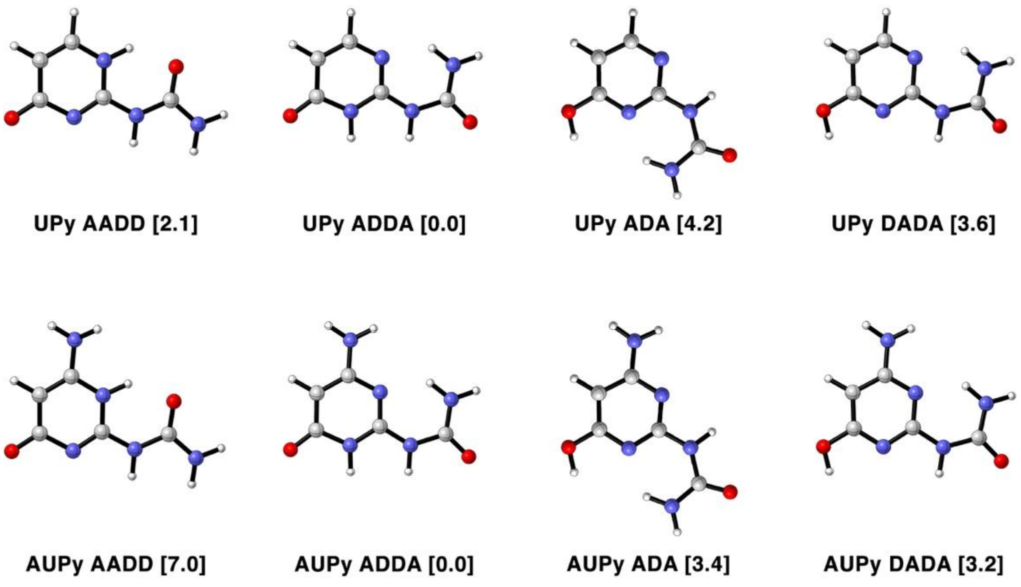

Figure S7. Optimized UPy and AUPy tautomers and their Gibbs free energies G between brackets [in kcal mol^{-1}] with respect to their most stable tautomer ADDA. Computed at the BLYP-D3(BJ)/TZ2P level of theory.

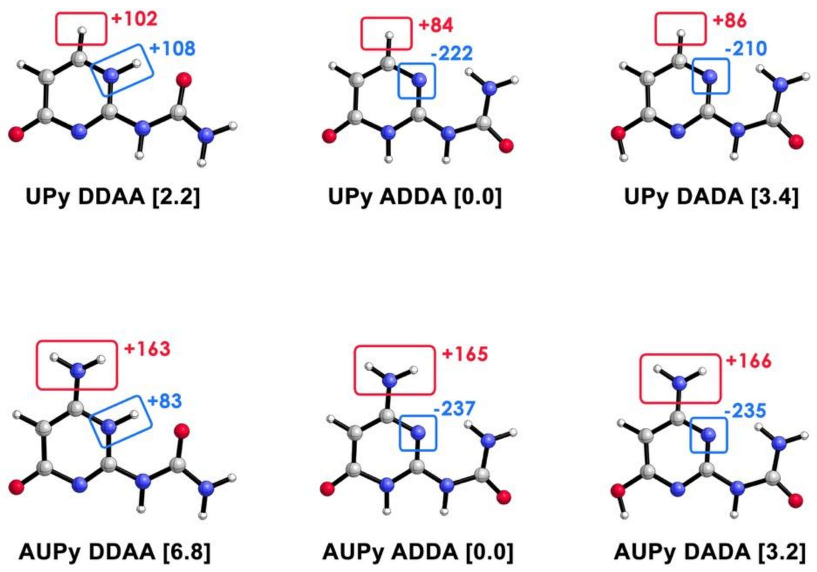

Figure S8. UPy and AUPy tautomers and their Gibbs free energies G between brackets [in kcal mol^{-1}] with respect to their most stable tautomer ADDA and the relevant Voronoi deformation density (VDD) charges [in me^{-}], computed at the BLYP-D3(BJ)/TZ2P level of theory. The R=NH_2 group is more electron donating than R=H. This is reflected by the VDD charges (red squares), which are more positive for AUPy than for UPy. This flow of electron density is favourable for the ADDA and DADA tautomers, because the hydrogen bond acceptor atoms N (blue squares) become more negatively charged. On the other hand, the same flow of electron density has a destabilizing effect in the DDAA conformations, because the hydrogen bond donating groups NH become less positively charged. Since these stabilizing (ADDA and DADA) and destabilizing (DDAA) effects are more pronounced when R=NH_2, the energetic differences between the AUPy tautomers are larger than the energetic differences of the AUPy tautomers. As a consequence, the tautomerization energy is larger for AUPy, which explains why AUPy forms DADA homodimers while UPy forms DDAA homodimers.

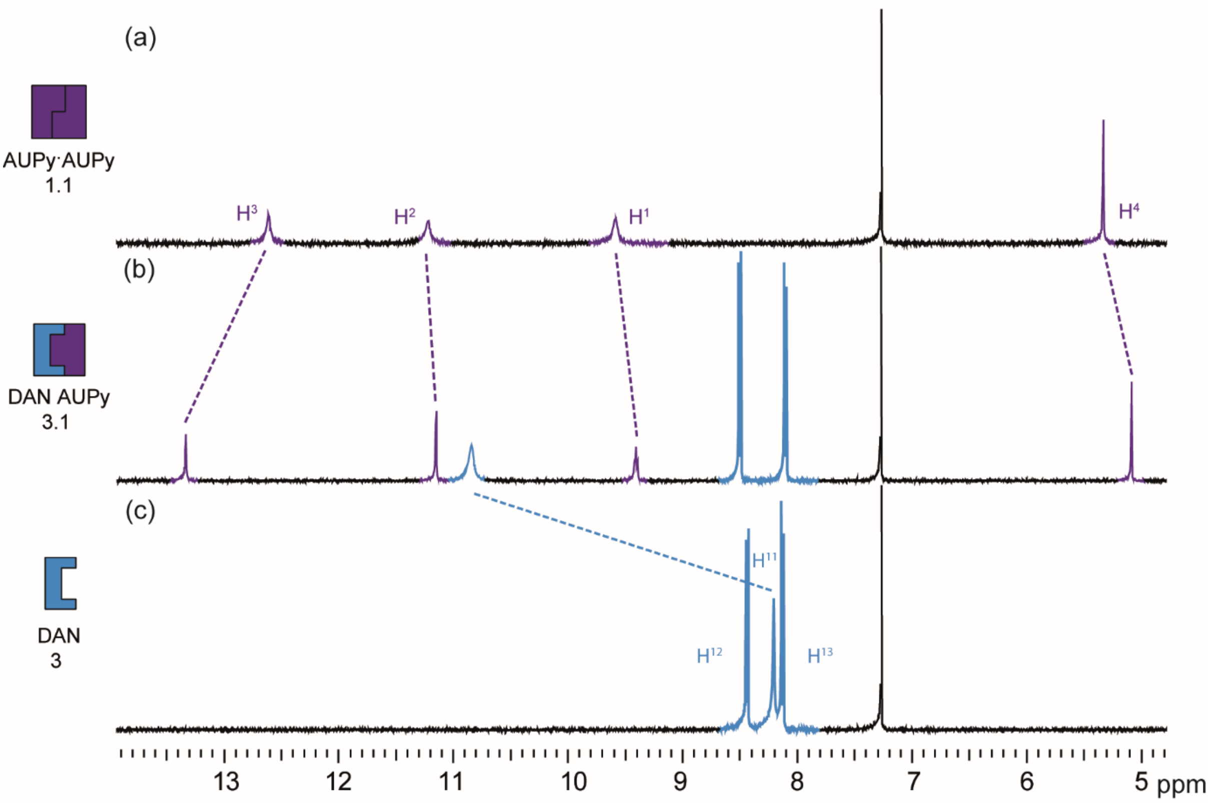

Figure S9. ^{1}H NMR (500 MHz, 10 mM, $CDCl_3$) for (a) AUPy **1·1** (b) 1:1 AUPy·DAN **1·3** (c) DAN **3**.

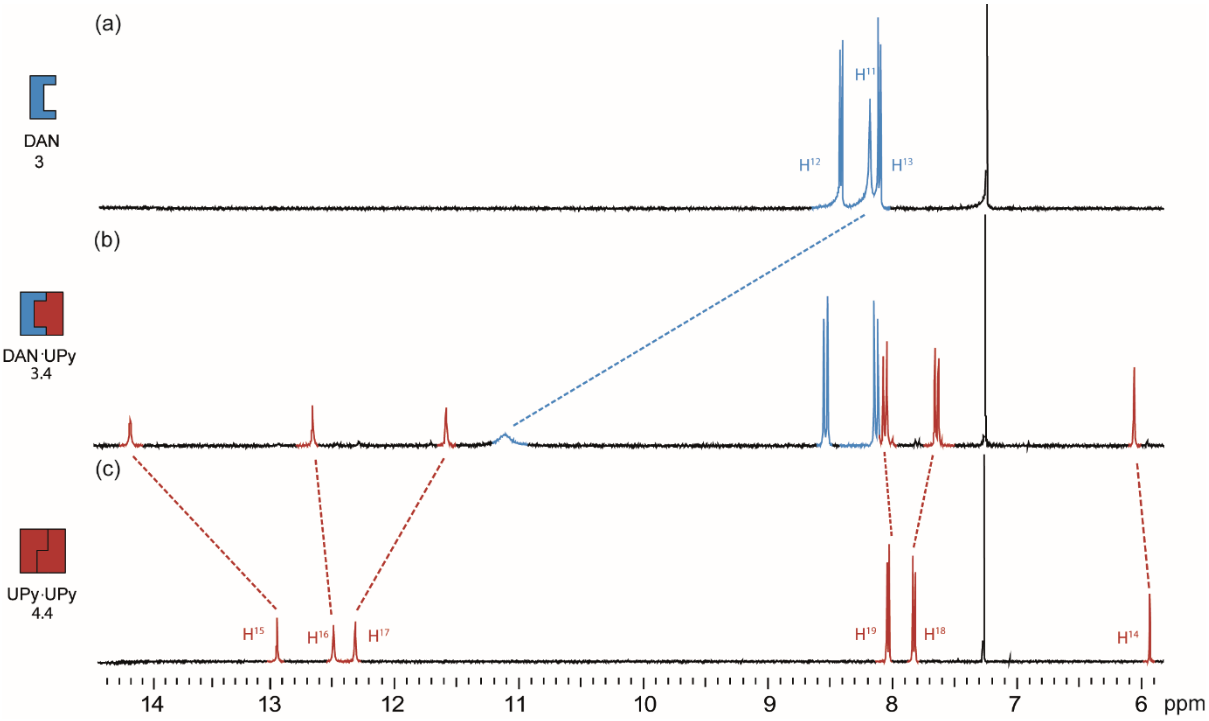

Figure S10. ^{1}H NMR (500 MHz, 10 mM, $CDCl_3$) for (a) DAN **3** (b) 1:1 DAN·UPy **3·4** c) UPy **4·4**.

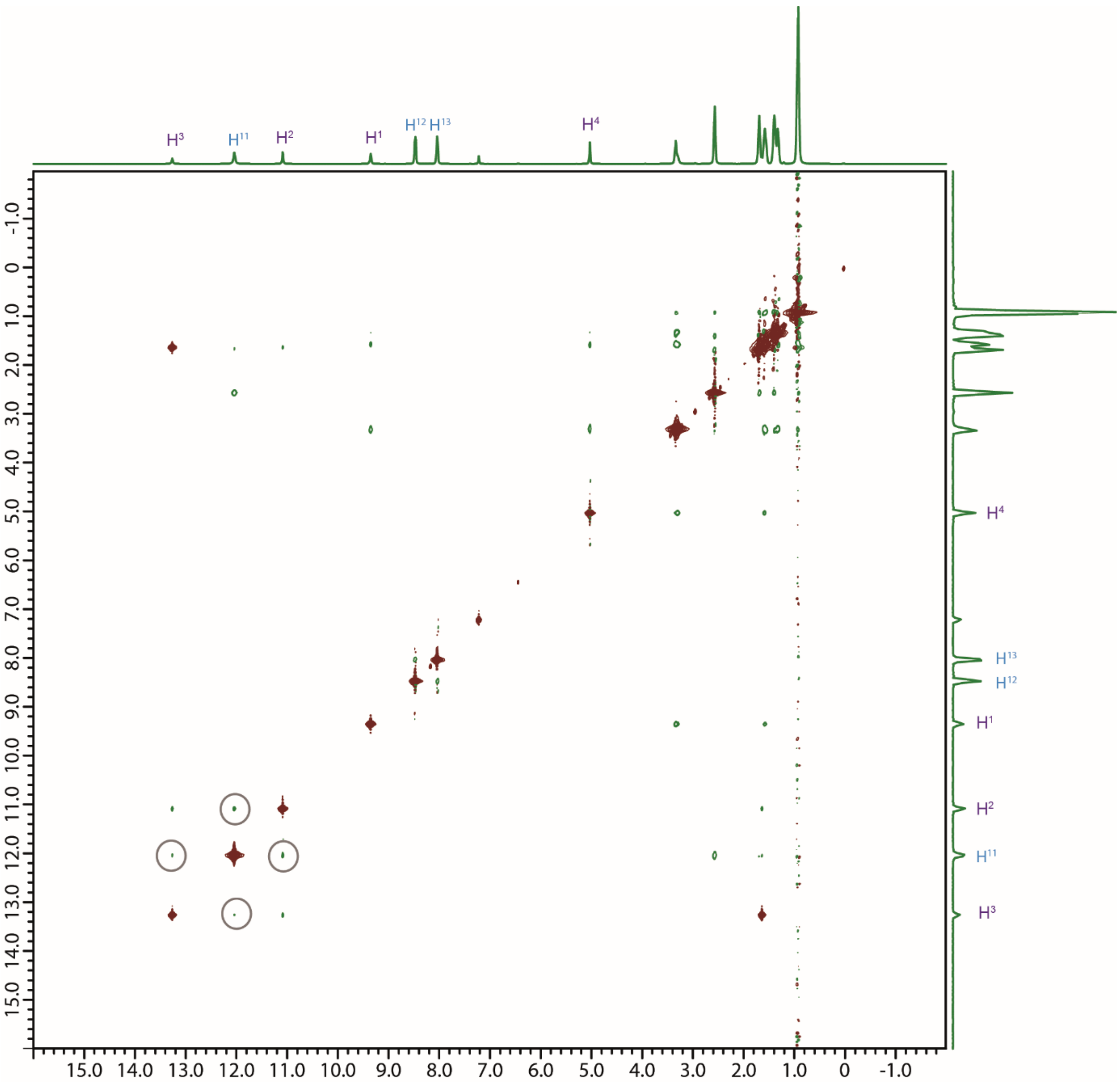

Figure S11. 2D ^1H-^1H NOESY NMR (600 MHz, 50 mM, $CDCl_3$, 298 K) for 1:1 AUPy·DAN **1·3.** Grey circles highlight the NOE cross peaks between NH signals fo AUPy and DAN (H^3-H^{11} and H^2-H^{11})**.**

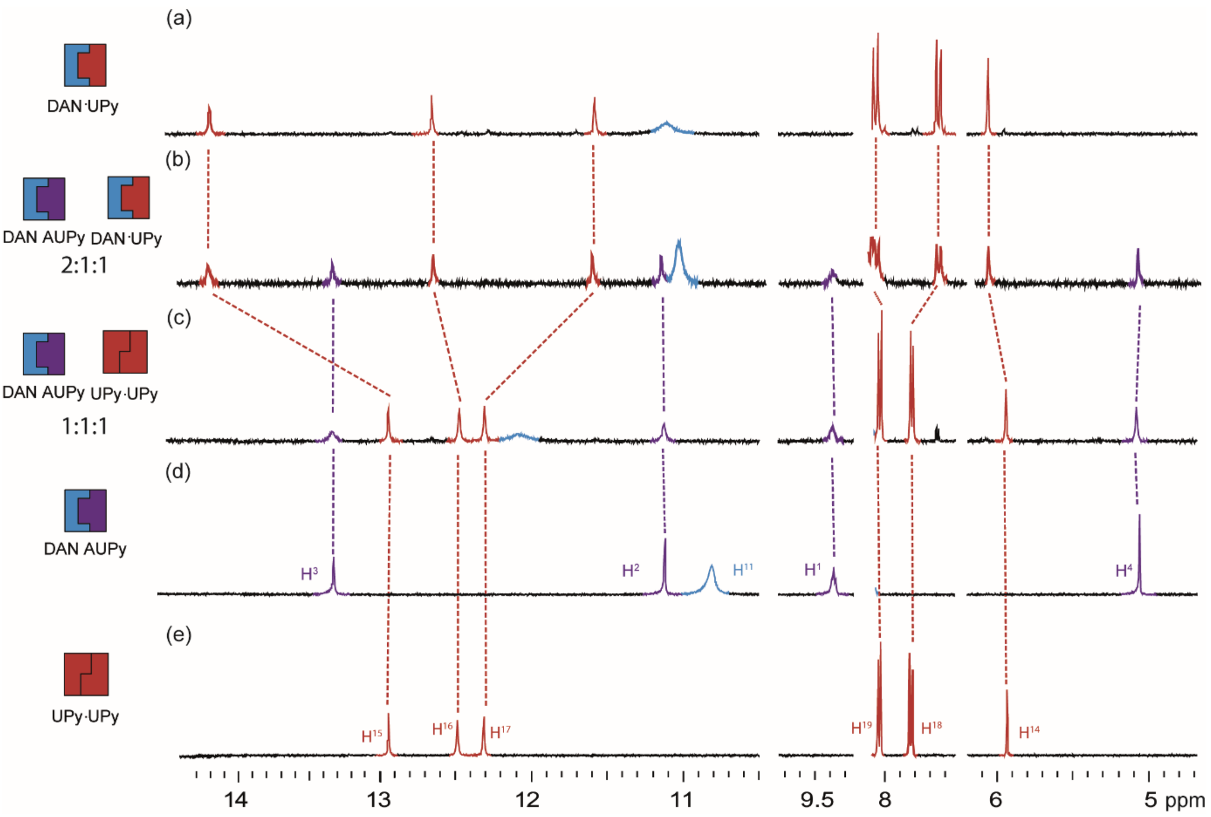

Figure S12. ^{1}H NMR (500 MHz, 10 mM, $CDCl_3$) for (a) 1:1 AUPy·DAN **1·3** (b) 2:1:1 AUPy **1**, DAN **3** and UPy **4** (c) 1:1:1 AUPy **1**, DAN **3** and UPy **4** (d)1:1 AUPy·DAN **1·3** (e) UPy **4·4**.

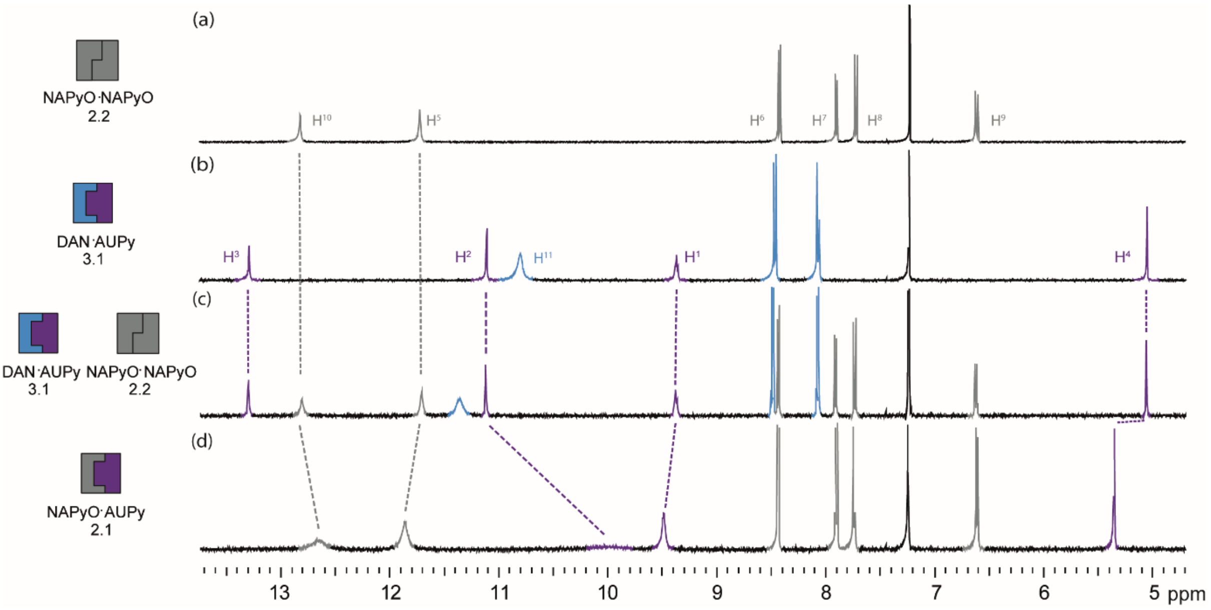

Figure S13. ^{1}H NMR (500 MHz, 10 mM, $CDCl_3$) for (a) NAPyO **2·2** (b)1:1 AUPy·DAN **1·3** (c)1:1:1 AUPy **1**, NAPyO **2** and DAN **3**, (d) 1:1 AUPy·NAPyO **1·2**

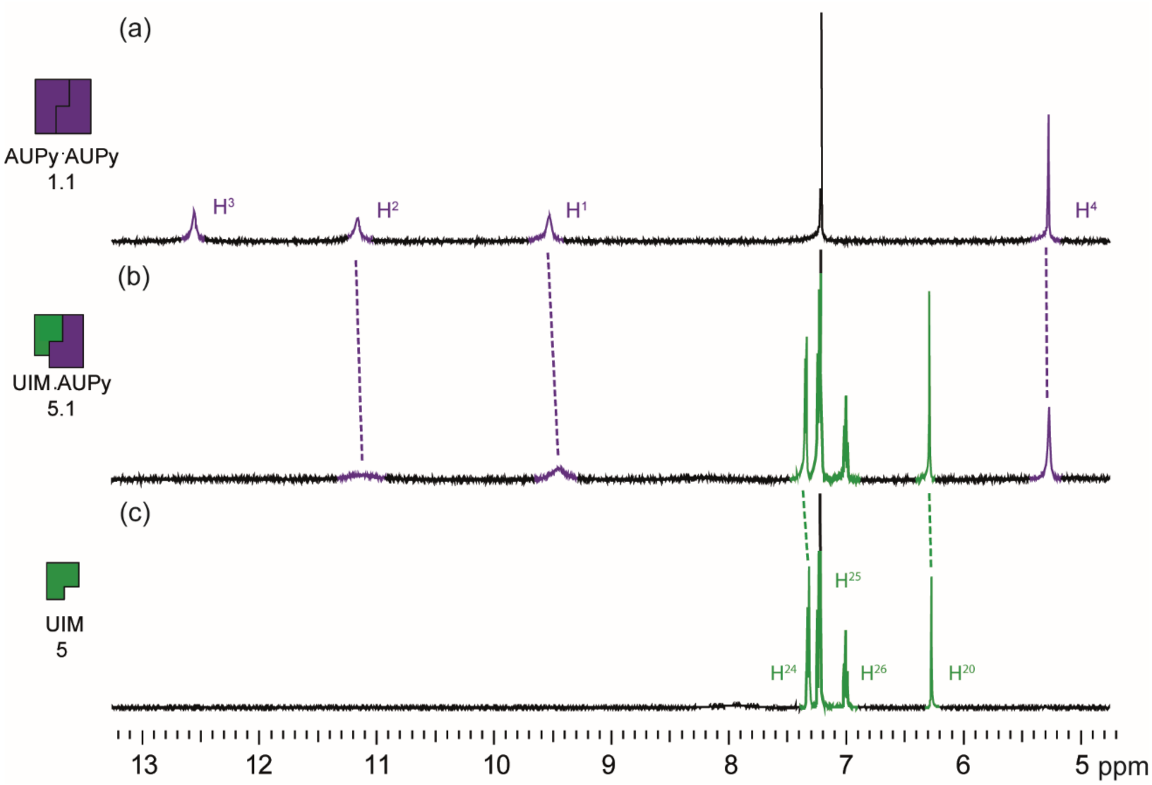

Figure S14. ^{1}H NMR (500 MHz, 10 mM, $CDCl_3$) for (a) AUPy **1·1** (b) 1:1 AUPy·UIM **1·5** (c) UIM **5**.

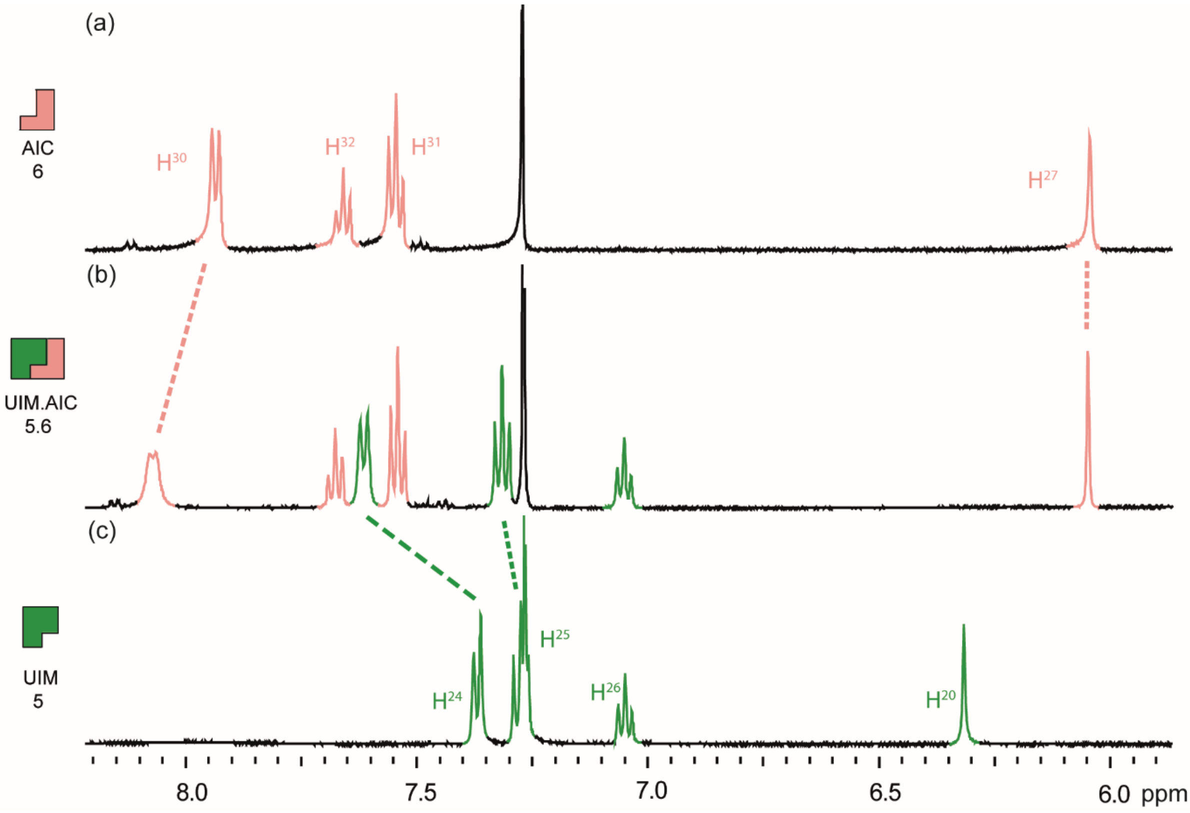

Figure S15. ^{1}H NMR (500 MHz, 10 mM, $CDCl_3$) for (a) AIC **6** (b) 1:1 UIM·AIC **5·6** (c) UIM **5**.

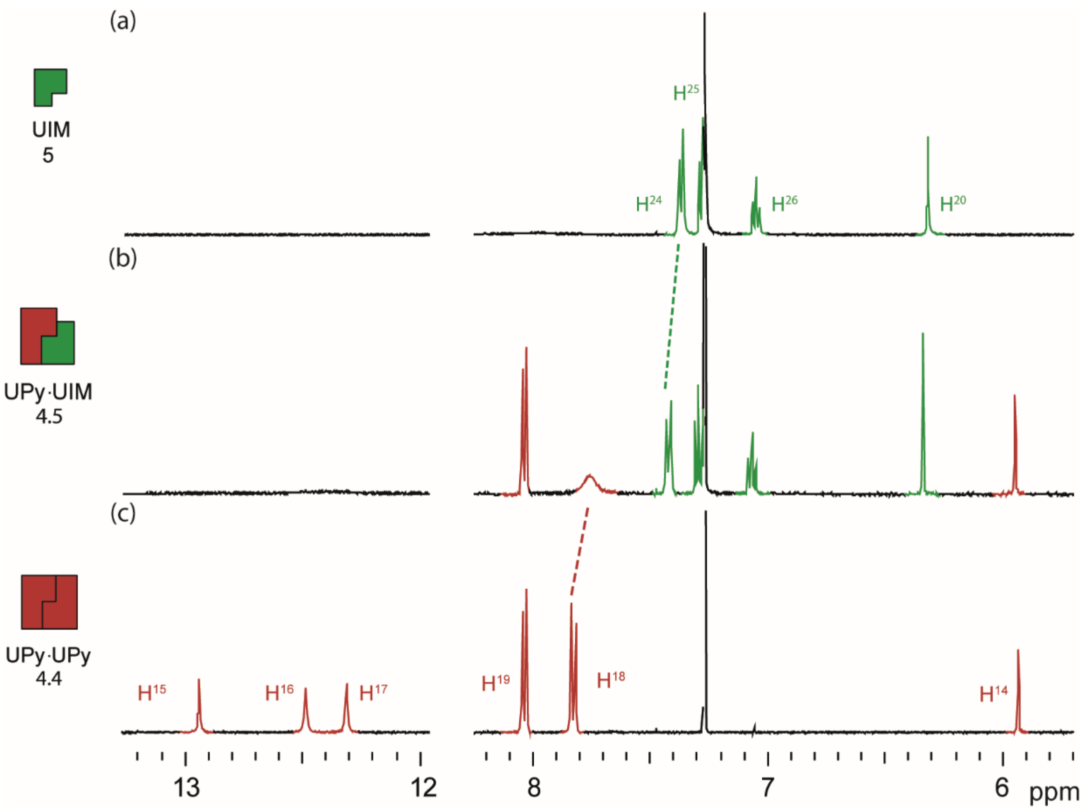

Figure S16. ^{1}H NMR (500 MHz, 10 mM, $CDCl_3$) for (a) UIM **5** (b) 1:1 UPy·UIM **4·5** (c) UPy **4·4**.

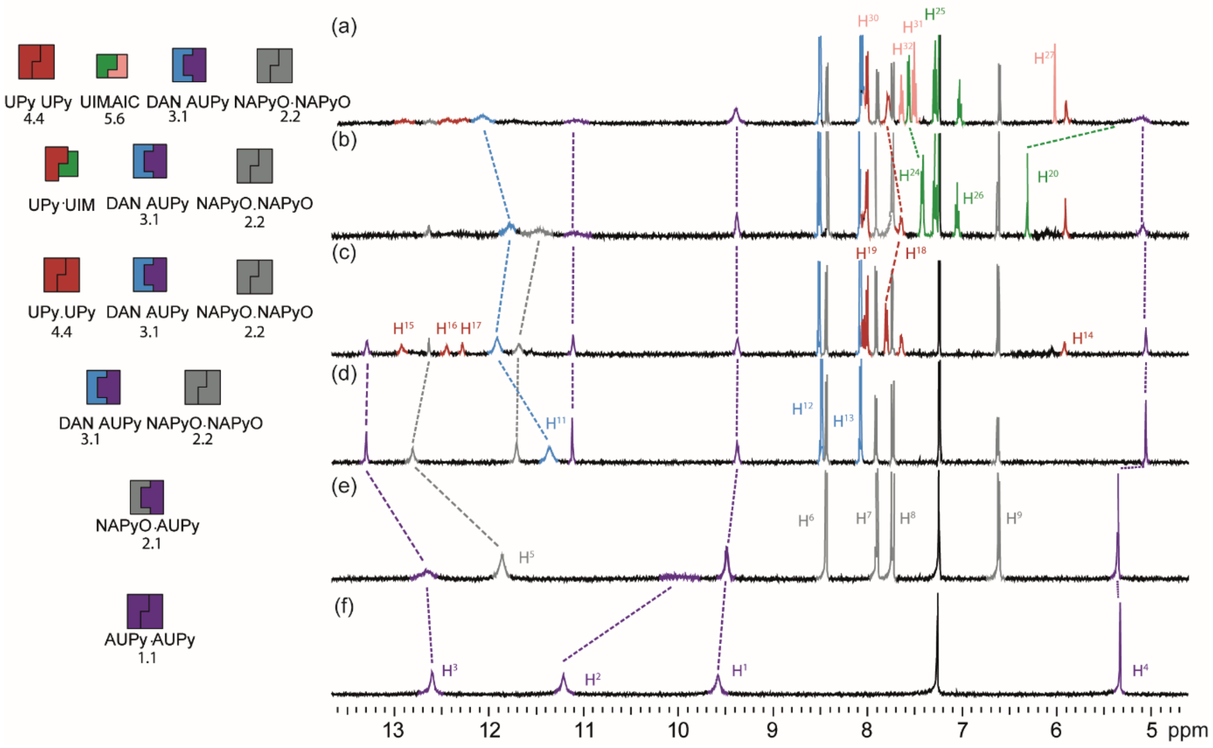

Figure S17. Pathway A ^{1}H NMR (10 mM, $CDCl_3$) six component signalling cascade a) UPY, UIM, AIC, DAN, AUPy and NAPyO b) UPY, UIM, DAN, NAPyO and AUPy c)UPY, DAN, AUPy AND NAPyO d)AUPy, DAN and NAPyO e)AUPy and NAPyO f)AUPy.

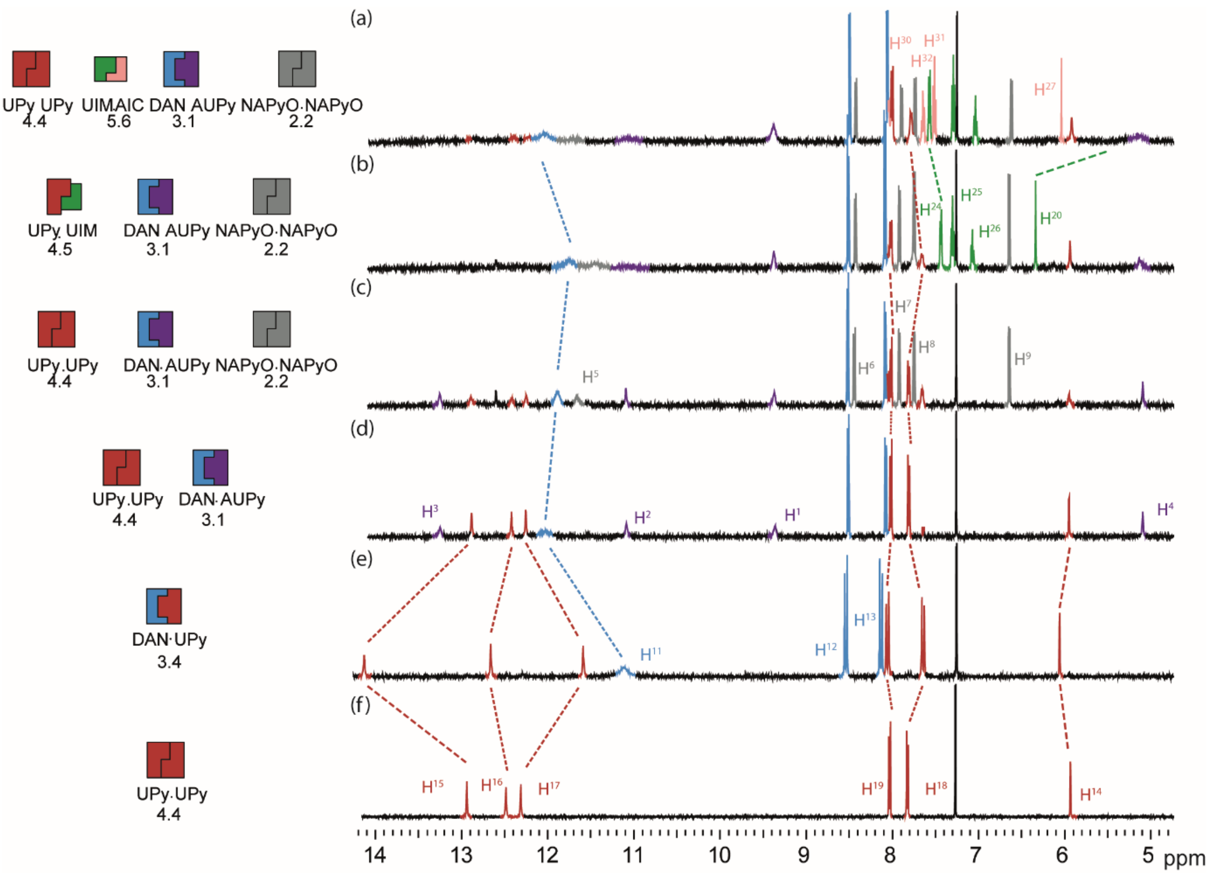

Figure S18. Pathway B ^{1}H NMR (500 MHz, 10 mM, $CDCl_3$) six component signalling cascade (a) AUPy **1**, NAPyO **2**, DAN **3**, UPy **4**, UIM **5** and AIC **6**, (b) AUPy **1**, NAPyO **2**, DAN **3**, UPy **4** and UIM **5** (c) AUPy **1**, NAPyO **2**, DAN **3**, UPy **4** (d) AUPy **1**, DAN **3** and UPy **4**, (e) DAN **3** and UPy **4** (f) UPy **4**.

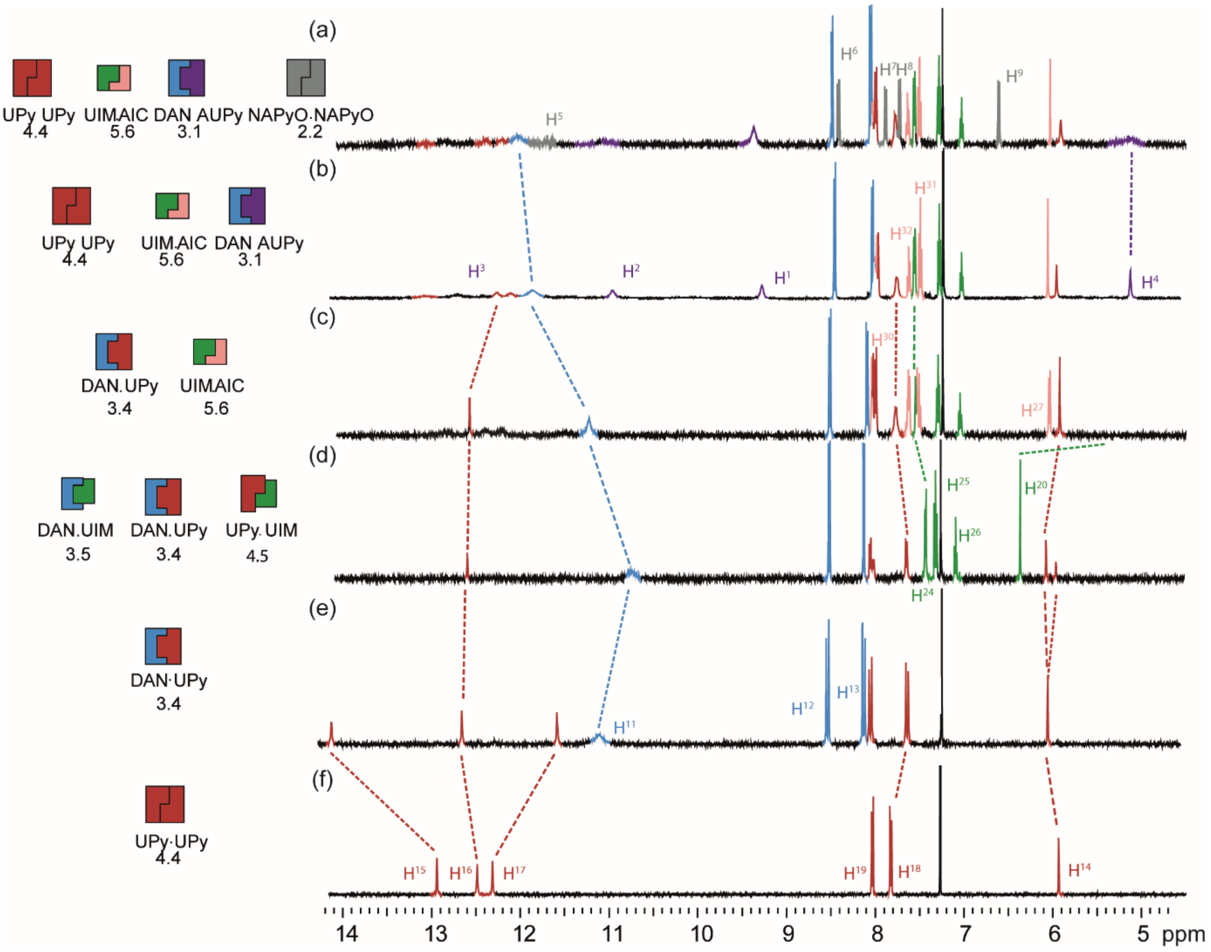

Figure S19.Pathway C ^{1}H NMR (500 MHz, 10 mM, $CDCl_3$) six component signalling cascade a) AUPy **1**, NAPyO **2**, DAN **3**, UPy **4**, UIM **5**, and AIC **6**, b) AUPy **1**, DAN **3**, UPy **4**, UIM **5**, and AIC **6** (c) DAN **3**, UPy **4**, UIM **5**, and AIC **6** (d) DAN **3**, UPy **4**, and UIM **5** (e) DAN **3** and UPy **4** (f) UPy **4.**

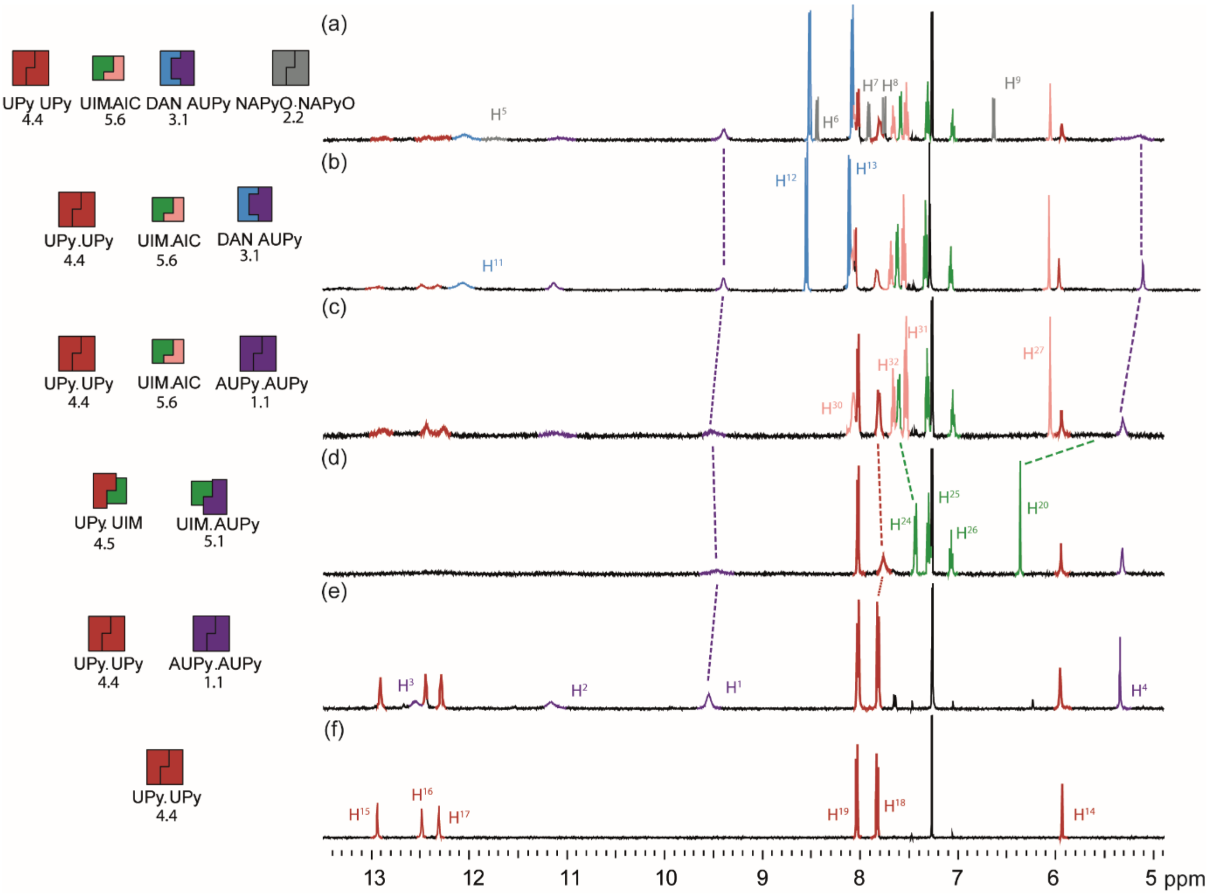

Figure S20. Pathway D ^{1}H NMR (500 MHz, 10 mM, $CDCl_3$) six component signalling cascade (a) AUPy **1**, NAPyO **2**, DAN **3**, UPy **4**, UIM **5**, and AIC **6**, (b) AUPy **1**, DAN **3**, UPy **4**, UIM **5**, and AIC **6**, (c) AUPy **1**, UPy **4**, UIM **5**, and AIC **6**, (d) AUPy **1**, UPy **4**, and UIM **5**, (e) AUPy **1**, and UPy **4**, (f) UPy **4**.

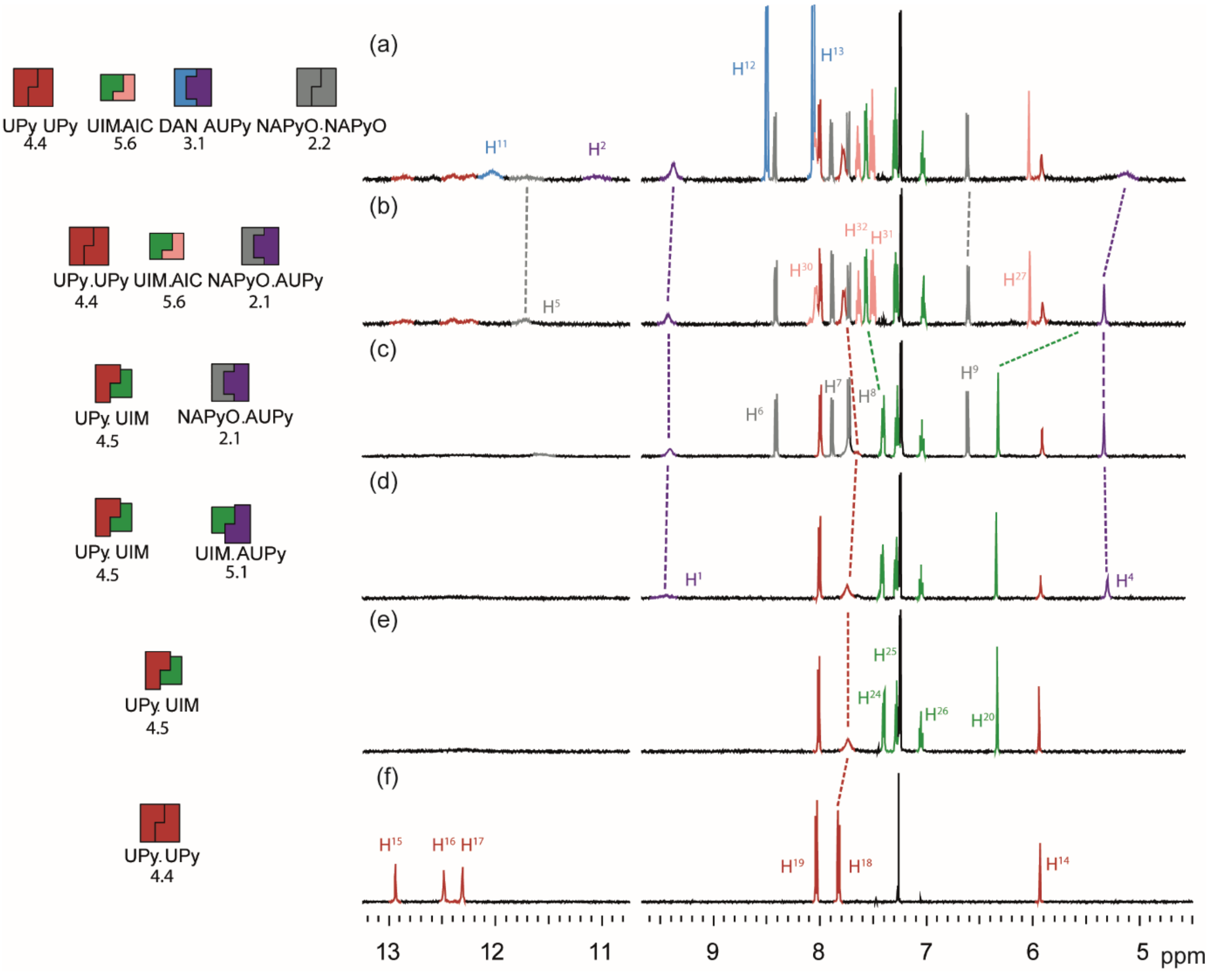

Figure S21. Pathway F ^{1}H NMR 500 MHz, 10 mM, $CDCl_3$) six component signalling cascade (a) AUPy **1**, NAPyO **2**, DAN **3**, UPy **4**, UIM **5**, and AIC **6**, (b) AUPy **1**, NAPyO **2**, UPy **4**, UIM **5**, and AIC **6**, (c) AUPy **1**, NAPyO **2**, UPy **4**, and UIM **5**, (d) AUPy **1**, UPy **4**, and UIM **5**, (e) UPy **4**, and UIM **5**, (f) UPy **4**.

2. Synthetic Procedures

Compounds **3 (DAN)[1] and 4 (UPy)[2]** were synthesized as described previously Compounds **1 (AUPy), 2 (NAPyO), 5 (UIM), 6 (AIC) 7 (NAPyO isopropyl analogue) and 8 (DAN isopropyl analogue),** were synthesized as described below:

2-amino-6-(dibutylamino) 4-pyrimidinol

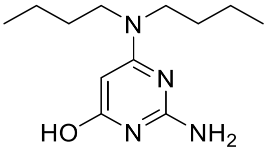

2-Amino-6-chloro-4-pyrimidinol (10.01 g, 68.70 mmol) was dissolved in ethyleneglycol (170 mL) and dibutylamine (29 mL, 171.80 mmol) was added. The solution was heated for 5 hours at 135 °C after which it was allowed to cool to room temperature. The solution was poured into saturated aqueous NH_4Cl solution (500 mL) and extracted three times with ethyl acetate. The combined organic layers were evaporated *in vacuo* to a volume of 100 mL and extracted with a saturated aqueous $NaHCO_3$ solution (300 mL) and a saturated aqueous NaCl solution (300 mL). The organic layer was dried, filtered and evaporated *in vacuo*. The yellow solid was recrystallized with acetonitrile twice affording 2-amino-6-(dibutylamino) 4-pyrimidinol (10.44 g, 64%) as a colourless solid. ^{1}H-NMR (500 MHz, $CDCl_3$) δ: 12.39 (s, 1H, O*H*), 5.02 (s, 2H, N*H₂*), 4.81 (s, 1H, Ar-*H*), 3.29 (bs, 4H, N-C*H₂*), 1.54-1.49 (m, 4H, NCH_2-C*H₂*), 1.28 (sext, *J* 7.2, 4H, NCH_2CH_2-C*H₂*), 0.91 (t, *J* 7.2, 6H, C*H₃*); ^{13}C-NMR (125 MHz, $CDCl_3$) δ: 165.8, 163.6, 154.4, 76.3, 48.1, 29.6, 20.1, 13.9; $ν_{max}$: 3317, 3166, 2956, 2930, 2871, 2730, 1586, 1558, 1490, 1453 cm^{-1}; MS (ESI+) m/z $[M+H]^+$ calculated for $C_{12}H_{22}N_4O$: 239.18., found: $[M+H]^+$ 239.17.

1-butyl-3-(4-(dibutylamino)-6-hydroxypyrimidin-2-yl)urea

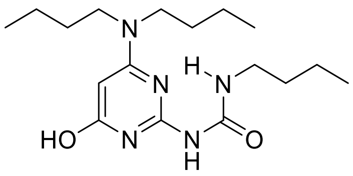

A suspension of 2-amino-6-(dibutylamino) 4-pyrimidinol (5.00 g, 21.07 mmol), butyl isocyanate (3.56 mL, 31.60 mmol) and 4-*N*,*N*-dimethylaminopyridine (6.44 g, 52.70 mmol) in anhydrous chloroform (150 mL) was heated under nitrogen at 70 °C overnight. After cooling the solution was concentrated. Dissolved in dichloromethane and filtered through silica to remove excess 4-*N*,*N*-dimethylaminopyridine. Ether (20 mL) was added and the white precipitate formed was further purified by recrystallization (acetone) affording 1-(4-(dibutylamino)-6-hydroxypyrimidin-2-yl)-3-dodecylurea (0.68 g, 10%) as a colourless solid. ^{1}H-NMR (500 MHz, $CDCl_3$) δ: 12.57 (bs, 1H, O*H*), 11.18 (bs, 1H, N*H*), 9.54 (bs, 1H, N*H*), 5.31 (s, 1H, Ar-*H*), 3.40-3.20 (m, 6H, N-C*H₂*), 1.63-1.47 (m, 8H, NCH_2-C*H₂*), 1.43-1.27 (m, 4H, NCH_2CH_2-C*H₂*), 0.98-0.88 (m, 9H, C*H₃*) ppm; ^{13}C-NMR (125 MHz, $CDCl_3$) δ: 170.9, 162.5, 157.4, 157.0, 78.7, 50.0, 40.0, 31.9, 30.3, 29.6, 29.4, 14.0 ppm; $ν_{max}$ (solid state) = 3218, 3127,

3019, 2955, 2922 , 2852, 2548, 1674, 1613, 1559, 1524, 1504, 1454 cm^{-1}; MS (ESI+) m/z $[M+H]^+$ calculated for $C_{17}H_{31}N_5O_2$: 338.2556., found: $[M+H]^+$ 338.2566.

7-Amino-1,8-naphthridin-2-ol

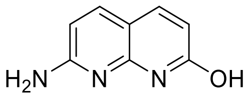

Concentrated sulfuric acid (40 mL), was added drop wise at 0 °C to a ground mixture of 2,6-diaminopyridine (6.67 g, 61.1 mmol) and D-malic acid (9.07 g, 67.6 mmol). The reaction mixture was then heated to 110 °C for 4 hours before being cooled back to 0 °C. Saturated aqueous NH_4OH solution was then added drop wise to pH 9, before the reaction mixture was filtered and subsequently washed with water and diethyl ether, to give 7-amino-1,8-naphthridin-2-ol (5.31 g, 54 %) as an olive green solid. ^{1}H-NMR (500 MHz, DMSO-*d*6) δ: 7.67 (2H, m, Ar-*H*), 6.99 (2H, s, NH_2), 6.37 (1H, d, *J* 8.5 Hz, Ar-*H*), 6.14 (1H, d, *J* 9.2 Hz, Ar-*H*) ppm; ^{13}C-NMR (125 MHz, DMSO-d_6) δ: 164.1, 160.9, 150.7, 140.1, 137.7, 115.2, 105.6, 105.4 ppm; v_{max}: 3500-2500 (broad), 1617, 1513 cm^{-1}; MS (ESI+) *m/z* $[M+H]^+$ calculated for $C_8H_8N_3O$: 162.0667, found: $[M+H]^+$ 161.9396.

2-ethyl-*N*-(5methyl-1,5-naphtyridine)hexanamide

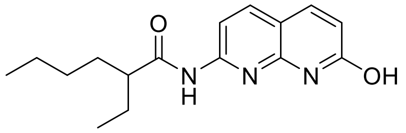

2-Ethylhexanoyl chloride (1.2 mL, 7.14 mmol) was slowly added to a solution of 7-amino-4-methyl-1,8-naphthyridin-2(1H)-one (1.00 g, 5.71 mmol) in dry pyridine (10.0 mL). The mixture was heated to 110 ^{o}C for 24 h and the solvent removed in vacuo. The residue was diluted with dichloromethane (100 mL). The organic phase was washed with 0.1 M aqueous HCl solution (10 mL), water (10 mL), saturated NaHCO3 solution (10 mL) and dried. After evaporation of the solvent the crude product was purified by re-crystallization from acetone yielding 2-ethyl-N-(5methyl-1,5-naphtyridine)hexanamide (1.07 g, 65%) as a colourless powder. ^{1}H-NMR (500 MHz, $CDCl_3$) δ: 12.83 (s, 1H; N*H*), 11.73 (s, 1H; N*H*), 8.45 (d, *J* 8.6 Hz, 1H, Ar-*H*), 7.91 (d, *J* 8.6 Hz, 1H; Ar-*H*), 7.73 (d, *J* 9.4 Hz, 1H, Ar-*H*), 6.62 (dd, J_1 9.4 Hz, J_2 1.9 Hz, 1H, Ar-*H*), 2.92-2.77 (m, 1H; C*H*), 1.81-1.69 (m, 2H; CH_2), 1.54-1.45 (m, 4H; CH_2), 1.40-1.25 (m, 4H; CH_2), 0.96 (t, *J* 7.2 Hz. 3H; CH_3), 0.85 (t, *J* 7.2 Hz. 3H; CH_3) ppm; ^{13}C-NMR (125 MHz, $CDCl_3$) δ: 177.8, 154.0, 148.5, 148.4, 136.0, 119.0, 112.0, 110.6, 48.4, 32.5, 29.7, 26.2, 22.9, 18.6, 14.0, 11.9 ppm; v_{max} (solid state) = 3175, 3135, 3069, 2960, 2933, 1705, 1657, 1616, 1579, 1525 cm^{-1}; MS (ESI+) *m/z* $[M+H]^+$ calculated for $C_{16}H_{21}N_3O_2$: 288.38., found: $[M+H]^+$ 288.40

7-[(propan-2-yl)amino]-1,8-naphthyridin-2-ol

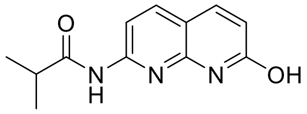

Isobutyryl chloride (134 mg, 1.26 mmol) was slowly added to a solution of 7-amino-4-methyl-1,8-naphthyridin-2(1H)-one (100 mg, 0.63 mmol) in dry pyridine (5.0 mL). The mixture was heated to 110 ^{o}C for 1 h. Precipitate was filtered, washed with methanol and recrystallized from acetonitrile to give colourless crystals of 7-[(propan-2-yl)amino]-1,8-naphthyridin-2-ol (95 mg, 66%). ^{1}H-NMR (500 MHz, DMSO-*d*$_6$) δ: 11.82 (s, 1H; N*H*), 10.46 (s, 1H; N*H*), 8.04 (d, *J* 8.6 Hz, 1H, Ar-*H*), 7.95 (d, *J* 8.6 Hz, 1H; Ar-*H*), 7.84 (d, *J* 9.4 Hz, 1H, Ar-*H*), 6.42 (d, *J* 9.4 Hz, 1H, Ar-*H*), 2.86 (quinet, *J* 6.8 Hz, 1H, C*H*), 1.09 (s, 3H, C*H*$_3$), 1.08 (s, 3H, C*H*$_3$) ppm; ^{13}C-NMR (125 MHz, DMSO-*d*$_6$) δ: 177.7, 155.8, 154.7, 153.2, 140.9, 140.1, 121.9, 119.4, 115.7, 35.2, 19.7 ppm; ν_{max} (solid state) = 3044, 2972, 2925, 2868, 1700, 1624, 1525 cm^{-1}; MS (ESI+) *m/z* [M+H]$^+$ calculated for $C_{12}H_{14}N_3O_2$: 232.1081, found: [M+H]$^+$ 232.1080

***N*2,*N*7-bis(propan-2-yl)-1,8-naphthyridine-2,7-diamine**

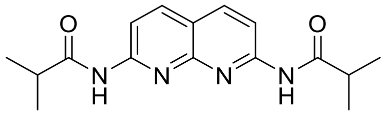

7-[(propan-2-yl)amino]-1,8-naphthyridin-2-chloride (75 mg, 0.30 mmol), isobutyramide (31 mg, 0.36 mmol), potassium carbonate (58 mg, 0.42 mmol), palladium(II) acetate (3 mg, 0.015 mmol) and xantphos (17 mg, 0.03 mmol) were suspended in 1,4-dioxane (5 mL) in a schlenck tube. The reaction mixture was heated to 100 °C for 23 h before being cooled to room temperature and filtered through celite. The solvent was evaporated in vacuo and the crude product was purified by crystallisation (MeOH) to give *N*2,*N*7-bis(propan-2-yl)-1,8-naphthyridine-2,7-diamine (32 mg, 34%) as colourless needles; ^{1}H-NMR (400 MHz, $CDCl_3$) δ: 8.42 (d, *J* 8.6 Hz, 1H, Ar-*H*), 8.21 (s, 1H, N*H*), 8.10 (d, *J* 8.6 Hz, 1H, Ar-*H*), 2.60 (m, *J* 6.8 Hz, 1H, C*H*), 1.28 (s, 3H, C*H*$_3$), 1.26 (s, 3H, C*H*$_3$) ppm; ^{13}C-NMR (100 MHz, $CDCl_3$) δ: 176.0, 153.9, 153.7, 139.0, 121.9, 118.4, 113.4, 37.1, 19.3 ppm; ν_{max} (solid state) = 3284, 3138, 2967, 2933, 2872, 1690, 1499 cm^{-1}; MS (ESI+) *m/z* [M+H]$^+$ calculated for $C_{16}H_{21}N_4O_2$: 301.1620, found: [M+H]$^+$ 301.0924.

***N-tert*-Butoxycarbonylguanidine**

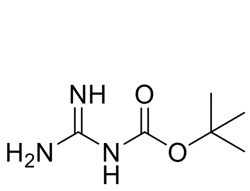

Guanidine hydrochloride (13.1 g, 138 mmol) was dissolved in deionised water (80 mL). Sodium hydroxide (11.0 g, 275 mmol) was added in portions with stirring, then di-*tert*-butyl dicarbonate (10.0 g, 46 mmol) in acetone (100 mL) was added at 0 °C and the reaction mixture was left to stir for 20 hrs,

once reaching room temperature. The volatiles were removed *in vacuo* to provide an aqueous suspension, which was extracted into ethyl acetate (2 x 150 mL). The organic layer was dried with sodium sulfate, filtered and concentrated under reduced pressure. The resultant solid was triturated (2:1 hexane:ethyl acetate) to provide N-tert-Butoxycarbonylguanidine (6.19 g, 85%) as a colourless solid; ^{1}H-NMR (500 MHz, DMSO-d_6): δ 6.81 (br s, 4 H,CNH_2 and 2x CN*H*), 1.36 (s, 9 H, CH_3 (^{t}Bu)) ppm; ^{13}C-NMR (125 MHz, DMSO-d_6) δ 163.4, 162.7, 75.5, 28.2 ppm; ν_{max} (solid state) = 3440, 3402, 3312, 1599, 1531 cm^{-1}; ESI-HRMS *m/z* found 182.0899 [M + Na]$^+$ $C_6H_{13}N_3NaO_2$ requires 182.0900.

Tert-Butyl 5-*tert*-1*H*-imidazol-2-yl-carbamate

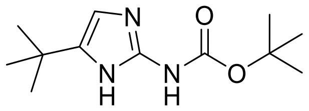

1-Bromopinacolone (1.70 mL, 12.6 mmol) was added to a solution of *N*-*tert*- butoxycarbonylguanidine (6.00 g, 37.6 mmol) in anhydrous dimethylformamide (40 mL). The reaction was stirred at room temperature for 72 hr. The solution was filtered and the precipitate was dried providing tert-Butyl 5-tert-1H-imidazol-2-yl-carbamate (708 mg, 23%) as a colourless powder. The filtrate was refrigerated to give a second crop (554 mg, 18%); ^{1}H-NMR (500 MHz, DMSO-d_6): δ 6.43 (s, 1 H, imidazole-*H*), 6.41 (br s, 2 H, 2xN*H*), 1.55 (s, 9 H, CH_3 (O^{t}Bu))), 1.14 (s, 9 H, CH_3 (C^{t}Bu)) ppm; ^{13}C-NMR (125 MHz, DMSO-d_6): δ 149.8, 149.0, 148.0, 102.9, 84.0, 31.2, 29.1, 27.5 ppm; ν_{max} (solid state) = 3433, 3279, 2971, 1724, 1640 cm^{-1}; ESI-HRMS *m/z* found 262.1525 [M + Na]$^+$ $C_{12}H_{21}N_3NaO_2$ requires 262.1526.

***4-tert*-Butyl-1*H*-imidazole-2-amine hydrochloride**

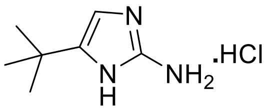

Tert-butyl 5-*tert*-1H-imidazol-2-yl-carbamate (750 mg, 3.10 mmol) was dissolved in 1M HCl in ethanol (30 mL) and refluxed for 16 hrs. The reaction was allowed to cool, concentrated and dried under pressure to give a colourless solid (400 mg, 2.28 mmol, 74%) ^{1}H-NMR (500 MHz, $CDCl_3$): δ 11.83 (s, 1H, N*H*), 11.23 (s, 1H, N*H*). 6.24 (s, 1 H, imidazole-*H*), 5.53 (br s, 2 H, NH_2), 1.25 (s, 9 H, CH_3 (C^{t}Bu)) ppm; ^{13}C-NMR (125 MHz, DMSO-d_6): δ 147.1, 135.8, 106.1, 29.8, 28.6 ppm; ν_{max} (solid state) = 3500-2800 (broad) cm^{-1}; ESI-HRMS *m/z* found 140.1190 [M + H]$^+$ $C_7H_{14}N_3$ requires140.1188.

1-(4-*tert*-Butyl-1*H*-imidazol-2-yl)-3-phenylurea

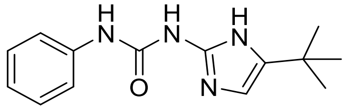

Tert-Butyl imidazole (3.5 g, 20 mmol) was dissolved in anhydrous chloroform (100 mL) and anhydrous *N*,*N*-diisopropylethylamine (1.78 mL, 9.99 mmol) under a nitrogen atmosphere. Reaction was cooled to 0 ^{o}C and phenylisocyanate (1.09 mL, 9.99 mmol) was added dropwise and stirred for 1 hour. The resulting solution was concentrated under reduced pressure and purified via column chromatography

(SiO_2, 9:1 dichloromethane:ethylacetate) and recrystallized from acetronitrile:methanol to give 1-(4-tert-Butyl-1H-imidazol-2-yl)-3-phenylurea (300 mg, 1.16 mmol, 12%).^{1}H-NMR (500 MHz, $CDCl_3$): δ 7.28 (d, *J* 8.3, 2H, Ar-*H*), 7.19 (t, *J* 8.0, 2H, Ar-*H*), 6.97 (t, *J* 7.4, 1H, Ar-*H*), 6.22 (s, 1 H, imidazole-*H*), 1.19 (s, 9 H, CH_3 (C^tBu)) ppm; ^{13}C-NMR (125 MHz, $CDCl_3$): δ 180.6, 154.7, 144.4, 138.3, 130.0, 123.3, 119.7, 101.6, 30.6, 29.7 ppm; ν_{max} (solid state) = 3184, 3069, 2961, 1549 cm^{-1}; ESI-HRMS *m/z* found 259.1561 $[M + H]^+$ $C_{14}H_{19}N_4O$ requires 259.1514.

Benzamido-5-methylisocytosine

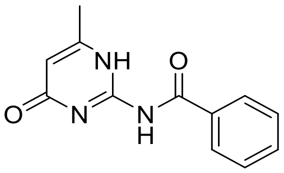

To a stirred mixture of methyl*iso*cytosine (3.00 g, 24.0 mmol) and 4-*N*,*N*-dimethylaminopyridine (150 mg, cat.) in anhydrous chloroform (120 mL) at room temperature under N_2 was added anhydrous *N*,*N*-diisopropylethylamine (6.30 ml, 36 mmol) and benzoyl chloride (3.00 mL, 26.4 mmol) and the reaction heated to reflux for 16 h. After cooling to room temperature the reaction mixture was washed with water (3 x 100 mL). The organic phase was dried over sodium sulfate, filtered and concentrated under reduced pressure. The product was purified by column chromatography (SiO_2, 1:9 ethylacetate:dichloromethane) to yield Benzamido-5-methylisocytosineas an off-white solid (4.10 g, 17.9 mmol, 75%); ^{1}H-NMR (500 MHz, $CDCl_3$) δ 8.11 (d, *J* 7.6, 2 H, Ar-*H*), 7.58 (t, *J* 7.6, 1 H, Ar-*H*), 7.44 (t, *J* 7.6, 2 H, Ar-*H*), 6.42 (s, 1 H, PyC*H*), 2.34 (s, 3 H, PyCH_3) ppm; ^{13}C NMR (125 MHz, DMSO-d_6) δ 171.6, 166.2, 163.7, 163.3, 134.1, 130.4, 128.7, 128.7, 102.0, 24.1 ppm; ν_{max} (solid state) = 3483, 3296, 3144, 1744 cm^{-1}; ESI-HRMS *m/z* found 230.0923 $[M + H]^+$ $C_{12}H_{12}N_3O_2$ requires 230.0924.

3. Spectral Data

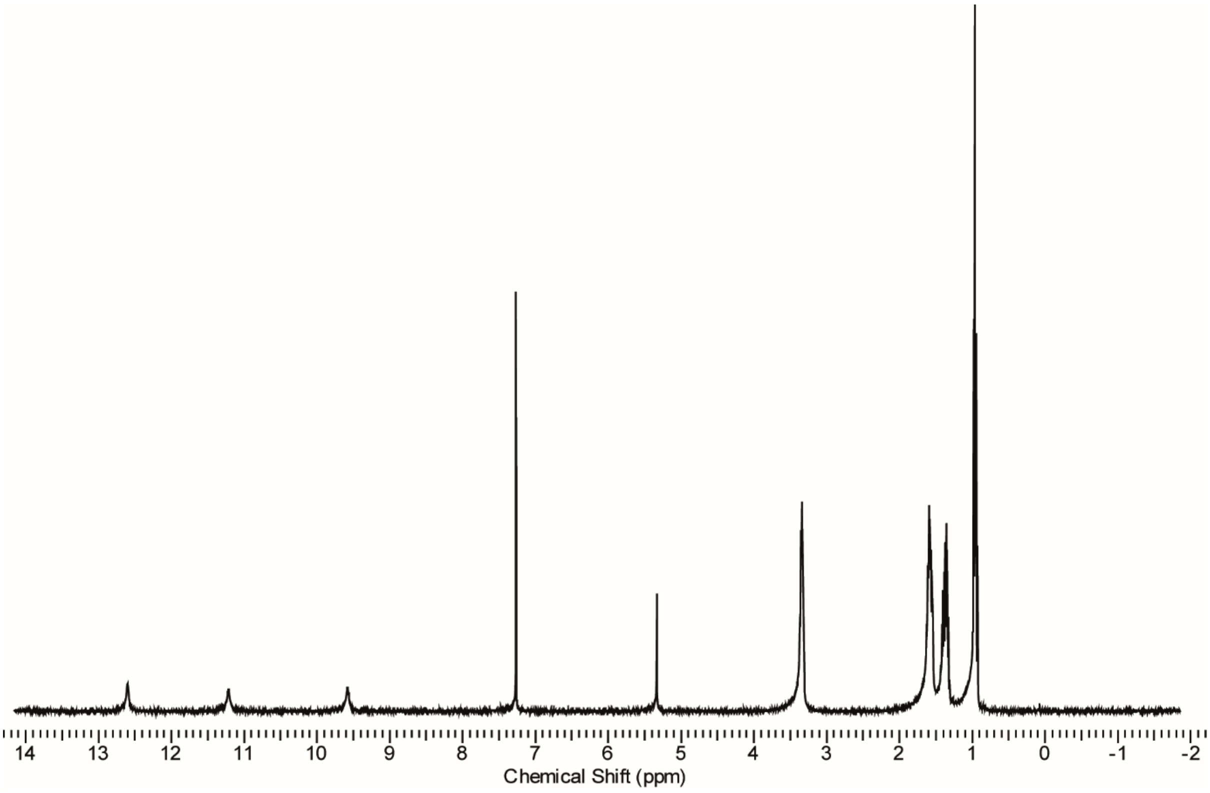

^{1}H NMR AUPy **1** (*500 MHz,* 10 mM, $CDCl_3$)

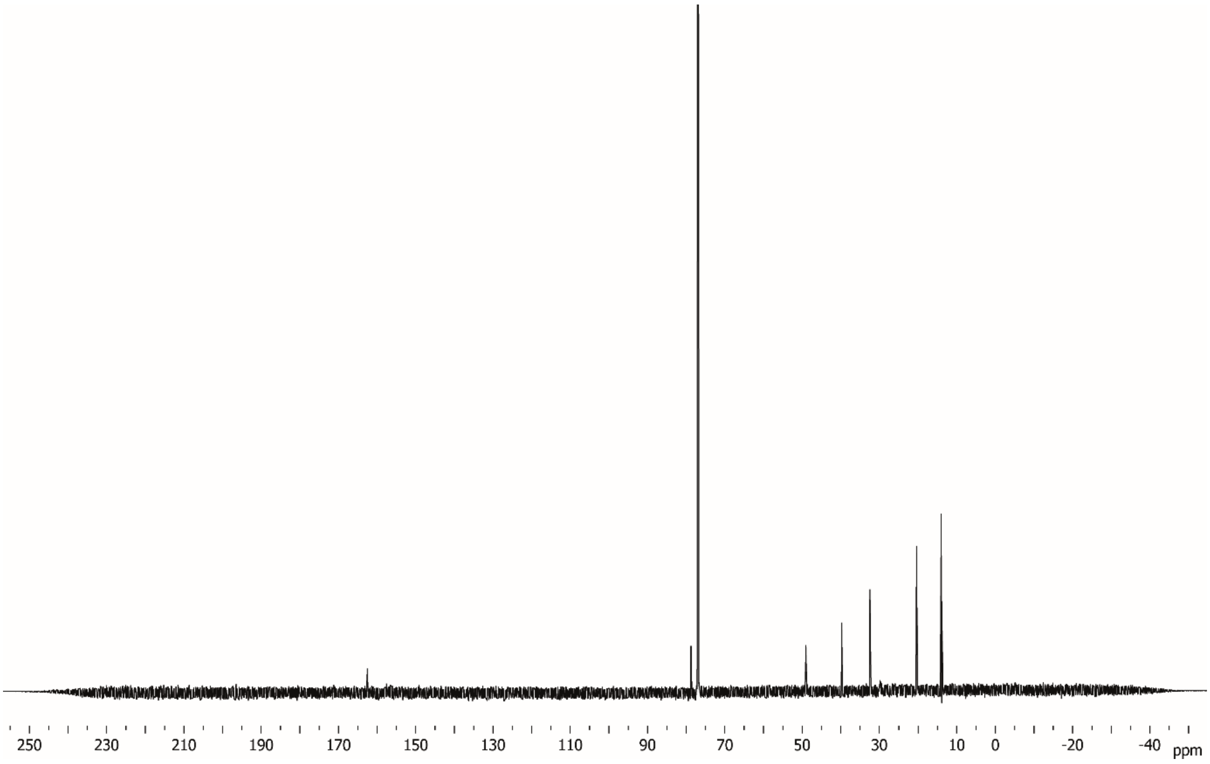

^{13}C NMR AUPy **1** (*150 MHz,* 30 mM, $CDCl_3$)

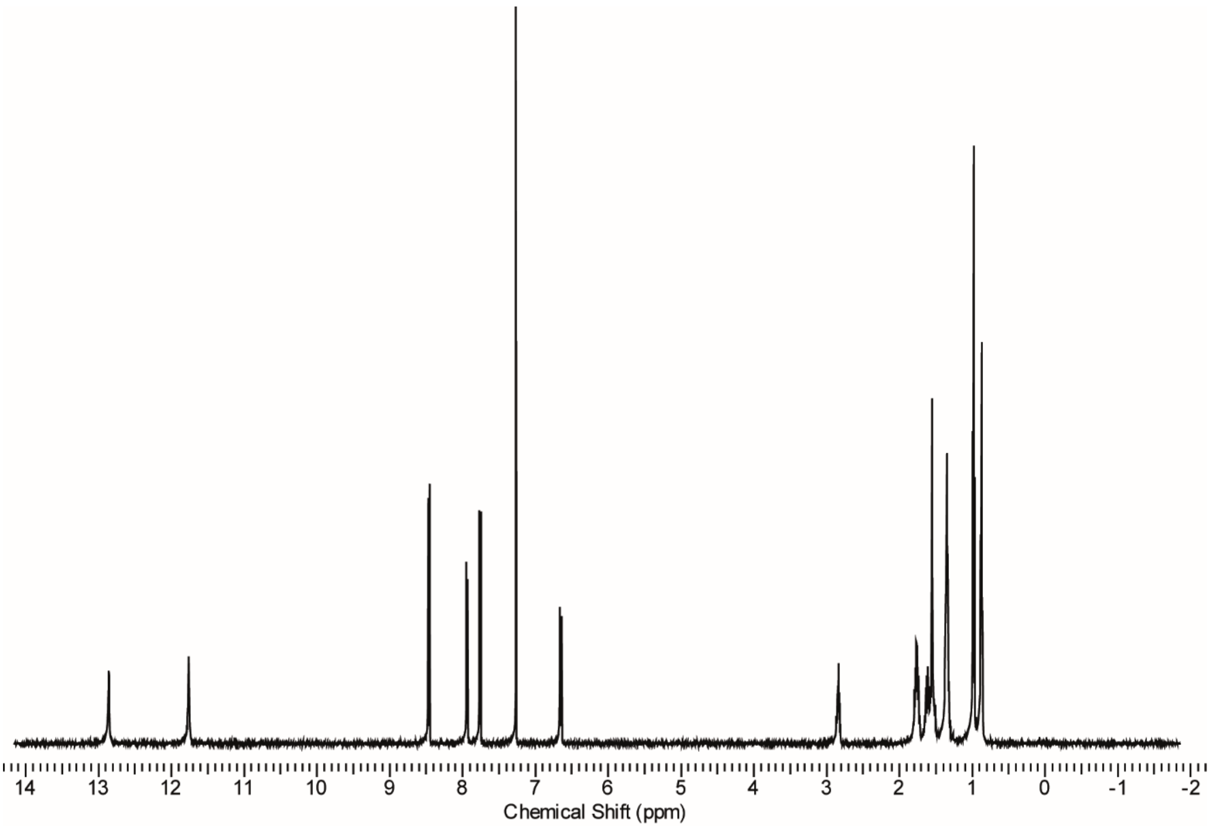

^{1}H NMR NAPyO **2** (*500 MHz,* 10 mM, $CDCl_3$)

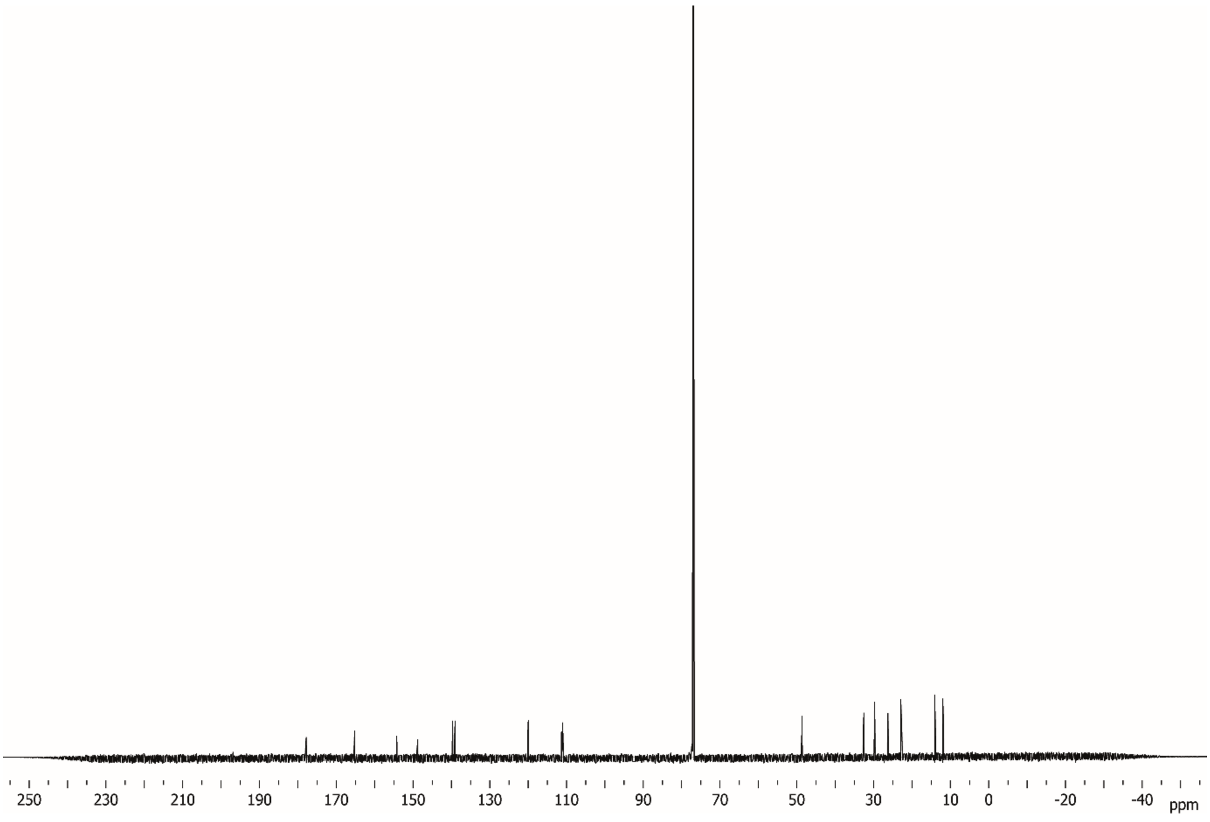

^{13}C NMR NAPyO **2** (*150 MHz,* 30 mM, $CDCl_3$)

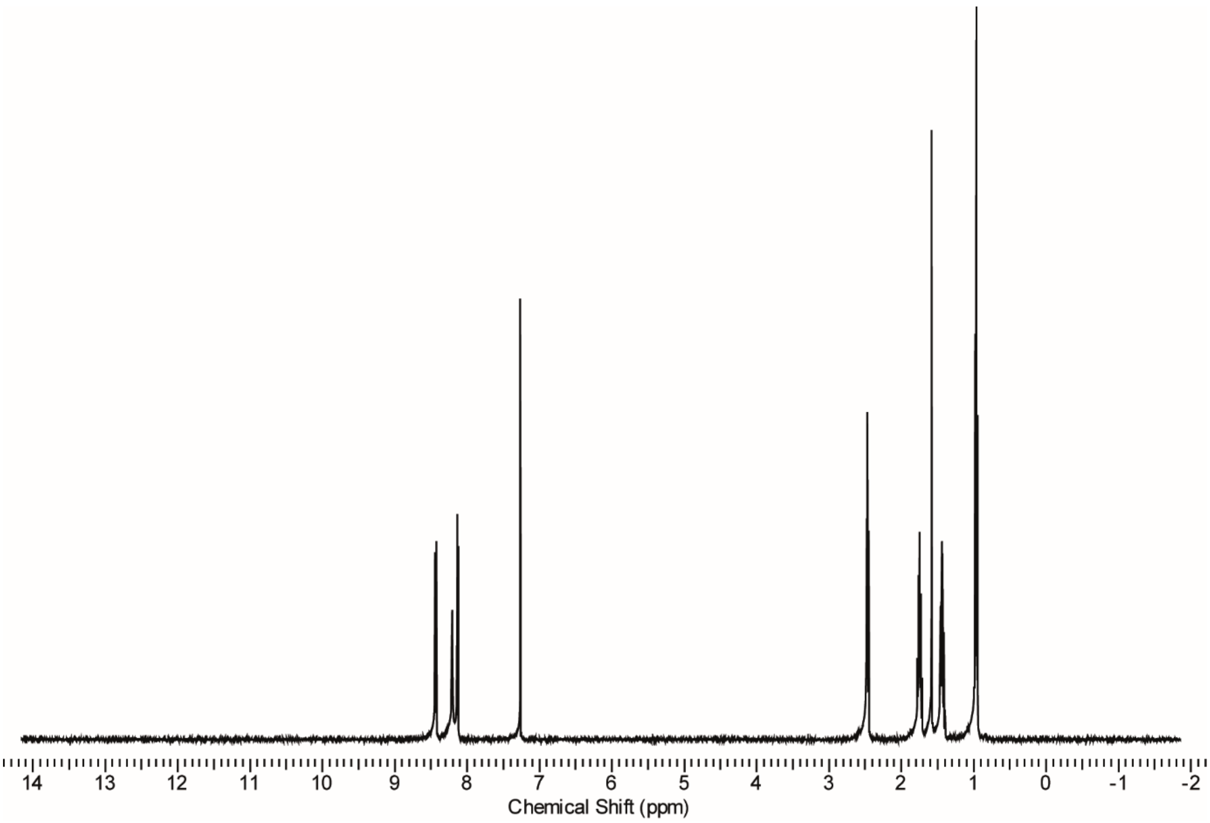

^{1}H NMR DAN **3** (*500 MHz,* 10 mM, $CDCl_3$)

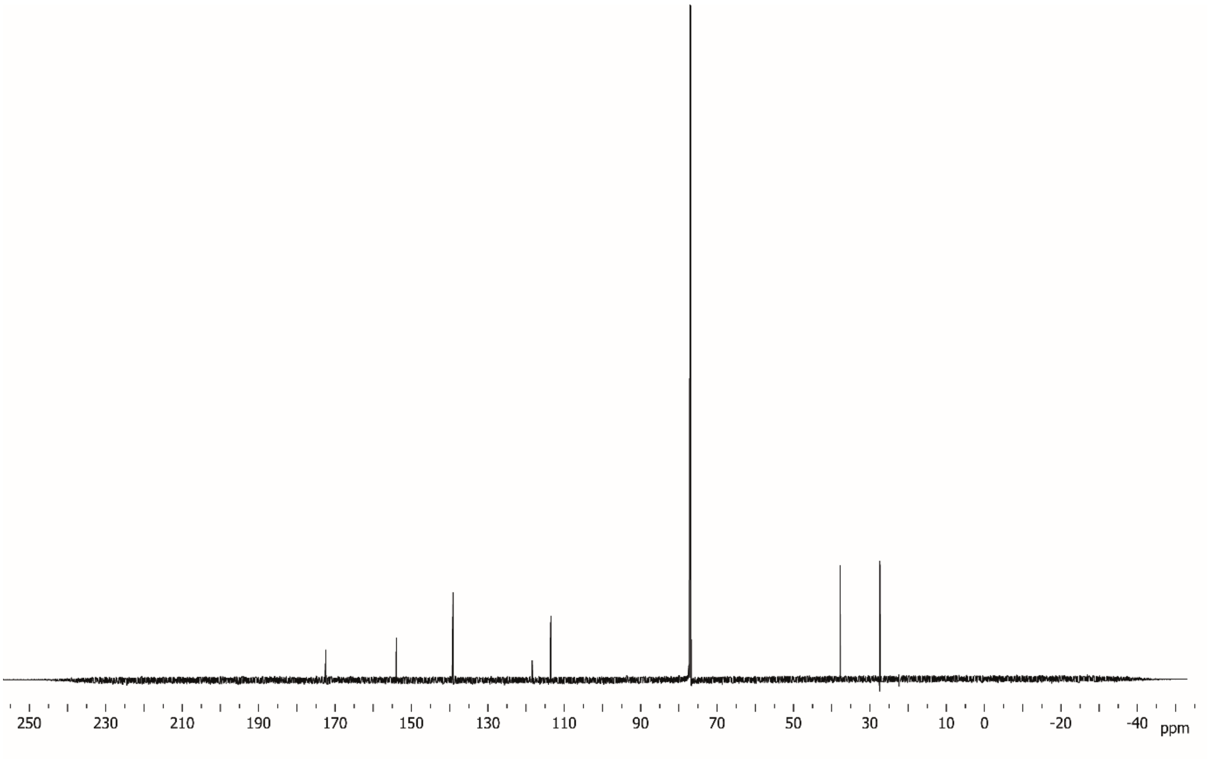

^{13}C NMR DAN **3** (*150 MHz,* 30 mM, $CDCl_3$)

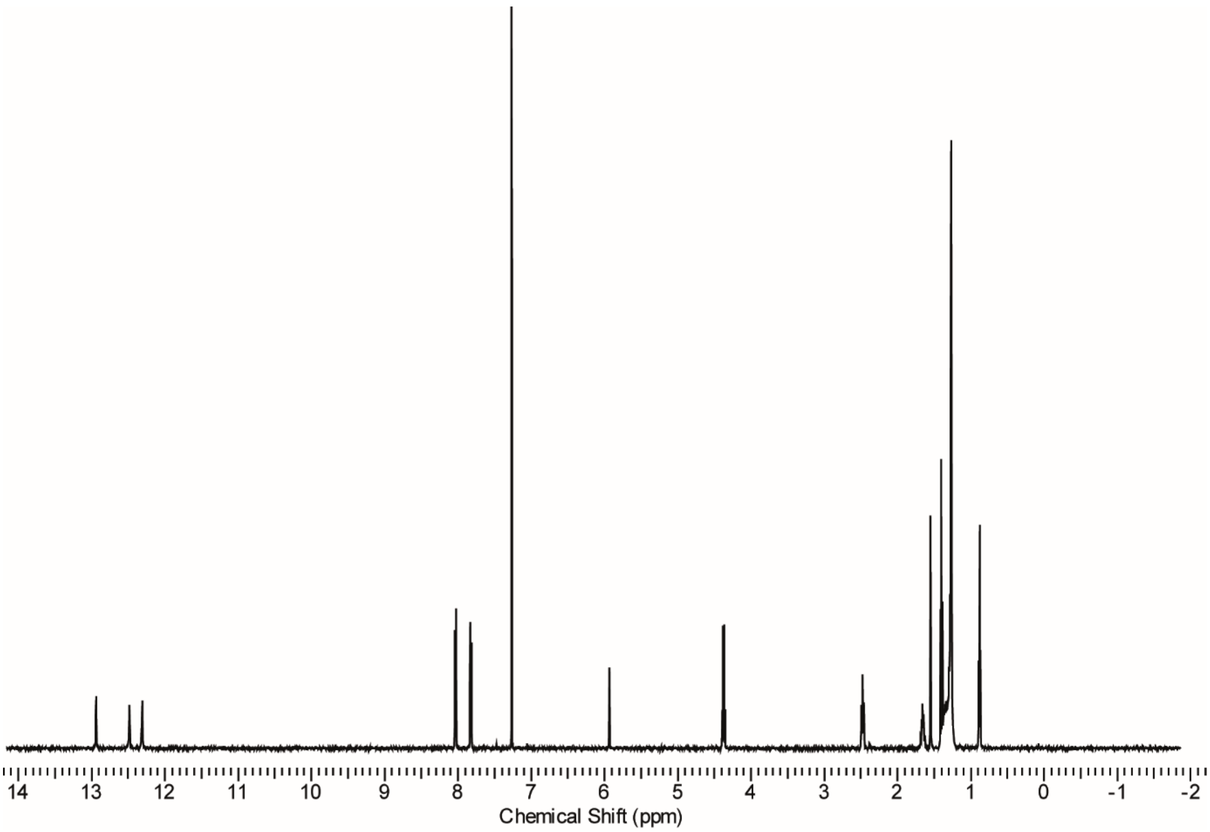

^{1}H NMR UPy **4** (*500 MHz,* 10 mM, $CDCl_3$)

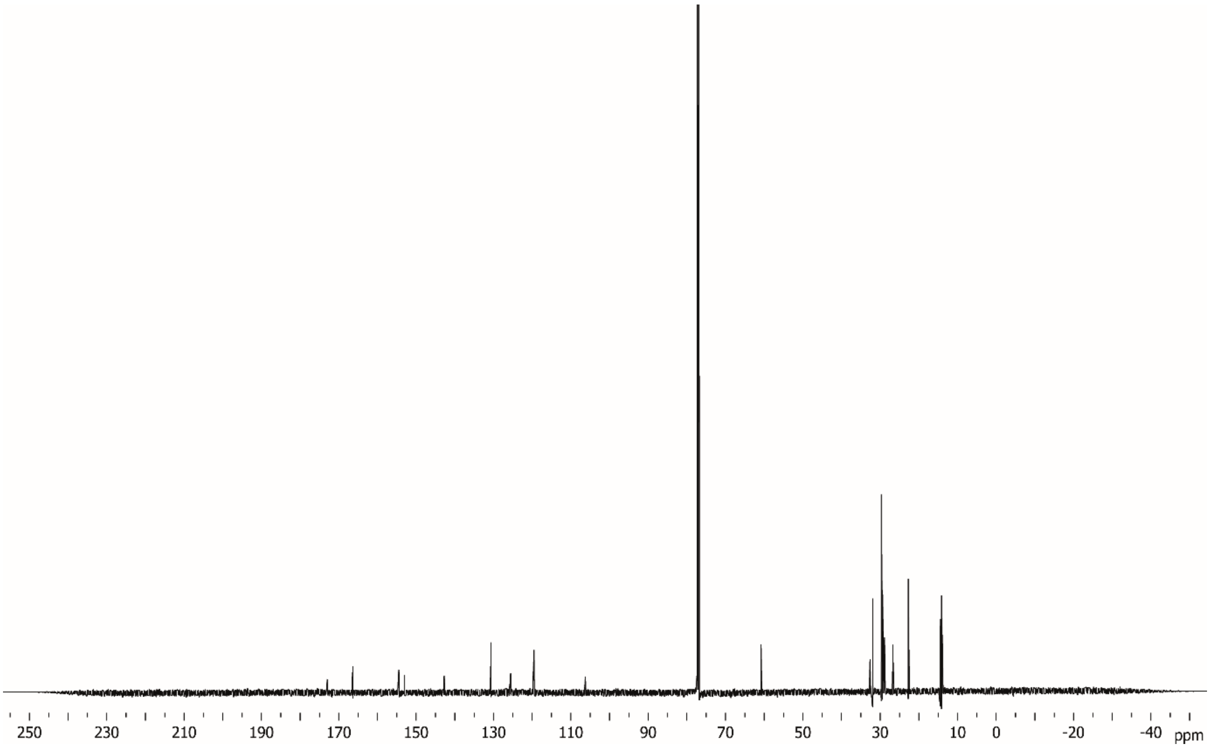

^{13}C NMR UPy **4** (*150 MHz,* 30 mM, $CDCl_3$)

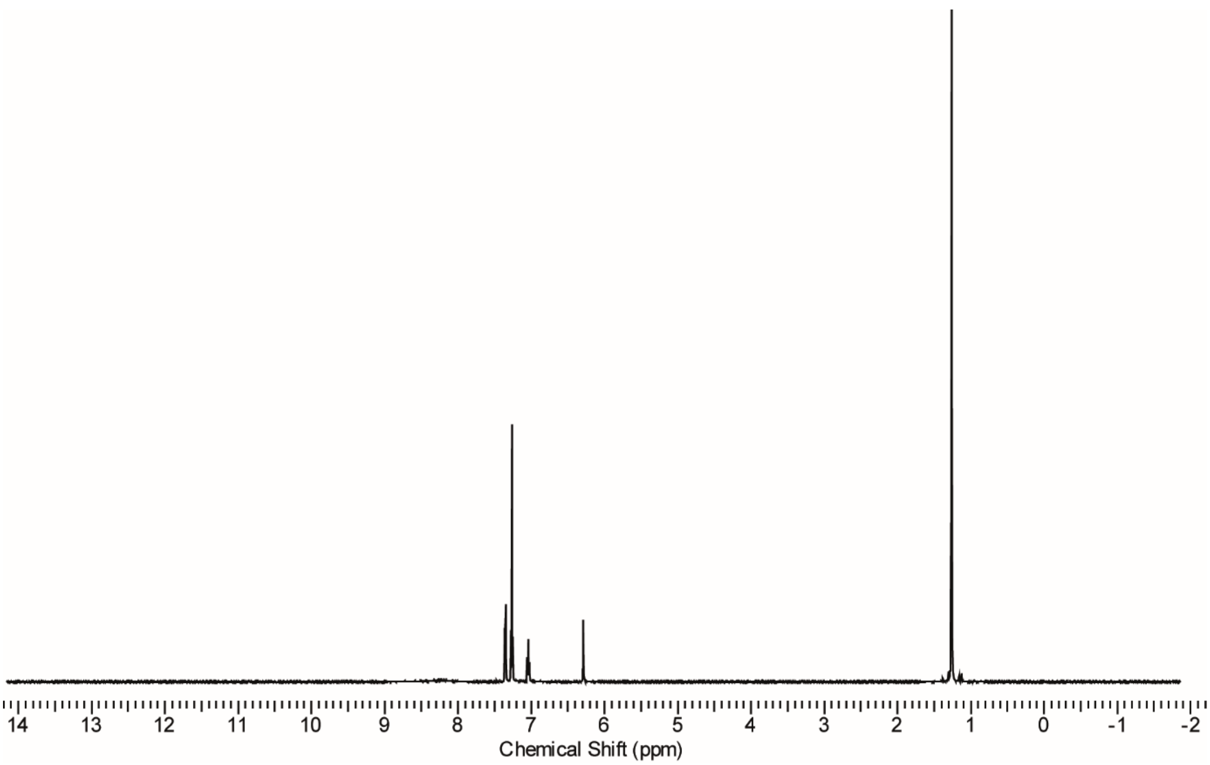

^{1}H NMR UIM **5** (*500 MHz,* 10 mM, $CDCl_3$)

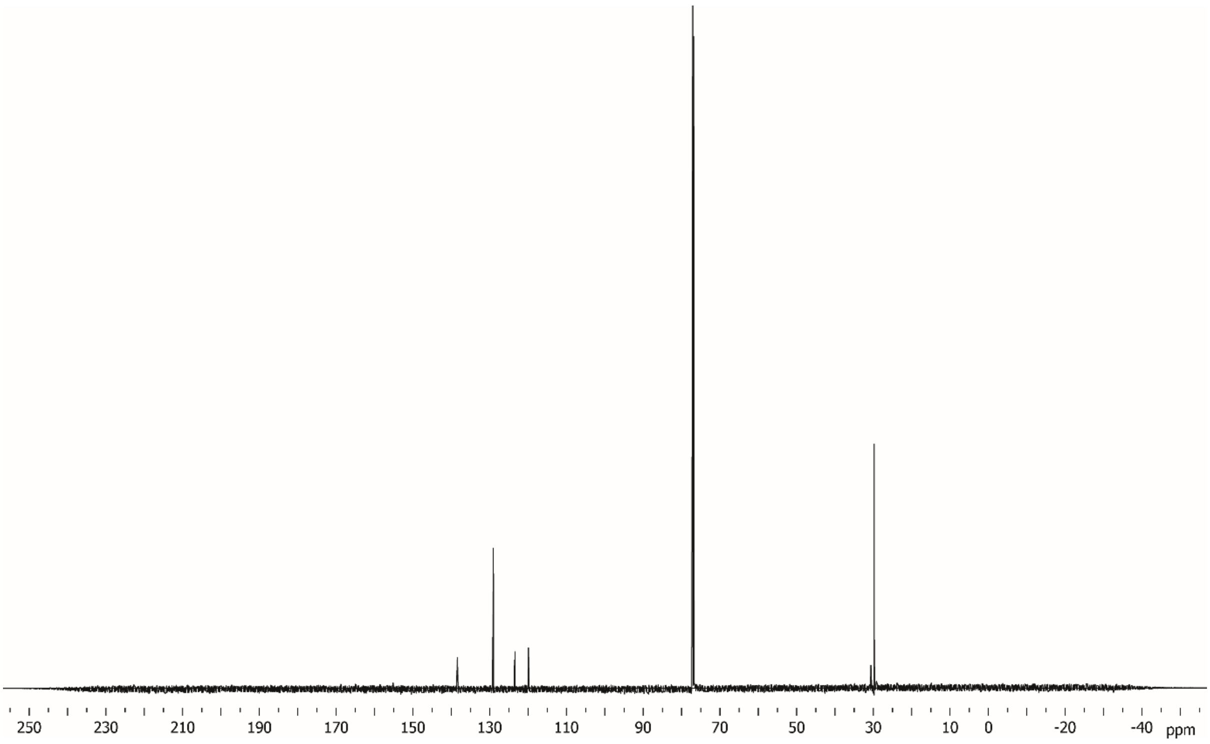

^{13}C NMR UIM **5** (*150 MHz,* 30 mM, $CDCl_3$)

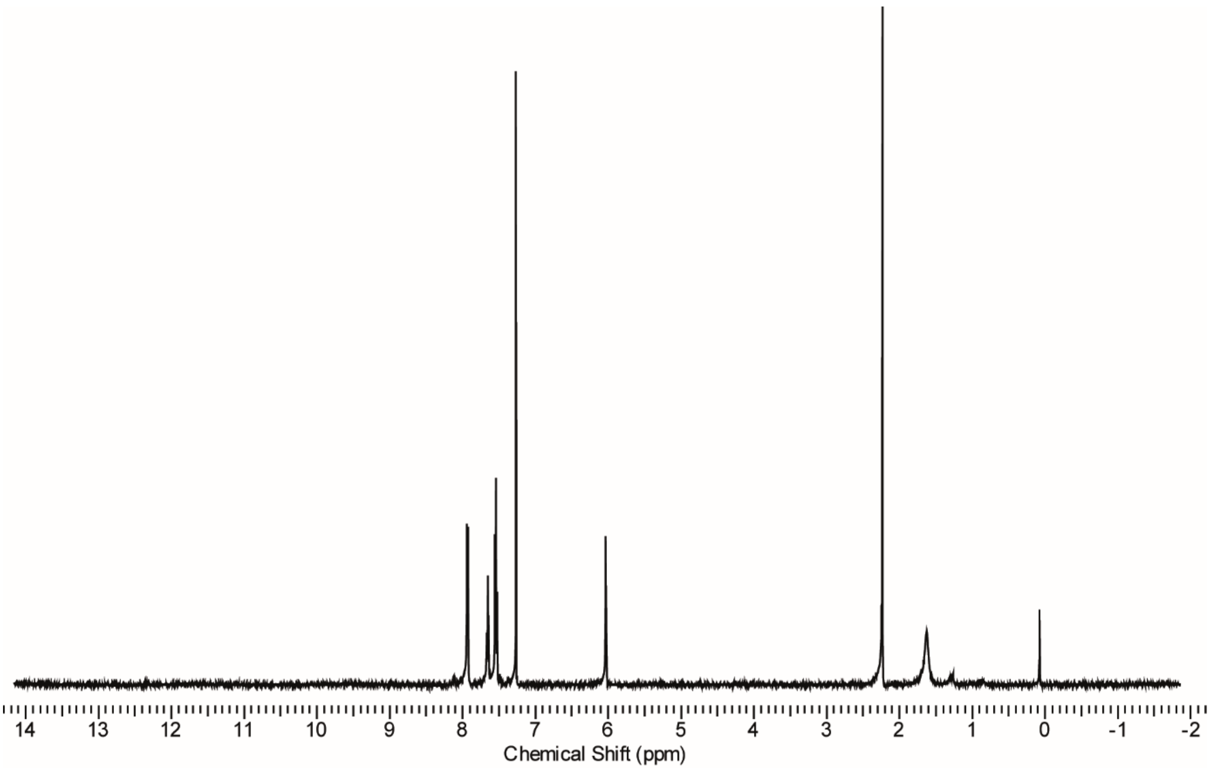

^{1}H NMR AIC **6** (*500 MHz,* 10 mM, $CDCl_3$)

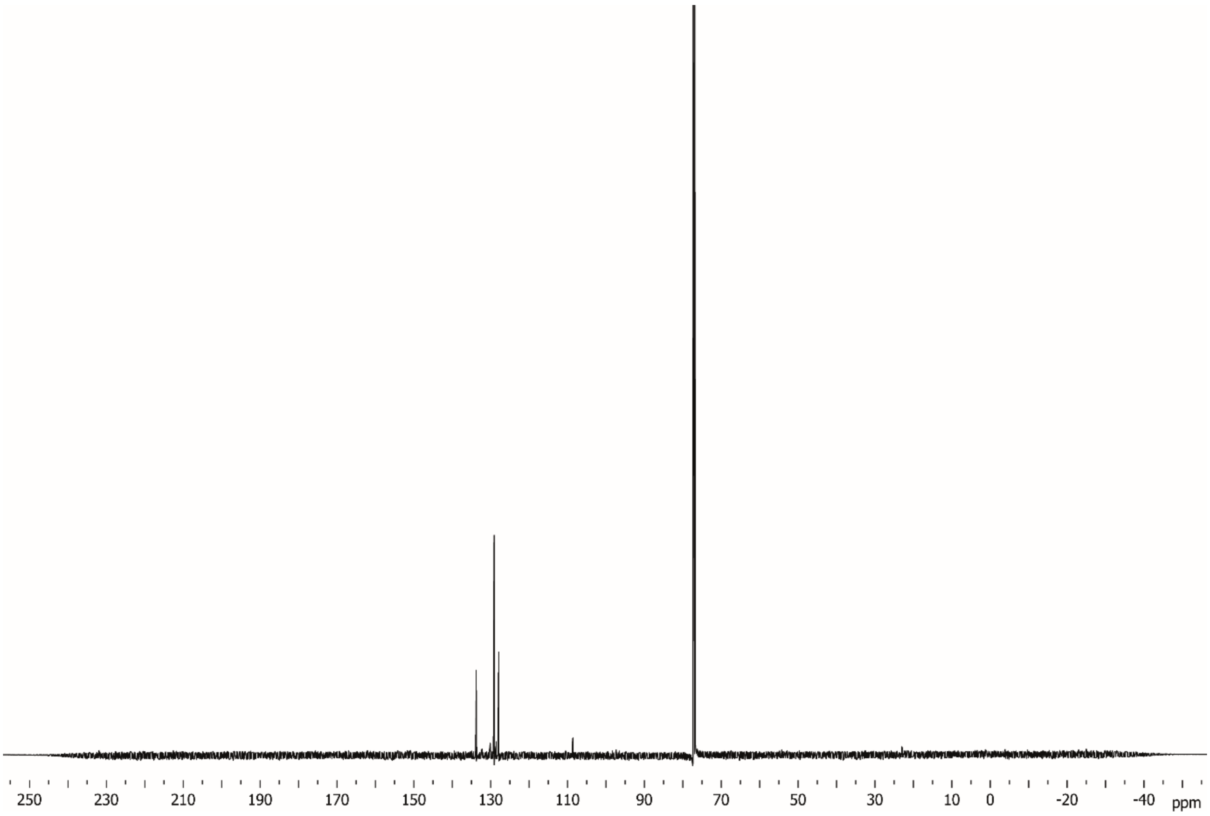

^{13}C NMR AIC **6** (*150 MHz,* 30 mM, $CDCl_3$)

4. Computational Data

All calculations were performed with the Density Functional Theory (DFT) based program Amsterdam Density Functional (ADF) 2017.208.[3] We used the BLYP Generalized Gradient Approximation (GGA) density functional, which is composed of the Becke[4] (B) exchange and Lee, Yang and Parr [5] (LYP) correlation functional. In order to describe the non-local dispersion interactions, we applied the DFT-D3(BJ) method developed by Grimme and coworkers,[6] which contains the damping function proposed by Becke and Johnson[7] and is essentially free of basis set superposition errors (BSSE) and other incompleteness effects.[8] The BLYP-D3(BJ) functional is in excellent agreement with the best available ab initio results for the hydrogen bond lengths and energies of biological hydrogen-bonded systems.[9] All integrals that are evaluated numerically, including the exchange- correlation integrals, were solved by using the Becke integration scheme with an integration accuracy of 'very good'.[10]

The Kohn-Sham Molecular Orbitals (KS MOs) were constructed from a linear combination of Slater-type orbitals (STOs), which have the correct cusp behaviour and long-range decay. We used the TZ2P basis set, which is of triple-ζ quality for all atoms and has been augmented with two sets of polarization functions, i.e. 2*p* and 3*d* on H and 3*d* and 4*f* on C, N and O. To speed up the computation, we treated the 1*s* core shells of C, N and O by the frozen-core approximation.[11] The molecular density was fitted by the systematically improvable Zlm fitting scheme with quality 'very good'.[12] The SCF procedure was considered to be converged if the difference between ρ^n and ρ^{n+1} was equal or smaller than 1e-6.

Geometries were optimized in chloroform in Cartesian coordinates. The chloroform solvent was modelled by using the implicit conductor-like screening model (COSMO), in which the solute molecule is surrounded by a dielectric medium.[13] The convergence criteria were 1e-6 for the changes in bond energy in Hartree, and 1e-5 for the nuclear gradient in Hartree/Ångström. All complexes were optimized with C_1 (i.e. without) symmetry constraints. All optimized structures have been verified to be true minima (zero imaginary frequencies).

The vibrational frequencies were obtained by evaluating the analytical second derivative of the total energy with respect to the nuclear displacements.[14] The frequencies were used for two different purposes, namely (1) the verification of minimum energy structures and (2) the derivation of the entropy ΔS and Gibbs free energy ΔG.

Voronoi deformation density (VDD) charges

The atomic charge distribution was analyzed by using the Voronoi Deformation Density (VDD) method.[15] The VDD method partitions the space into so-called Voronoi cells, which are non-overlapping regions of space that are closer to nucleus A than to any other nucleus. The charge distribution is determined by taking a fictitious promolecule as reference point, in which the electron density is simply the superposition of the atomic densities. The change in density in the Voronoi cell when going from this promolecule to the final molecular density of the interacting system is associated with the VDD atomic charge Q. Thus, the VDD atomic charge $Q_{\mathrm{A}}^{\mathrm{VDD}}$ of atom A is given by:

$$Q_{\mathrm{A}}^{\mathrm{VDD}} = -\int_{\text{Voronoi cell of A}} [\rho(\mathbf{r}) - \rho_{\text{promolecule}}(\mathbf{r})]d\mathbf{r}$$

So, instead of computing the amount of charge contained in an atomic volume, we compute the flow of charge from one atom to the other upon formation of the molecule. The physical interpretation is therefore straightforward. A positive atomic charge Q_{A} corresponds to the loss of electrons, whereas a negative atomic charge Q_{A} is associated with the gain of electrons in the Voronoi cell of atom A.

Cartesian coordinates monomers

Cartesian coordinates [in Å] and Gibbs free energies [in kcal mol^{-1}] of the monomers, computed at BLYP-D3(BJ)/TZ2P in implicit chloroform solvation at 298.15 K.

UPy DADA (C_1) [-2488.2]

```
N         2.273595   -0.186532    0.395247
H         3.470428    1.987878    0.017544
N         1.531535    1.965566   -0.236840
C         1.251408    0.680472    0.044563
H        -2.162074   -0.569007   -0.098296
O        -2.237878    0.377313   -0.342423
H         5.122010    1.420120    0.383682
N         4.128941    1.254182    0.286979
C         3.664614    0.009130    0.535147
O         4.368224   -0.957740    0.871613
C         0.476423    2.742456   -0.568394
N         0.021913    0.113941    0.022036
H         0.702144    3.782308   -0.795799
H         1.981261   -1.138700    0.593045
H        -1.664300    2.903505   -0.891017
C        -0.824646    2.272281   -0.623924
C        -0.992040    0.911074   -0.309439
```

UPy ADDA (C_1) [-2491.8]

```
N         1.308989   -0.138326   -0.249116
H         2.737512    2.809455   -0.182613
N        -0.572313    1.226243    0.148494
C        -0.041912    0.030178   -0.038884
C        -2.201551   -1.132476    0.167693
O        -2.801697   -2.218214    0.155433
H         1.027534    2.341099    0.020353
N         2.009587    2.107752   -0.142801
C         2.361482    0.817828   -0.312121
O         3.510065    0.401731   -0.515630
C        -1.927615    1.274477    0.349617
N        -0.796682   -1.111599   -0.035291
H        -2.331713    2.273560    0.500477
H         1.652754   -1.083918   -0.385720
H        -3.821382    0.275571    0.532727
C        -2.753497    0.182738    0.368864
H        -0.360979   -2.020900   -0.181488
```

UPy AADD (C_1) [-2489.7]

```
N         1.369624   -0.026354   -0.043398
H        -0.254243    1.907381   -0.075239
N         1.896786   -2.311989    0.208700
C         2.318562   -1.042959   -0.059294
C         4.543376   -1.712047   -0.356943
O         5.728484   -1.415698   -0.614585
H        -1.683423    0.996699    0.322324
N        -0.684859    1.022912    0.161300
C         0.001061   -0.147041    0.215596
O        -0.537742   -1.238833    0.478014
C         2.812158   -3.339791    0.199565
N         3.557776   -0.713026   -0.331602
H         2.415556   -4.324662    0.419468
H         1.762547    0.887045   -0.251701
H         4.851482   -3.877431   -0.084987
C         4.114645   -3.081696   -0.072971
H         0.897350   -2.434521    0.405634
```

UPy ADA (C_1) [-2487.6]

```
N         1.263351    0.019159   -0.096075
H         0.866711   -2.472597   -0.177567
N        -0.745951    1.131854    0.072463
C        -0.117719   -0.058853   -0.016206
H        -1.994420   -3.177367   -0.036016
O        -2.678265   -2.479820    0.035879
H         2.566265   -2.974706   -0.303297
N         1.860223   -2.254621   -0.226730
```

C	2.267921	-0.964654	-0.198632
O	3.453273	-0.599303	-0.256441
C	-2.089035	1.080116	0.152159
N	-0.715759	-1.269206	-0.031670
H	-2.602301	2.037402	0.223951
H	1.631390	0.965828	-0.079080
H	-3.894034	-0.133707	0.210947
C	-2.812473	-0.106847	0.145988
C	-2.050426	-1.278267	0.049277

**AUPy DADA (C_1)**			**[-2757.4]**
N	2.271343	-0.237919	0.191726
H	3.463160	1.958219	-0.028061
N	1.506209	1.970584	-0.113050
C	1.229171	0.667011	0.026901
H	-2.178041	-0.548195	-0.003450
O	-2.275886	0.418838	-0.124410
H	5.131341	1.342923	0.122827
N	4.131008	1.196234	0.100193
C	3.668478	-0.068121	0.235198
O	4.387079	-1.070285	0.392972
C	0.445875	2.806619	-0.271441
N	0.003026	0.101262	0.029602
H	1.679768	4.439149	-0.252917
H	1.981176	-1.205356	0.295033
H	-1.731785	2.980442	-0.405513
C	-0.876001	2.326422	-0.281303
C	-1.022499	0.951788	-0.124858
N	0.737096	4.126889	-0.451935
H	-0.002107	4.814283	-0.383629

**AUPy ADDA (C_1)**			**[-2760.6]**
N	1.356352	-0.067498	-0.272036
H	0.939078	2.399901	-0.082797
N	-0.594629	1.191394	0.110901
C	-0.003833	0.025324	-0.051517
C	-2.091944	-1.241431	0.213279
O	-2.617212	-2.372914	0.236357
H	2.616369	2.954934	-0.325817
N	1.930077	2.215801	-0.247174
C	2.350324	0.940875	-0.379294
O	3.519860	0.586445	-0.583987
C	-1.956097	1.188486	0.327089
N	-0.683138	-1.155879	-0.012790
H	-1.891278	3.217380	0.595629
H	1.750676	-0.996347	-0.382734
H	-3.784134	0.052926	0.563684
C	-2.714142	0.022931	0.388160
H	-0.201733	-2.042481	-0.143764
N	-2.510830	2.430615	0.445367
H	-3.459990	2.520020	0.784503

**AUPy AADD (C_1)**			**[-2753.6]**
N	1.361622	0.009213	-0.039177
H	-0.236137	1.949331	-0.277976
N	1.825152	-2.287544	0.180447
C	2.303611	-1.016171	0.028213
C	4.529035	-1.726660	0.007756
O	5.743935	-1.430101	-0.069935
H	-1.706910	1.038758	-0.110217
N	-0.696214	1.072213	-0.070081
C	-0.027642	-0.107480	0.015613
O	-0.606216	-1.204134	0.149244
C	2.709916	-3.347840	0.247880
N	3.564244	-0.694637	-0.058747
N	2.143058	-4.596881	0.332672
H	1.783694	0.927257	-0.143598
H	4.777849	-3.889511	0.226266
C	4.055451	-3.081947	0.169863
H	0.801687	-2.385157	0.213635
H	1.219308	-4.667890	0.747408
H	2.768817	-5.350340	0.595458

**AUPy ADA (C_1)**			**[-2757.2]**
N	1.279986	0.017810	-0.262739
H	0.948560	-2.433407	0.205426
N	-0.739393	1.105915	-0.172393

C	-0.093432	-0.065628	-0.062002
H	-1.860409	-3.131771	0.705536
O	-2.566505	-2.454368	0.685557
H	2.651581	-2.932103	0.063321
N	1.932125	-2.221869	0.039762
C	2.302227	-0.948779	-0.232216
O	3.471370	-0.587934	-0.452572
C	-2.081674	1.076962	0.012815
N	-0.644274	-1.263719	0.216857
H	-2.196781	3.109867	-0.188561
H	1.618626	0.952433	-0.469801
H	-3.837876	-0.158570	0.463614
C	-2.764812	-0.121675	0.312694
C	-1.978140	-1.259551	0.399350
N	-2.743428	2.258921	-0.134304
H	-3.707159	2.337707	0.162236

NAPyO DADA (C_1) **[-3158.8]**

N	5.941174	0.525141	-0.035304
C	7.247047	0.127213	0.121495
O	7.636528	-1.013867	0.364419
H	0.862891	-3.105714	0.332587
C	2.312025	-1.500534	0.117828
C	2.468088	-0.110575	-0.140882
N	3.652933	0.516228	-0.183836
C	4.755019	-0.221599	0.031398
C	4.728010	-1.609041	0.297925
C	3.492106	-2.235312	0.337707
H	7.937255	0.981138	0.000040
C	-0.104608	-1.255900	-0.090174
C	0.024507	0.170594	-0.358356
H	3.426876	-3.301642	0.539511
H	5.792369	1.512122	-0.234900
N	1.340377	0.648480	-0.362931
O	-0.923721	0.949265	-0.573000
H	5.651712	-2.146006	0.462418
C	0.985088	-2.042572	0.134778
H	-1.114003	-1.654811	-0.080157
H	1.468554	1.642416	-0.547220

NAPyO DAAD (C_1) **[-3149.7]**

N	-2.947768	-2.376551	-0.152862
C	-3.946176	-3.313516	-0.251265
O	-5.154714	-3.091165	-0.320770
H	-3.693108	3.819397	0.033562
C	-2.957999	1.774707	0.006250
C	-1.771125	0.968159	0.058741
N	-1.822811	-0.387732	0.002513
C	-3.016170	-0.972542	-0.103525
C	-4.248553	-0.266843	-0.162978
C	-4.200924	1.109373	-0.106834
H	-3.527440	-4.335989	-0.261536
C	-1.556108	3.735694	0.181449
C	-0.446328	2.844419	0.225329
H	-5.118240	1.692361	-0.149041
H	-1.996529	-2.735954	-0.102860
N	-0.528211	1.528622	0.168216
O	0.791846	3.407668	0.335147
H	1.441578	2.674260	0.352840
C	-2.809666	3.186305	0.071495
H	-1.389830	4.806796	0.234852
H	-5.178625	-0.811751	-0.249186

NAPyO AADD (C_1) **[-3146.5]**

N	6.092548	0.210401	0.072172
C	6.344006	1.563302	0.027344
O	5.478532	2.437444	-0.040088
H	0.837388	-3.208408	0.021134
C	2.330853	-1.621915	0.009668
C	2.449086	-0.188303	-0.045711
N	3.737733	0.331394	-0.020357
C	4.856984	-0.430843	0.052989
C	4.758081	-1.820338	0.107210
C	3.487944	-2.401118	0.084627
H	7.422776	1.783035	0.058782
C	-0.054013	-1.265616	-0.091673
C	0.152821	0.190462	-0.143938

H	3.395811	-3.484059	0.126494
H	6.903255	-0.399557	0.126185
N	1.455635	0.680516	-0.117691
O	-0.820508	0.980625	-0.210468
H	3.858958	1.349663	-0.057644
C	0.992187	-2.131941	-0.017894
H	-1.082651	-1.615699	-0.114344
H	5.659483	-2.420260	0.165807

DAN (C_1) **[-3606.5]**

N	-2.241335	-0.221222	0.429696
C	-2.704302	1.070132	0.363387
O	-2.056479	2.064444	0.038570
H	3.552115	-2.139951	-0.873563
C	1.461513	-1.944744	-0.328752
C	0.303863	-2.679127	0.099087
N	-0.888315	-2.065511	0.334529
C	-0.966451	-0.748569	0.158991
C	0.121252	0.069084	-0.261970
C	1.329122	-0.545291	-0.501782
H	-3.771134	1.120712	0.646444
C	2.688379	-4.030473	-0.352691
C	1.486366	-4.665393	0.072663
H	2.188475	0.037856	-0.824923
H	-2.916150	-0.926487	0.719195
N	0.339592	-4.026357	0.291266
H	1.954144	-8.017381	0.450624
H	0.485785	-6.359253	0.598663
C	2.658794	-2.668662	-0.548921
O	3.540140	-6.867024	-0.150481
C	2.368634	-7.026955	0.189862
N	1.409328	-6.050437	0.301461
H	-0.021641	1.134326	-0.381932
H	3.581495	-4.619820	-0.509344

Cartesian coordinates dimers

Cartesian coordinates [in Å] and Gibbs free energies [in kcal mol^{-1}] of the dimers, computed at BLYP-D3(BJ)/TZ2P in implicit chloroform solvation at 298.15 K.

UPy DADA-ADAD (C_1) [-4986.2]

N	2.771044	1.444028	-0.911412
H	3.818091	3.624296	-1.615204
N	2.086454	3.700840	-0.813619
C	1.829277	2.406796	-0.565766
H	-1.403499	1.371928	1.044883
O	-1.364242	2.389812	0.931098
H	5.343412	2.937417	-2.258363
N	4.435009	2.834163	-1.824945
C	4.019172	1.595500	-1.508450
O	4.719097	0.572162	-1.733448
C	1.123447	4.580058	-0.448520
N	0.705980	1.914030	0.008392
H	1.333885	5.628425	-0.650379
H	2.525243	0.462333	-0.698070
H	-0.826918	4.902976	0.434396
C	-0.059818	4.193815	0.144373
C	-0.235746	2.807543	0.362045
N	0.172584	-0.990711	0.544568
H	-0.876040	-3.171348	1.245360
N	0.860967	-3.246709	0.455019
C	1.116625	-1.953004	0.203842
H	4.349192	-0.918203	-1.407256
O	4.313433	-1.935356	-1.285971
H	-2.404862	-2.485004	1.880717
N	-1.495203	-2.381635	1.449974
C	-1.078108	-1.142731	1.136058
O	-1.779446	-0.119696	1.358014
C	1.826598	-4.125375	0.095557
N	2.240425	-1.460146	-0.369251
H	1.617416	-5.173461	0.300151
H	0.418745	-0.008935	0.331987
H	3.780071	-4.447664	-0.780691
C	3.010892	-3.738936	-0.495152
C	3.184620	-2.353142	-0.717585

UPy DDAA-AADD (C_1) [-4990.5]

N	1.433205	-0.094906	0.036032
H	-0.185677	1.858545	0.015536
N	1.856609	-2.403752	0.001813
C	2.340411	-1.127985	0.025762
C	4.556168	-1.894055	0.022889
O	5.791602	-1.647306	0.034147
H	-1.654046	0.863263	0.020588
N	-0.643331	0.924408	0.034438
C	0.029635	-0.238200	0.028594
O	-0.527145	-1.361820	0.020401
C	2.715548	-3.470753	-0.014070
N	3.636632	-0.847646	0.038507
H	2.251777	-4.450633	-0.033160
H	1.842811	0.861752	0.046606
H	4.760164	-4.080543	-0.018024
C	4.054789	-3.257599	-0.005592
H	0.825575	-2.478487	-0.001255
N	4.789468	1.904825	0.161423
H	6.409158	-0.048195	0.157333
N	4.366394	4.213784	0.187520
C	3.883115	2.938473	0.139927
C	1.669994	3.706366	0.046375
O	0.435972	3.460478	-0.017092
H	7.874099	0.944998	0.276752
N	6.864184	0.884261	0.233710
C	6.191275	2.046907	0.234108
O	6.746660	3.169564	0.296243
C	3.508533	5.281502	0.164528
N	2.588353	2.659122	0.073374
H	3.971728	6.260964	0.205918
H	4.380783	0.948801	0.116077

H	1.466482	5.893152	0.073633
C	2.170918	5.069553	0.093849
H	5.396087	4.287387	0.242015

AUPy DADA-ADAD (C_1) [-5523.4]

N	2.413332	-0.280609	0.202062
H	3.481141	1.990282	-0.023229
N	1.573701	1.899414	-0.095627
C	1.335874	0.588631	0.040268
H	-2.161225	-0.734257	-0.001339
O	-2.168914	0.278764	-0.118345
H	5.182627	1.447410	0.122435
N	4.188491	1.261694	0.101154
C	3.776897	-0.013103	0.236002
O	4.587388	-0.964966	0.391522
C	0.491176	2.707813	-0.253164
N	0.126826	-0.013770	0.041109
H	1.680030	4.371148	-0.202607
H	2.180984	-1.284352	0.306510
H	-1.683612	2.820500	-0.388585
C	-0.809281	2.190800	-0.265198
C	-0.939571	0.807490	-0.112926
N	0.751695	4.037862	-0.434055
H	-0.005865	4.702697	-0.341193
N	-0.344937	-2.955371	0.359974
H	-1.410963	-5.230197	0.551430
N	0.494592	-5.135194	0.659437
C	0.732691	-3.824992	0.518500
H	4.229968	-2.502514	0.556666
O	4.237495	-3.515182	0.676690
H	-3.111437	-4.689459	0.387335
N	-2.118546	-4.501103	0.431465
C	-1.707661	-3.224715	0.310300
O	-2.518123	-2.272865	0.154565
C	1.577582	-5.944595	0.808465
N	1.941962	-3.223057	0.513665
H	0.369087	-7.567935	1.103596
H	-0.112608	-1.951569	0.256035
H	3.752598	-6.057795	0.939420
C	2.878007	-5.427520	0.821010
C	3.008333	-4.044285	0.668050
N	1.321527	-7.284127	0.906849
H	2.055762	-7.901211	1.230302

AUPy DDAA-AADD (C_1) [-5520.6]

N	1.413878	-0.035950	-0.040150
H	-0.205603	1.910078	-0.220712
N	1.829859	-2.328637	0.189261
C	2.326420	-1.065411	0.025720
C	4.524844	-1.861881	-0.003749
O	5.763685	-1.614682	-0.089259
H	-1.669257	0.924893	-0.004053
N	-0.659466	0.985397	-0.050366
C	0.015756	-0.171210	0.049380
O	-0.541423	-1.286241	0.213250
C	2.671791	-3.415566	0.256553
N	3.614161	-0.796110	-0.070810
N	2.071465	-4.640391	0.361223
H	1.819588	0.915087	-0.177669
H	4.729286	-4.020858	0.216890
C	4.027733	-3.195568	0.164575
H	0.794176	-2.373907	0.242775
H	1.125539	-4.686560	0.723869
H	2.664283	-5.421108	0.617342
N	4.749231	1.917783	-0.368820
H	6.368302	-0.030228	-0.206608
N	4.331862	4.205369	-0.641694
C	3.835124	2.941742	-0.481908
C	1.633466	3.726393	-0.555912
O	0.393467	3.475044	-0.506815
H	7.835394	0.967666	-0.314627
N	6.825203	0.905575	-0.280947
C	6.149144	2.059549	-0.402870
O	6.707396	3.178531	-0.532828
C	3.488044	5.285082	-0.771995
N	2.545825	2.666893	-0.434060
N	4.084891	6.497048	-0.986877

H	4.342638	0.963228	-0.261696
H	1.427754	5.880177	-0.821215
C	2.130567	5.059679	-0.727124
H	5.368511	4.253986	-0.665380
H	5.051648	6.617566	-0.705450
H	3.503902	7.320118	-0.879368

NAPyO DADA-ADAD (C_1) [-6321.1]

N	5.882096	0.612192	-0.059874
C	7.198830	0.240698	0.057699
O	7.643909	-0.902088	0.182173
H	1.055385	-3.344664	-0.040810
C	2.379000	-1.625904	-0.103332
C	2.437373	-0.209670	-0.225097
N	3.583565	0.495185	-0.208350
C	4.741083	-0.187182	-0.068303
C	4.793789	-1.596915	0.060026
C	3.605859	-2.301918	0.040837
H	7.855252	1.128402	0.026584
C	-0.045592	-1.534294	-0.277489
C	-0.002949	-0.091722	-0.403760
H	3.609208	-3.384873	0.137289
H	5.732141	1.638260	-0.153859
N	1.259003	0.489972	-0.368783
O	-1.024920	0.628712	-0.539103
H	5.751921	-2.084895	0.169004
C	1.098749	-2.261478	-0.134415
H	-1.026739	-1.997229	-0.303373
H	1.295967	1.522859	-0.460227
N	-1.091308	3.393194	-0.821852
C	-2.406234	3.762524	-0.964090
O	-2.849342	4.902948	-1.114426
H	3.732803	7.353188	-0.805569
C	2.410001	5.633439	-0.754020
C	2.351437	4.217450	-0.629545
N	1.205852	3.511848	-0.654392
C	0.049326	4.193009	-0.807904
C	-0.002927	5.602173	-0.942421
C	1.184223	6.308133	-0.912809
H	-3.063087	2.875348	-0.927686
C	4.832811	5.544023	-0.555511
C	4.790178	4.101459	-0.429250
H	1.181099	7.390815	-1.012289
H	-0.942879	2.368935	-0.707960
N	3.528976	3.518851	-0.474474
O	5.811536	3.381776	-0.285491
H	-0.960243	6.089010	-1.063104
C	3.689371	6.270093	-0.710968
H	5.813278	6.007800	-0.520369
H	3.492223	2.485708	-0.385978

NAPyO DDAA-AADD (C_1) [-6310.1]

N	5.730000	0.886040	0.496376
C	7.039013	0.542019	0.756590
O	7.483420	-0.586082	0.965664
H	1.019534	-3.212308	0.066979
C	2.286543	-1.447322	0.153262
C	2.252776	-0.020510	0.063915
N	3.444611	0.661639	0.190737
C	4.642601	0.044852	0.403262
C	4.694513	-1.356420	0.503891
C	3.518910	-2.080163	0.374693
H	7.678827	1.441343	0.758958
C	-0.099165	-1.407631	-0.202351
C	-0.058572	0.045837	-0.274096
H	3.548255	-3.165182	0.445271
H	5.578631	1.927548	0.341794
N	1.143379	0.705919	-0.134880
O	-1.129519	0.707635	-0.463863
H	3.401046	1.716305	0.112655
C	1.038475	-2.126279	0.007536
H	-1.068194	-1.884014	-0.319183
H	5.649332	-1.830425	0.675030
N	-1.297185	3.372202	-0.345269
C	-2.628608	3.724878	-0.396566
O	-3.097450	4.862221	-0.374877
H	3.432635	7.462802	-0.092011

C	2.160948	5.699576	-0.135359
C	2.200937	4.270382	-0.107774
N	1.003281	3.590510	-0.180535
C	-0.207548	4.212331	-0.270710
C	-0.267882	5.616781	-0.288143
C	0.914695	6.337811	-0.223047
H	-3.262160	2.823449	-0.460754
C	4.568256	5.651748	0.013732
C	4.531577	4.196787	0.046487
H	0.880139	7.424803	-0.241876
H	-1.125431	2.323096	-0.382822
N	3.321562	3.539745	-0.016346
O	5.613253	3.530998	0.133728
H	1.053506	2.533304	-0.167467
C	3.417621	6.375286	-0.071006
H	5.544386	6.125400	0.063186
H	-1.233397	6.095030	-0.356397

UPy-NAPyO DDAA-AADD (C_1) [-5649.8]

N	-2.271160	-0.240549	-0.173620
C	-3.544881	0.263923	-0.327350
O	-4.577642	-0.159500	0.189417
H	-0.825737	-5.178233	3.393277
C	-0.829815	-3.465752	2.052349
C	0.030975	-2.655160	1.244783
N	-0.545223	-1.611520	0.546524
C	-1.878161	-1.321403	0.588235
C	-2.735214	-2.108437	1.373316
C	-2.198290	-3.167238	2.092973
H	-3.533036	1.136560	-1.002976
C	1.131874	-4.733792	2.650860
C	1.947231	-3.867816	1.806008
H	-2.853259	-3.782843	2.705544
H	-1.533382	0.288330	-0.713948
N	1.351072	-2.830324	1.113547
O	3.196460	-4.066061	1.707336
H	0.098416	-1.024759	-0.040120
C	-0.209771	-4.536789	2.766637
H	1.643080	-5.535284	3.176425
H	-3.787511	-1.868479	1.396453
N	3.142307	-1.201881	-0.570779
H	4.185485	-3.124133	0.709431
N	3.328394	0.627037	-2.030833
C	2.546805	-0.164191	-1.240291
C	0.636641	1.086904	-1.771663
O	-0.604413	1.286187	-1.642676
H	5.836761	-2.854076	0.103084
N	4.859303	-2.589183	0.116345
C	4.514939	-1.530083	-0.631821
O	5.326319	-0.877127	-1.331748
C	2.783630	1.677936	-2.718523
N	1.240666	0.034145	-1.096592
H	3.473631	2.256041	-3.322854
H	2.506939	-1.779627	0.027528
H	0.994246	2.761045	-3.146106
C	1.455757	1.936727	-2.614867
H	4.328057	0.360425	-2.054540

UPy-NAPyO ADDA-DAAD (C_1) [-5650.0]

N	5.725323	-2.326581	-1.890020
C	6.642800	-3.089716	-2.566851
O	6.599493	-4.303754	-2.780535
H	0.483898	-4.554896	0.629509
C	2.143863	-3.331053	-0.048728
C	2.678928	-2.000745	-0.060986
N	3.859402	-1.712087	-0.677281
C	4.528823	-2.709249	-1.281673
C	4.071288	-4.054203	-1.313199
C	2.878638	-4.348701	-0.694301
H	7.478868	-2.461178	-2.922506
C	0.263395	-2.505313	1.212525
C	0.867225	-1.211469	1.157267
H	2.492456	-5.365567	-0.697257
H	5.971265	-1.317628	-1.833772
N	2.027217	-0.958690	0.546825
O	0.197004	-0.225212	1.766099
H	0.676551	0.672015	1.710930

C	0.905826	-3.552681	0.610993
H	-0.683635	-2.617898	1.730382
H	4.664127	-4.803979	-1.817813
N	3.054282	1.802812	0.584760
H	3.385794	4.279031	0.961144
N	4.702191	3.364373	-0.057405
C	4.232599	2.124438	-0.053090
C	6.086751	1.262708	-1.367733
O	6.636138	0.280845	-1.919119
H	1.900247	4.556090	1.923671
N	2.525840	3.949566	1.408712
C	2.198294	2.652302	1.299388
O	1.158636	2.170987	1.812518
C	5.877015	3.582568	-0.719393
N	4.876647	1.089648	-0.673939
H	6.232787	4.610842	-0.702487
H	2.729556	0.810355	0.546667
H	7.515448	2.815993	-1.885281
C	6.585214	2.607677	-1.368734
H	4.480393	0.120005	-0.648640

UPy-NAPyO DADA-ADAD (C_1) [-5653.2]

N	-5.471911	2.204008	0.510278
H	-4.552316	0.201251	-0.710591
N	-3.573536	1.829648	-0.839856
C	-4.311030	2.670189	-0.097476
C	-2.918132	4.473323	-0.437313
O	-2.585258	5.748214	-0.252859
H	-9.720579	0.687864	2.769052
N	-5.419943	-0.034948	-0.220750
C	-6.046322	0.926714	0.484267
O	-7.112446	0.728523	1.113000
C	-2.457831	2.350997	-1.402246
N	-4.032908	3.976571	0.131660
H	-1.863068	1.666149	-2.003454
H	-6.007448	2.883718	1.068838
H	-1.182583	4.076596	-1.691684
C	-2.077557	3.665873	-1.238135
C	-5.824039	8.056843	2.581660
H	-5.835089	-0.957380	-0.239973
C	-5.099852	7.077732	1.802714
H	-9.274277	6.666304	4.367898
H	-8.020362	1.888160	2.053436
N	-5.640612	5.807861	1.749838
O	-4.024129	7.333181	1.187843
H	-10.024116	4.289678	4.100747
C	-6.981710	7.721442	3.222750
H	-5.404523	9.056447	2.627907
H	-5.119988	5.099094	1.189075
H	-3.241868	6.272654	0.341209
N	-8.675279	2.437347	2.645747
C	-9.755062	1.734751	3.119424
O	-10.664105	2.158990	3.836002
H	-7.521512	8.463900	3.806809
C	-7.512158	6.396741	3.145246
C	-6.803158	5.429036	2.381134
N	-7.197246	4.151787	2.232132
C	-8.336440	3.773115	2.848195
C	-9.120746	4.655502	3.633390
C	-8.697032	5.962861	3.772850

AUPy-NAPyO DDAA-AADD (C_1) [-5915.2]

N	-0.990572	-4.591021	-1.773714
C	-0.971754	-5.170483	-3.023439
O	-0.677660	-4.629888	-4.089233
H	0.594794	1.352056	-0.604797
C	-0.071385	-0.716520	-0.517619
C	-0.560638	-1.713437	0.385868
N	-0.835627	-2.964636	-0.129964
C	-0.673574	-3.289501	-1.445125
C	-0.205939	-2.319560	-2.346947
C	0.090641	-1.050529	-1.869232
H	-1.268702	-6.231556	-2.958891
C	0.018484	0.776484	1.374571
C	-0.494513	-0.284715	2.234327
H	0.459735	-0.293568	-2.557778
H	-1.270849	-5.256576	-0.994966

N	-0.772677	-1.525796	1.694049
O	-0.685595	-0.070168	3.470601
H	-1.187251	-3.690547	0.547922
C	0.217667	0.564846	0.044623
H	0.226971	1.734732	1.841788
H	-0.085440	-2.588911	-3.385439
N	-1.944397	-3.502568	3.521340
H	-1.461180	-1.204236	4.469040
N	-2.665503	-5.712638	3.802531
C	-2.140392	-4.752283	2.984561
C	-2.012220	-6.257889	1.200568
O	-1.703875	-6.475841	-0.012231
H	-2.119172	-1.547334	6.086145
N	-1.937230	-1.850901	5.137260
C	-2.241389	-3.125391	4.845699
O	-2.753572	-3.923721	5.670698
C	-2.878096	-6.993831	3.346613
N	-1.816427	-4.974148	1.723014
N	-3.355011	-7.890152	4.258753
H	-1.521586	-2.796365	2.874142
H	-2.706976	-8.276087	1.633034
C	-2.554303	-7.281909	2.038010
H	-2.866841	-5.381606	4.766396
H	-3.819296	-7.544321	5.090834
H	-3.700029	-8.774673	3.906016

AUPy-NAPyO ADDA-DAAD (C_1) [-5919.4]

N	6.031649	0.527001	0.174134
C	7.318119	0.054426	0.215887
O	7.686866	-1.122911	0.206518
H	0.953948	-3.075585	-0.048159
C	2.380428	-1.439770	0.010421
C	2.510756	-0.011936	0.018690
N	3.728486	0.597454	0.073193
C	4.826316	-0.176350	0.116216
C	4.789052	-1.596853	0.107325
C	3.559894	-2.213452	0.055518
H	8.035789	0.893257	0.261313
C	-0.014240	-1.165393	-0.086748
C	0.201222	0.248470	-0.080039
H	3.487469	-3.298785	0.049081
H	5.954448	1.561731	0.192867
N	1.413607	0.806428	-0.028073
O	-0.891906	1.014547	-0.129376
H	-0.688497	2.026447	-0.140179
C	1.073317	-1.994499	-0.042636
H	-1.032814	-1.537709	-0.128732
H	5.715820	-2.152063	0.142475
N	3.903491	3.663618	0.101253
H	4.630457	6.081691	0.125996
N	2.757809	5.721769	0.012559
C	2.736491	4.399997	0.021006
C	0.327193	4.295421	-0.127216
O	-0.708995	3.566579	-0.180857
H	6.390653	5.786481	0.242853
N	5.436068	5.455567	0.187131
C	5.228923	4.125623	0.183486
O	6.155823	3.289533	0.250346
C	1.555311	6.378963	-0.070843
N	1.586698	3.675187	-0.046210
H	2.529926	8.171461	0.117560
H	3.819797	2.629532	0.097102
H	-0.607018	6.236216	-0.201526
C	0.336820	5.705917	-0.138754
H	1.599066	2.623031	-0.034972
N	1.644307	7.737966	-0.111732
H	0.809076	8.299263	-0.008680

AUPy-NAPyO DADA-ADAD (C_1) [-5921.7]

N	-5.850212	0.572104	-0.120202
C	-7.158877	0.175741	-0.232893
O	-7.584131	-0.978702	-0.317041
H	-0.936009	-3.275521	0.062819
C	-2.304249	-1.590704	0.034579
C	-2.395405	-0.171613	0.082417
N	-3.555020	0.508257	0.031346
C	-4.695193	-0.205549	-0.073292

C	-4.718772	-1.621774	-0.129894
C	-3.516631	-2.300244	-0.074378
H	-7.830443	1.052696	-0.240959
C	0.117332	-1.426331	0.202194
C	0.031306	0.016826	0.249387
H	-3.497267	-3.386616	-0.114549
H	-5.706588	1.600856	-0.062503
N	-1.236749	0.563198	0.186653
O	1.040843	0.771504	0.342573
H	-5.666612	-2.134455	-0.214036
C	-1.008623	-2.190469	0.098575
H	1.110387	-1.860887	0.251876
H	-1.300840	1.606231	0.219251
N	-3.510094	3.624698	0.163136
H	-4.135405	6.064027	0.203689
N	-2.280731	5.626583	0.304881
C	-2.287420	4.289497	0.255146
H	0.941338	2.356961	0.379316
O	1.112328	3.364247	0.417414
H	-5.909108	5.843208	0.089324
N	-4.968595	5.474150	0.138918
C	-4.809650	4.136036	0.103050
O	-5.775306	3.339861	0.019432
C	-1.066700	6.234498	0.390824
N	-1.205446	3.480094	0.286838
H	-1.957124	8.067832	0.570427
H	-3.471562	2.596535	0.126627
H	1.095078	5.960025	0.502885
C	0.119216	5.492211	0.432971
C	-0.003292	4.101419	0.377485
N	-1.075199	7.600484	0.397611
H	-0.234381	8.097651	0.662250

UPy-AUPy DADA-ADAD (C_1) [-5254.8]

N	-0.579015	-2.213740	0.042429
H	-3.090867	-2.372429	0.099323
N	-2.315416	-0.623670	0.067729
C	-1.003188	-0.885405	0.036418
H	1.501205	1.890559	-0.050515
O	0.556074	2.271460	-0.037261
H	-3.197862	-4.161001	0.114402
N	-2.663562	-3.302075	0.103428
C	-1.321261	-3.387545	0.069869
O	-0.721281	-4.496566	0.064086
C	-2.680836	0.686964	0.056830
N	-0.001202	0.019519	-0.000323
N	-4.023855	0.939206	0.045724
H	0.443095	-2.365547	0.014963
H	-1.998657	2.759057	0.014564
C	-1.725630	1.709487	0.022102
C	-0.383843	1.319516	-0.005702
N	2.923042	-0.626881	-0.069708
H	5.437902	-0.478243	-0.139738
N	4.664097	-2.220435	-0.120823
C	3.349163	-1.949784	-0.080122
H	0.826243	-4.731952	0.009341
O	1.779855	-5.108303	-0.023325
H	5.551588	1.310550	-0.142091
N	5.013698	0.453776	-0.126708
C	3.672100	0.547124	-0.090994
O	3.074249	1.654925	-0.075371
C	5.003099	-3.531155	-0.129538
N	2.350358	-2.864088	-0.046364
H	6.069123	-3.747438	-0.162750
H	1.899126	-0.470306	-0.042627
H	4.350561	-5.595677	-0.106084
C	4.072282	-4.547807	-0.098847
C	2.713082	-4.159510	-0.055688
H	-4.350105	1.876026	0.246362
H	-4.649542	0.177818	0.280308

UPy-AUPy DDAA-AADD (C_1) [-5255.5]

N	1.392225	-0.028209	-0.216969
H	-0.302083	1.859913	-0.247371
N	1.926480	-2.295827	0.074551
C	2.344379	-1.016514	-0.153302
C	4.589165	-1.690988	-0.235140

O	5.806321	-1.400279	-0.384412
H	-1.722893	0.811502	-0.048699
N	-0.721046	0.907539	-0.161893
C	0.001468	-0.221558	-0.079433
O	-0.500923	-1.357777	0.096894
C	2.835380	-3.317048	0.159910
N	3.621208	-0.692894	-0.311107
H	2.422735	-4.302733	0.344024
H	1.754320	0.937887	-0.371555
H	4.901716	-3.842740	0.078802
C	4.158579	-3.056097	0.015799
H	0.902908	-2.408748	0.166248
N	4.600810	2.037827	-0.963446
H	6.316164	0.172420	-0.877333
N	4.065904	4.303200	-1.218436
C	3.646402	3.031729	-0.943958
C	1.443064	3.752145	-0.638896
O	0.239080	3.465803	-0.372758
H	7.692272	1.184663	-1.353281
N	6.699031	1.090941	-1.179921
C	5.967883	2.216863	-1.244379
O	6.451523	3.338779	-1.536869
C	3.180217	5.357334	-1.206539
N	2.395788	2.721088	-0.664176
N	3.711480	6.596158	-1.437122
H	4.254778	1.083921	-0.731114
H	1.125517	5.892328	-0.901374
C	1.859656	5.094391	-0.919046
H	5.080961	4.382795	-1.417719
H	3.060160	7.346097	-1.636815
H	4.599123	6.660411	-1.923073

UPy-DAN DADA-ADAD (C_1) [-6103.1]

N	-2.309037	-0.194375	0.142664
C	-2.697959	1.093515	0.413640
O	-1.981924	2.030861	0.771657
H	3.605388	-2.112515	-0.084011
C	1.447393	-1.951293	0.020287
C	0.225091	-2.701145	0.000957
N	-0.993347	-2.087553	0.046845
C	-1.028635	-0.751026	0.148986
C	0.136181	0.065450	0.224311
C	1.363641	-0.544687	0.145121
H	-3.788745	1.204888	0.278105
C	2.658119	-4.030876	-0.183283
C	1.402284	-4.701918	-0.146341
H	2.277504	0.044207	0.173386
H	-3.076726	-0.826780	-0.156243
N	0.225265	-4.064744	-0.063865
H	1.904686	-8.044793	-0.314404
H	0.405370	-6.503653	0.125922
C	2.666424	-2.661278	-0.085662
O	3.459168	-6.796356	-0.778621
C	2.296977	-7.018912	-0.433280
N	1.319451	-6.095503	-0.158328
H	0.026752	1.136406	0.323892
H	3.566359	-4.610960	-0.268938
N	-3.548738	-3.676609	-0.265332
H	-5.943875	-4.448201	-0.481009
N	-4.650487	-5.737160	0.066458
C	-3.532725	-5.025815	0.015753
C	-2.121009	-6.932490	0.544704
O	-0.964513	-7.382745	0.723268
H	-6.649065	-2.868500	-0.937143
N	-5.855437	-3.455190	-0.712964
C	-4.649780	-2.868799	-0.608239
O	-4.456236	-1.650325	-0.811755
C	-4.528319	-7.064398	0.365846
N	-2.299733	-5.573341	0.237301
H	-5.464348	-7.618539	0.398146
H	-2.651207	-3.157406	-0.196777
H	-3.277007	-8.747239	0.850481
C	-3.336248	-7.690505	0.615206
H	-1.432883	-4.991306	0.143940

AUPy-DAN DDAA-AADD (C_1) [-6372.0]

N	-2.319638	-0.201359	0.145075

C	-2.712400	1.085498	0.413111
O	-1.999816	2.026263	0.769965
H	3.599378	-2.113559	-0.043948
C	1.440311	-1.953047	0.040607
C	0.218720	-2.703758	0.005750
N	-1.000869	-2.091505	0.046246
C	-1.037933	-0.755892	0.155640
C	0.125349	0.061526	0.243526
C	1.354200	-0.547276	0.171784
H	-3.803375	1.193479	0.275923
C	2.653697	-4.031580	-0.162868
C	1.397316	-4.703674	-0.143544
H	2.267186	0.042423	0.211138
H	-3.085556	-0.836648	-0.152670
N	0.220147	-4.066130	-0.068899
H	1.897986	-8.044173	-0.326774
H	0.388823	-6.509176	0.094663
C	2.660584	-2.662547	-0.057538
O	3.465252	-6.795711	-0.745714
C	2.294321	-7.017953	-0.428167
N	1.312280	-6.096379	-0.167429
H	0.014051	1.131886	0.347489
H	3.562926	-4.610980	-0.242797
N	-3.562736	-3.691538	-0.283400
H	-5.951867	-4.465144	-0.549890
N	-4.652205	-5.760770	0.001077
C	-3.538957	-5.050103	-0.030583
C	-2.132614	-6.963887	0.461409
O	-0.962782	-7.399893	0.643021
H	-6.654589	-2.879666	-0.983012
N	-5.862980	-3.468295	-0.757609
C	-4.659036	-2.881623	-0.624375
O	-4.469986	-1.657906	-0.801938
C	-4.544875	-7.104759	0.264015
N	-2.306570	-5.588596	0.182798
H	-6.586674	-7.258586	0.254870
H	-2.666825	-3.173279	-0.203288
H	-3.253521	-8.787990	0.714112
C	-3.322633	-7.725791	0.506135
H	-1.443815	-5.004471	0.106765
N	-5.724573	-7.789522	0.239274
H	-5.758189	-8.736149	0.595071

References

[1] M. L. Pellizzaro, S. A. Barrett, J. Fisher and A. J. Wilson, *Organic & Biomolecular Chemistry* **2012**, *10*, 4899-4906.

[2] M. L. Pellizzaro, K. A. Houton and A. J. Wilson, *Chem. Sci.* **2013**, *4*, 1825-1829.

[3] a) ADF2017, SCM, Theoretical Chemistry, Vrije Universiteit, Amsterdam, The Netherlands, http://www.scm.com; b) G. te Velde, F. M. Bickelhaupt, E. J. Baerends, C. Fonseca Guerra, S. J. A. van Gisbergen, J. G. Snijders and T. Ziegler, *J. Comput. Chem.* **2001**, *22*, 931-967; c) C. Fonseca Guerra, J. G. Snijders, G. te Velde and E. J. Baerends, *Theor. Chem. Acc.* **1998**, *99*, 391-403.

[4] A. D. Becke, *Phys. Rev. A* **1988**, *38*, 3098-3100.

[5] C. Lee, W. Yang and R. G. Parr, *Physical Review B* **1988**, *37*, 785-789.

[6] S. Grimme, J. Antony, S. Ehrlich and H. Krieg, *J. Chem. Phys.* **2010**, *132*, 154104.

[7] E. R. Johnson and A. D. Becke, *J. Chem. Phys.* **2005**, *123*, 0241011-0241017.

[8] S. Grimme, S. Ehrlich and L. Goerigk, *J. Comput. Chem.* **2011**, *32*, 1456-1465.

[9] Brauer, M. K. Kesharwani, S. Kozuch and J. M. L. Martin, *Phys. Chem. Chem. Phys.* **2016**, *18*, 20905-20925; b) C. Fonseca Guerra, T. van der Wijst, J. Poater, M. Swart and F. M. Bickelhaupt, *Theor. Chem. Acc.* **2010**, *125*, 245-252.

[10] A. D. Becke, *J. Chem. Phys.* **1988**, *88*, 2547-2553.

[11] E. J. Baerends, D. E. Ellis and P. Ros, *Chem. Phys.* **1973**, *2*, 41-51.

[12] M. Franchini, P. H. T. Philipsen, E. van Lenthe and L. Visscher, *J. Chem. Theory Comp.* **2014**, *10*, 1994-2004.

[13] C. C. Pye and T. Ziegler, *Theor. Chem. Acc.* **1999**, *101*, 396-408.

[14] S. K. Wolff, *Int. J. Quantum Chem* **2005**, *104*, 645-659.

[15] C. Fonseca Guerra, J.-W. Handgraaf, E. J. Baerends and F. M. Bickelhaupt, *J. Comput. Chem.* **2004**, *25*, 189-210.
